# Traction Force Microscopy for Viscoelastic Substrates: A Semi‐Analytical Method

**DOI:** 10.1002/advs.202522252

**Published:** 2026-05-28

**Authors:** Adrià Villacrosa‐Ribas, Daniëlle C. A. Duffhues, Pim van den Bersselaar, Sarah Pragnere, Bart G. W. Groenen, Mariana Azevedo Gonzalez Oliva, Giuseppe Ciccone, Manuel Salmeron‐Sanchez, Carlijn V. C. Bouten, Jose J. Muñoz, Vito Conte

**Affiliations:** ^1^ Department of Biomedical Engineering Eindhoven University of Technology (TU/e) Eindhoven Netherlands; ^2^ Institute For Complex Molecular Systems (ICMS) Eindhoven University of Technology (TU/e) Eindhoven Netherlands; ^3^ Institute For Bioengineering of Catalonia (IBEC) The Barcelona Institute of Science and Technology (BIST) Barcelona Spain; ^4^ Institució Catalana de Recerca i Estudis Avançats (ICREA) Barcelona Spain; ^5^ Centre For the Cellular Microenvironment (CeMi) University of Glasgow Glasgow UK; ^6^ Department of Mathematics Polytechnic University of Catalonia (UPC) Barcelona Spain; ^7^ Centre Internacional de Mètodes Numèrics en Enginyeria (CIMNE) Barcelona Spain; ^8^ Institut De Matemàtiques De La UPC – BarcelonaTech (IMTECH) Barcelona Spain

**Keywords:** cell–material interactions, semi‐analytical methods, stress relaxation, viscoelastic hydrogels, viscoelastic traction force microscopy

## Abstract

Traction force microscopy (TFM) quantifies cellular forces at the cell–extracellular matrix interface, yet elastic formulations neglect viscous dissipation and can misinterpret cellular forces on viscoelastic substrates. We introduce a semi‐analytical 2D viscoelastic TFM (veTFM) that generalizes the Boussinesq framework of elastic TFM to Generalized Maxwell (GMX) substrates with one or two components. By combining Fourier and Laplace transforms, veTFM quantifies time‐resolved tractions in finite‐thickness substrates and resolves stress‐free reference and substrate pre‐stress. We derive criteria for when elastic regimes remain valid in this framework. This positions veTFM as a scalable extension of standard 2D TFM (eTFM) to viscoelastic substrates, identifying when eTFM remains sufficient, which elastic limit applies, and when full viscoelastic quantification is required. Applied to beating cardiomyocytes, epithelial cells, and dermal fibroblasts cultured on linear‐polyacrylamide and alginate viscoelastic hydrogels, veTFM shows that the elastic or viscoelastic regime engaged by the cell depends on timescale matching between the loading rate and the substrate's relaxation times. Notably, for the Generalized Maxwell substrates analyzed here, viscoelastic traction magnitudes scale with the substrate's total dissipation rather than individual relaxation times, with total dissipation setting traction magnitude and timescale matching determining whether the cell engages the substrate in an elastic or viscoelastic regime.

## Graphical abstract

A semi‐analytical viscoelastic traction force microscopy framework is introduced for quantifying time‐resolved cell tractions on flat finite‐thickness substrates. The method generalizes elastic traction force microscopy to Generalized Maxwell materials, identifies when elastic approximations remain valid and, when they do not, shows that inferred traction dynamics depend on the spectral overlap between cellular loading and substrate rheology across distinct human cell systems.

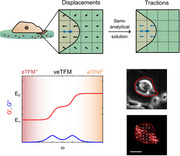

## 1 Introduction

Traction force microscopy (TFM) has become a cornerstone method in mechanobiology by enabling quantification of the forces that cells transmit to the extracellular matrix (ECM). The widespread use of its standard 2D formulation has been driven largely by computationally efficient elastic algorithms that apply Fourier‐domain Green's functions derived from the Boussinesq solution for flat substrates of finite thickness [1, 2]. Notably, their speed and accessibility stem from their semi‐analytical structure rather than from elasticity per se. Extending TFM to full 3D settings generally requires volumetric imaging and finite element modeling (FEM), although micro‐engineered substrate geometries can improve scalability by standardizing 3D traction quantification while controlling cell shape [3]. In many experimental settings, the conventional elastic framework remains entirely appropriate, particularly when the substrate behaves effectively elastically over the timescales of cellular loading. However, these elastic formulations do not account for viscous dissipation, which is relevant in many biological tissues and biomaterials [4, 5, 6, 7] and can affect traction quantification when substrate dissipation is non‐negligible over the timescales of cellular loading [8, 9]. While numerical approaches such as FEM can incorporate viscoelasticity, their computational cost limits their use in routine or high‐throughput settings and may hinder their integration into large‐scale automated or AI‐driven analysis workflows. This limitation has become increasingly important because viscoelastic matrices are being adopted more broadly across mechanobiology and biomaterials research [5, 6, 7, 8, 9, 10, 11, 12, 13].

Yet quantitative traction readouts on such substrates have lagged behind displacement‐based analyses because routine viscoelastic traction quantification tools have not been readily available. Existing approaches for traction analysis on viscoelastic substrates include FEM‐based implementations [14], forward or empirical formulations [15], and analytical solutions developed for specific constitutive models or geometries not directly applicable to standard cellular TFM settings [16]. However, these approaches have not provided a routine inverse semi‐analytical counterpart to standard 2D elastic TFM for flat finite‐thickness viscoelastic substrates, in part because they involve greater computational cost, added experimental complexity, or assumptions that do not map directly onto common TFM workflows. By contrast, the analytical Boussinesq framework remains the preferred basis for standard 2D elastic TFM (eTFM) because of its speed, precision, and ease of use [17].

Here, we introduce a semi‐analytical method for 2D viscoelastic TFM (veTFM) that generalizes mainstream 2D eTFM to flat finite‐thickness substrates modeled as linear Generalized Maxwell (GMX) materials with one or two viscoelastic components—a discrete‐relaxation class widely used to describe many biological tissues and biomaterials [8, 18, 19]. By combining Fourier and Laplace transforms, the method quantifies time‐resolved cellular tractions from the same primary experimental observable used in conventional eTFM—namely, the displacement field at the cell‐facing substrate surface—while preserving a computationally efficient semi‐analytical structure. In this sense, veTFM is not intended as a niche mechanics variant, but as a practical generalization of standard traction quantification that can be integrated into routine analysis pipelines. The framework is compatible with any 2D TFM workflow on flat finite‐thickness continuum substrates, irrespective of whether the displacement field is obtained from a stress‐free reference configuration or from a reference‐free approach [20, 21], and irrespective of the microscopy modality used to measure displacements at the cell‐substrate interface [2, 21, 22, 23, 24, 25, 26]. It is also not restricted to a specific substrate chemistry but applies in principle to any substrate that can be described as a linear viscoelastic continuum in the relevant regime, including elastomeric materials, such as silicone or polydimethylsiloxane (PDMS), provided their viscoelastic response is characterized appropriately.

Rather than replacing eTFM universally, veTFM addresses two practical ambiguities that arise when cells are studied on viscoelastic substrates. First, when an elastic approximation is sufficient, veTFM provides a principled basis for determining which elastic limit is appropriate, rather than assuming a single stiffness a priori. Second, it identifies regimes in which no single elastic stiffness provides a reliable approximation because the cell probes different parts of the substrate rheology over time. veTFM can therefore serve as a practical default for viscoelastic substrates—reducing to an effectively elastic traction quantification when viscous contributions are negligible over the relevant loading‐frequency range, while remaining valid when they are not. Because the method preserves the semi‐analytical structure of standard 2D TFM, it also remains computationally practical for routine use and scalable analysis. More broadly, the growing use of viscoelastic matrices in mechanobiology has outpaced the availability of routine traction‐based readouts, and veTFM is intended to close that gap by extending standard displacement‐to‐traction workflows to substrates for which dissipation cannot be neglected [5, 13].

We validate the method through theoretical consistency checks and benchmarking against independent analytical and custom FEM‐based solutions. In parallel, we address practical experimental challenges—namely, identification of an appropriate stress‐free reference configuration and correction for substrate pre‐stress—that are particularly relevant in viscoelastic settings. We further derive quantitative criteria that delineate when semi‐analytical elastic TFM approximations remain valid and when the full viscoelastic formulation is required. We apply veTFM to three human cell types with distinct loading dynamics in vitro—beating cardiomyocytes, mammary epithelial cells, and dermal fibroblasts cultured on linear polyacrylamide and alginate hydrogels. Our results show that the effective mechanical response sampled by cells on viscoelastic substrates depends on the rate and temporal content of substrate deformation: rapid loading samples a stiffer, high‐frequency response; slow loading a softer, low‐frequency response; and intermediate regimes produce force distributions that diverge from any single elastic approximation. These effects cannot be captured by a single elastic stiffness value. veTFM therefore provides a unified, rate‐dependent framework for quantifying cell‐generated forces on viscoelastic substrates.

This time‐dependent modulation of traction forces, which is absent from purely elastic models, is consistent with prior kinematic and biological observations that have suggested rate‐dependent cell–matrix interactions [27]. It also motivates the question of which viscoelastic material properties most strongly influence traction magnitude. Within the GMX2 framework analyzed here, our results show that dissipation is a more important determinant of cell traction magnitude than any individual relaxation time, refining mechanobiological interpretations that emphasize material timescales [8, 28, 29]. More broadly, our results suggest that the viscoelastic regime engaged by the cell is jointly determined by substrate dissipation and by the overlap between material relaxation and the frequency spectrum of cell‐induced substrate deformation. In this framework, substrate behavior may be effectively elastic or genuinely viscoelastic depending on the loading spectrum probed by the cell, which in turn determines whether an elastic approximation is sufficient or whether full viscoelastic quantification is required.

Taken together, veTFM provides a practical force readout for viscoelastic matrices that resolves stiffness‐selection ambiguity, identifies regimes in which elastic quantification is unreliable, and captures history‐dependent and local traction features that can be missed even when elastic and viscoelastic quantifications yield similar median traction magnitudes. This extends mainstream 2D traction microscopy to the growing range of mechanobiology, biomaterials, and regenerative engineering settings in which substrate dissipation cannot be ignored [4, 5, 8, 9, 12, 13, 27, 30, 31, 32, 33].

## 2 Results

### 2.1 Semi‐Analytical Problem Formulation and Solution of the 2D TFM on Flat Viscoelastic Substrates With Finite Thickness

**FIGURE 1:**
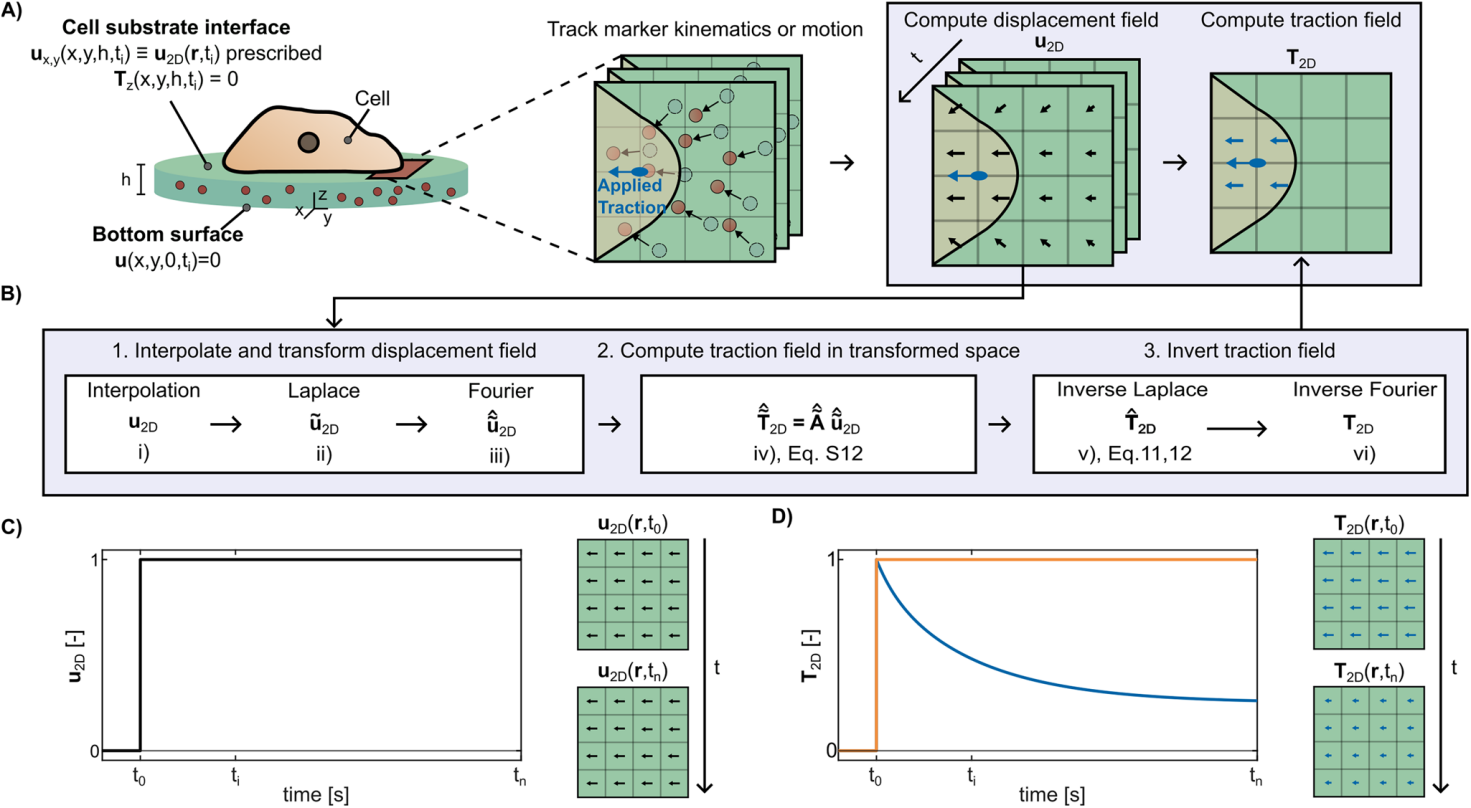
The veTFM approach. (A,B) Schematic of the 2D traction force microscopy (2D TFM) setup. (A) The substrate (green flat cylinder, left subpanel) is flat with finite thickness *h* and attached to a fixed boundary (usually glass) at the bottom surface (*z* = 0), which is numerically emulated by enforcing zero displacement ***u*** (*x*, *y*, *0*, *t*) = 0. Cells are seeded on and adhere to the substrate's top surface (cell‐substrate interface, *z* = *h*). Cells deform this surface by exerting an unknown two‐dimensional traction field ***T***_2D_ that generates a measurable displacement field ***u***_2D_ in the *x* − *y* plane of the cell‐substrate interface. The ***u***_2D_ is experimentally determined via image analysis and tracking fiducial markers embedded in the substrate over discrete timepoints *t* = *t*_0_, *t*_1_,···, *t_n_*. Out‐of‐plane tractions are assumed negligible in 2D TFM (i.e., *T*_z_(*x*, *y*, *h*, *t*) = 0), and the unknown ***T***_2D_ is inferred by solving mechanical equilibrium (quasi‐static approximation of cellular motion) across displacement fields ***u***_2D_ for *t* = *t*_0_, *t*_1_,···, *t_n_*. (B) Overview of the key steps in the veTFM algorithm used to compute traction fields at the generic timepoint *t* from time‐lapse displacement data for *t* = *t*_0_, *t*_1_,···, *t_n_*. (B1) The continuous displacement field ***u***_2D_ is constructed in the *Oxyz*‐space by interpolating measurements on fiducial markers positions at *t* = *t*_0_, *t*_1_,···, *t*_n_, and is then transformed to the Fourier–Laplace space (Equations (13) and (14)). (B2) In the Fourier–Laplace space, traction fields ${\hat{\tilde{T}}}_{2D}$ are obtained from displacement fields via an algebraic expression derived from viscoelastic theory (Equation (3)). (B3) Traction fields ***T***_2D_ are finally recovered in the original *Oxyz*‐space by performing the inverse Laplace and Fourier transforms (Methods, Section S3). (C,D) Illustrative conceptualization of the mechanical response of the viscoelastic‐substrate in a simple case. (C) A sudden step‐like uniform displacement field ***u***_2D_ is applied at the cell‐substrate interface; namely, ***u***_2D_ = *H*(*t*)*u*_0_ ***e**_x_* with *H*(*t*) the Heaviside's step function and ***e**_x_* the unit vector of the *x*‐axis. (D) Traction field corresponding to the step‐like displacement field ***u***_2D_ = *H*(*t*)*u*_0_ ***e**_x_* applied at the cell‐substrate interface in the case of: an elastic substrate (orange line) and viscoelastic substrate (blue line).

To compute cellular traction fields at a generic time *t*, we consider a flat viscoelastic substrate of finite thickness *h*, represented as a semi‐infinite half‐space within a Cartesian coordinate system (*x*, *y*, *z*), with the vertical dimension ranging from the fixed bottom (*z* = 0) to the top surface (*z* = *h*). We assume that cells adhere and immediately deform the top surface at time *t*_*A*_ (adhesion time), inducing an unknown traction force field ***T*** (*x*, *y*, *h*, *t*) and a resultant displacement field ***u*** (*x*, *y*, *z*, *t*) throughout the substrate domain (Figure 1A). For times prior to cell attachment *t* < *t*_A_, the substrate remains undeformed and stress‐free (i.e., ***T*** = 0 and ***u*** = 0), whereas for times *t* ≥ *t*_*A*_, the in‐plane displacements *u_i_* (*x*, *y*, *h*, *t*) with *i* = *x*, *y* at the cell‐substrate interface can be experimentally measured [34, 35, 36].

Under the Quasi‐Static Approximation of Cellular Motion, Mechanical Equilibrium in the Substrate is Enforced by Cauchy's Partial Differential Equations

(1) $$\nabla \cdot \sigma \left(x,y,z,t\right)=0\;\left(0<z<h\right)$$

subject to a fixed substrate at its bottom ***u*** (*x*, *y*, 0, *t*) = 0.

Cellular Traction Forces Relate to the Cauchy Stress Tensor **σ** via the Boundary Condition at *z* = *h*

(2) $$T\left(x,y,h,t\right)=\sigma \left(x,y,h,t\right)\cdot n$$

with ***n*** assumed as the external unit vector normal to the flat undeformed top surface of the substrate [37] and the viscoelastic stress tensor **σ** constitutively defined by two functions *Φ*(*t*) and *Ψ*(*t*) (Equation (S7) and Figure 1C,D) which are experimentally determined [38] (Experimental Section and Section S2). In 2.5D TFM, the 3D problem in Equations (1) and (2) is solved in the substrate domain with quantification of the full traction field ***T*** = [*T_x_* (*x*, *y*, *h*, *t*), *T_y_* (*x*, *y*, *h*, *t*), *T_z_* (*x, y*, *h*, *t*)] from the displacement field ***u*** = [*u_x_* (*x*, *y*, *h*, *t*), *u_y_* (*x*, *y*, *h*, *t*), *u_z_* (*x*, *y*, *h*, *t*)] at the cell‐substrate interface *z* = *h* (Section S4). In standard 2D TFM, the same 3D problem is solved under an in‐plane traction field approximation at the cell–matrix interface, in which the out‐of‐plane traction component *T_z_* is assumed negligible relative to the in‐plane components *T_x_* and *T_y_*, namely *T_z_* (*x*, *y*, *h*, *t*) = 0. Under this approximation, 2D TFM therefore quantifies the traction field at the cell‐substrate interface as ***T*** = [*T_x_* (*x*, *y*, *h*, *t*), *T_y_* (*x*, *y*, *h*, *t*), 0]. As the developments below focus on the 2D TFM formulation, and with and the assumption *z* = *h* implied throughout the analysis, we use the shorthand notations ***T***_2D_ = [*T_x_* (***r***, *h*, *t*), *T_y_* (***r***, *h*, *t*)] and ***u***_2D_ = [*u_x_* (***r***, *h*, *t*), *u_y_* (***r***, *h*, *t*)] for the traction and displacement fields ***T*** and ***u*** in the 2D TFM setting, with ***r*** = (*x*, *y*) the position vector at the cell‐substrate interface. To simplify the integro‐differential equations in Equations (1), (2) and Equation (S6), we apply a Laplace transform to ***u*** in the time variable *t* (Figure 1B, box 1‐ii) to obtain $\tilde{u}=\tilde{u}\left(r,z,s\right),$ where *s* is the complex Laplace domain variable, followed by a 2D Fourier transform in the top *x* − *y* plane (Figure 1B,iii) to obtain $\hat{\tilde{u}}=\hat{\tilde{u}}\left(k,z,s\right)$, where ***k*** = (*k_x_*, *k_y_*) is the Fourier wavevector. The integro‐differential problem in Equations (1), (2) and Equation (S6) reduces to an ordinary differential equation along the vertical coordinate *z* (Figure 1B, box 2‐iv), whose solution is a relationship between the Fourier–Laplace transforms of traction ${\hat{\tilde{T}}}_{2D}={\hat{\tilde{T}}}_{2D}\left(k,s\right)$ and displacement ${\hat{\tilde{u}}}_{2D}$ fields defined as

(3) $${\hat{\tilde{T}}}_{2D}=\hat{\tilde{A}}\;{\hat{\tilde{u}}}_{2D}$$

with $\hat{\tilde{A}}=\hat{\tilde{A}}\left(k,s\right)$ the viscoelastic Green's function (Experimental Section and Equation (S12)). Real‐space traction fields ***T***_2D_ are finally recovered by inverse Fourier and Laplace transformations (Figure 1B, box 3‐v, vi), as detailed in subsequent sections.

### 2.2 Discrete Numerical Implementation of the Fourier–Laplace veTFM Solution for Viscoelastic Substrates

The general Fourier–Laplace formulation described in Equation (3) assumes displacement fields are continuously available both in space and time. In practice, however, experimental displacement fields ***u***_2D_ are sampled on a discrete spatial grid and acquired at discrete time points $t=t_{0},\cdots ,t_{i},\cdots ,t_{n}$ (e.g., via time‐lapse microscopy imaging) rather than as continuous functions over the time interval [*t*_0_, *t_n_*] and the spatial domain considered. It is worth noticing that experimental constraints may further impose that the first measured displacement time point *t*_0_ may follow the time point of cellular‐adhesion *t*_A_ (i.e., *t*_0_ ≥ *t*_A_). As a result, the analytical spatial‐temporal dependence of the displacement field required for computing its joint Fourier–Laplace transform ${\hat{\tilde{u}}}_{2D}$ may not be directly available. To address this, the continuous Fourier transform used in Equation (3) is replaced with a 2D discrete Fourier transform (2D‐DFT), consistent with elastic TFM approaches [1, 2] (Experimental Section). Given the discrete temporal sampling of displacement fields in time increments, piecewise linear interpolation is applied between experimentally measured displacement timepoints, thus enabling numerical approximation of the Laplace transform (Experimental Section). Following this discrete numerical approach, the traction field in the Fourier–Laplace domain ${\hat{\tilde{T}}}_{2D}$ is calculated from Equation (3). Real‐space traction fields ***T***_2D_ are subsequently recovered by numerically implementing the inverse transforms. Specifically, the inverse time Laplace transform of ${\hat{\tilde{T}}}_{2D}$ is numerically carried out according to the specific viscoelastic model chosen for the substrate (see Section 2.5) to obtain ${\hat{T}}_{2D}$, followed by its spatial inverse 2D discrete Fourier transform (2D‐IDFT) to obtain ***T***_2D_ (Experimental Section).

### 2.3 Formulation of veTFM for a Two‐Component Generalized Maxwell Model

The general veTFM solution provided by Equation (3) requires specifying a particular viscoelastic constitutive model to compute cellular traction fields from displacement data. By fitting experimental stress relaxation data from the hydrogel materials used in our case studies (Experimental Section and Section S2), we found that two relaxation times best describe the viscoelastic behavior of our substrates—consistent with the GMX2 model commonly adopted for soft biological tissues and viscoelastic hydrogel substrates in other applications [8, 18, 19]. Notwithstanding, our method is also available and applicable to the GMX1 case (Section S3), relevant for viscoelastic substrates with a single characteristic relaxation timealso previously adopted [39].

**FIGURE 2:**
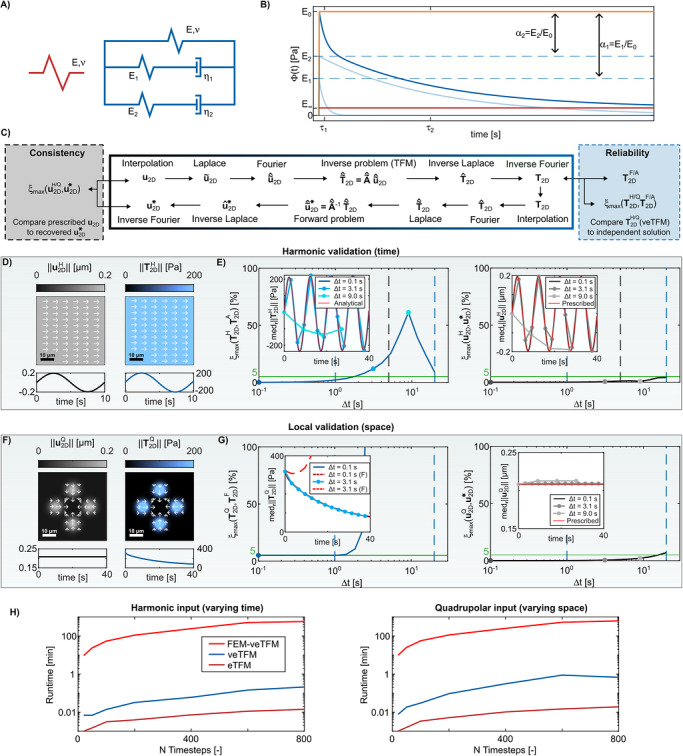
Validation of the veTFM solution for a two‐component deviatoric Generalized Maxwell model. (A) Symbolic representation of linear elastic (red) and linear viscoelastic material models (blue). Continuum elastic materials are fully characterized by their Young's modulus or stiffness *E* and Poisson's ratio *ν*. Linear viscoelastic materials described by a two‐component Generalized Maxwell model (GMX2) are fully characterized by the parameters *E* and *ν* of the elastic branch arranged in parallel with two viscoelastic Maxwell elements, each defined by its own stiffness *E_i_* and viscosity *η*_*i*_ (with *i* = 1, 2). (B) Illustrative diagram of stress‐relaxation behavior for a viscoelastic deviatoric GMX2 substrate (dark‐blue curve, Methods, Equation (15)) with: instantaneous and terminal stiffness *E*_0_ and *E*_∞_, respectively (blue dashed‐lines, Equations (4) and (5)), dissipations *α*_*i*_ and *i* = 1, 2 (Equation (6)) and characteristic times *τ*_*i*_ and *i* = 1, 2 (Equation (7)); an elastic substrate with stiffness *E*_0_ (orange line, Equation (4)) and *E*_∞_ (red line, Equation (5)), respectively. (C) Schematic of the two pillars of the veTFM validation framework: reliability and consistency. Reliability (right): a known displacement field ***u***_2D_ is imposed on the substrate as either varying in time or in space; the corresponding traction field ***T***_2D_ is computed via veTFM (involving numerical Laplace inversion) and compared to an independent reference solution that is either analytical or derived from finite element modeling (FEM). Consistency: the traction field ***T***_2D_ is imposed on the substrate and the resulting displacement field $u_{2D}^{*}$ is computed and compared to the original prescribed displacement field ***u***_2D_. (D) Spatial profile (top, illustrative resolution) and time evolution (bottom) of the median magnitude for the harmonic displacement field $u_{2D}^{H}$ and the corresponding traction field $T_{2D}^{H}$. (E) **Left**: maximum absolute differences between $T_{2D}^{H}$ provided by veTFM across different sampling intervals Δ*t* (dark‐blue line) and the analytical traction field $T_{2D}^{A}$ for a prescribed harmonic displacement input $u_{2D}^{H}$. **Inset**: representative trajectories of median traction magnitudes provided by veTFM over time for sampling intervals Δ*t* = 0.1*s*; 3.1*s*; 9.0*s* (blue lines); and by $T_{2D}^{A}$ (red line). **Right**: maximum absolute differences between the prescribed $u_{2D}^{H}$ and the recovered harmonic displacement field $u_{2D}^{*}$ obtained from $T_{2D}^{H}$ when using veTFM. **Inset**: representative trajectories of median displacement magnitude of $u_{2D}^{H}$ (red line) and those obtained by veTFM over time for sampling intervals Δ*t* = 0.1*s*; 3.1*s*; 9.0*s* (gray lines). (F) Spatial profile (top, illustrative resolution) and time evolution (bottom) of the median magnitude for the quadrupolar displacement field $u_{2D}^{Q}$ over four localized regions of the substrate's top surface and the corresponding traction field $T_{2D}^{Q}$. For the displacement field, magnitude varies with a Gaussian profile over space for each region to ensure continuity and smoothness with the rest of the surface. (G) **Left**: maximum magnitude difference between $T_{2D}^{Q}$ provided by veTFM across different sampling intervals Δ*t* (dark‐blue line) and the FEM algorithm $T_{2D}^{F}$ for a prescribed quadrupolar displacement input $u_{2D}^{Q}$. **Inset**: representative trajectories of average traction magnitude provided by veTFM over time for sampling intervals Δ*t* = 0.1*s* and 3.1*s* (full blue lines) and by $T_{2D}^{F}$ (dashed red lines). **Right**: maximum absolute differences between the prescribed $u_{2D}^{Q}$ and the recovered quadrupolar displacement field $u_{2D}^{*}$ obtained from $T_{2D}^{Q}$ when using veTFM. **Inset**: representative trajectories of median displacement magnitude of $u_{2D}^{Q}$ (red line) and those obtained by veTFM over time for sampling intervals Δ*t* = 0.1*s*; 3.1*s*; 9.0*s* (grey lines). (E,G) Vertical dashed blue lines indicate relaxation times *τ*_1_ < *τ*_2_, while the vertical black dashed line marks the time (2*f*)^−1^ with *f* = 1/10 Hz for the harmonic case, i.e., the maximum sampling interval ensuring quantification of the temporal dependence of $u_{2D}^{H}$. N.B. All simulations assume a substrate with material properties: *E* = 700 Pa, *ν* = 0.4, *E*_1_ = 500 Pa, *E*_2_ = 4000 Pa, *η*_1_ = 500 Pa · s, *η*_2_ = 80 000 Pa · s (*τ*_1_ = 1*s*, *τ*_2_ = 20*s*, *α*_1_ = 0.1, *α*_2_ = 0.8). (H) Runtime of FEM‐veTFM, veTFM, and eTFM as a function of the number of time steps, evaluated on a grid of lateral size *L_x_* = *L_y_* = 50 µm (in the same order of magnitude as typical single‐cell dimensions) and resolution *n_x_* = *n_y_* = 41 (and *n_z_* = 12 for the FEM‐veTFM).

The GMX2 model consists of a purely elastic branch arranged in parallel with two Maxwell viscoelastic elements (Figure 2A). For simplicity, we assume viscoelastic effects influence only the deviatoric component of the stress tensor, although the formulation can also accommodate non‐deviatoric contributions if required (Section S3). Under our deviatoric assumptions, the behavior of GMX2 is fully determined by six independent parameters (Figure 2A): *E* —the elastic stiffness of the purely elastic branch; *E*_1_ and *E*_2_—the elastic stiffness of the two Maxwell elements; *η*_1_ and *η*_2_—the viscosity coefficients of the two Maxwell elements; and, *ν*—the Poisson's ratio of the elastic response of GMX2. To express GMX2 in terms of quantities that more directly capture its viscoelastic response under cell‐generated loading, we reparametrize the model in terms of the following independent parameters (Experimental Section)

(4) $$E_{0}=\sum_{i=1}^{2}E_{i}+E_{\infty }$$

(5) $$E_{\infty }=\frac{E}{1+\nu }\;$$

(6) $$\alpha_{i}=\frac{E_{i}}{E_{0}}\;\left(i=1,2\right)$$

(7) $$\tau_{i}=\frac{\eta_{i}}{E_{i}}\;\left(i=1,2\right)$$

The parameters *E*_∞_ and *E*_0_ represent, respectively, the long‐term (terminal) and short‐term (instantaneous) elastic stiffnesses. The parameters *α*_*i*_ indicate the fraction of stress dissipated by each Maxwell element, with their sum *α*_*t*_ = *α*_1_ + *α*_2_ distinguishing between solid‐like (*α*_*t*_ < 1) and fluid‐like (*α*_*t*_ = 1) viscoelastic materials. Finally, *τ*_*i*_ define the characteristic relaxation times of each Maxwell viscoelastic element.

### 2.4 Validation of veTFM for a Two‐Component Generalized Maxwell Model

Having formulated veTFM specifically for the GMX2 viscoelastic model in Fourier–Laplace space (Figure 1B, ii–iv, Equation (3)), we validated the feasibility of quantifying traction fields in real space (Figure 1B, v–vi) by specifically assessing the individual numerical inversion methods. While the inverse Fourier transform (2D‐IDFT) is well‐established [40, 41], validation of the inverse Laplace transform depends critically on substrate compressibility, characterized by the Poisson's ratio *ν*. For incompressible substrates (*ν* = 0.5), Laplace inversion is straightforward and analytically feasible (Experimental Section). However, for compressible substrates (ν≠0.5), inversion requires numerical methods due to the complexity arising from nonlinear expressions (Experimental Section and Section S3). Among available numerical inversion techniques [42, 43, 44], we adopted Talbot's method [44] due to its computational robustness and excellent performance (Experimental Section and Section S5). We further confirmed Talbot's inversion performance against an algebraic inversion that we developed for both GMX1 (one Maxwell branch only) and GMX2 (Section S3), but gets exponentially increasingly complex for GMXn with *n* > 2.

Having validated individual transformations, we next focused on the full veTFM algorithm. To maintain generality and challenge the algorithm's robustness, we selected demanding GMX2 parameters: characteristic relaxation times *τ*_1_ = 1*s* and *τ*_2_ = 20*s* (one order of magnitude apart) coupled to substantial partial dissipations *α*_1_ = 0.8 and *α*_2_ = 0.1. This choice yielded a total dissipation *α*_*t*_ = *α*_1_ + *α*_2_ = 0.9 < 1, indicative of significant viscoelasticity approaching fluid‐like behavior yet still within the solid‐like regime.

The validation of our veTFM solution addressed two distinct aspects: consistency, defined here as verifying whether the algorithm correctly implements theory in Equations (1), (2), and (3); and reliability, defined as verifying whether the algorithm's predictions match results obtained through independent methods (e.g., either analytical solutions or finite element models). To quantitatively assess both consistency and reliability of veTFM, we adopted two metrics measuring the similarity between any two given vector fields ***v*** (***r***, *t*) and ***w*** (***r***, *t*). The first metric was the L^2^‐norm (or Euclidean Distance) *ξ*, defined to measure local dissimilarity by capturing differences in both magnitude and direction between vectorial fields ***v*** and ***w*** at corresponding spatial and temporal locations—where ***v*** and ***w*** may represent any pair of displacement or traction fields computed by semi‐analytical, FEM, or analytical approaches. The second metric was defined to evaluate the relative maximum deviation across space and time, thus providing an estimate of the worst‐case error in assuming ***v*** and ***w*** are identical

(8) $$\xi \left(v,w\right)=\left|\left|v\left(r,t\right)-w\left(r,t\right)\right|\right|$$

(9) $$\xi_{\max}\left(v,w\right)=100\max_{r,t}\xi \left(v,w\right)/\max_{r,t}\left|\left|w\right|\right|$$

Given that experimental TFM data are typically acquired at discrete intervals Δ*t*, we tested algorithm performance across three realistic sampling regimes for the veTFM displacement input: (i) a high‐frequency sampling (Δ*t* < *τ*_1_ < *τ*_2_); (ii) an intermediate‐frequency sampling (*τ*_1_ < Δ*t* < *τ*_2_); and, (iii) a low‐frequency sampling (Δ*t* ≈ *τ*_2_ > *τ*_1_). Samplings with frequency significantly lower than these (Δ*t* > *τ*_2_) were not considered, as they yield negligible viscoelastic effects (Figure 2).

Due to the considerable variability in real cell‐generated substrate displacements, we tested the consistency and reliability of veTFM using two virtual displacement fields designed to challenge the algorithm. First, we employed a harmonic displacement field $u_{2D}^{H}=u_{0}\sin\left(2\pi \mathrm{ft}\right)e_{x}$, varying in time but uniform in space over the substrate's top surface (Figure 2D). Second, we used a quadrupolar displacement field $u_{2D}^{Q}$, constant in time but spatially varying with Gaussian profile in space (to ensure continuity and smoothness) in each of four regions over the substrate's top surface, and with vectors oriented centrally to mimic realistic cell‐generated displacement fields (Figure 2F). The maximum displacement value for both $u_{2D}^{H}$ and $u_{2D}^{Q}$ was set to *u*_0_ ≈ 0.2 µm (Figure 2D,F)—consistent with the typical submicron magnitude of cell‐induced displacements observed in TFM applications [1, 45, 46, 47].

To assess veTFM's consistency, defined as verifying correct theoretical implementation, we leveraged veTFM's capability to operate bidirectionally. Initially, we solved the inverse problem by computing the traction field ***T***_2D_ from the known displacement field ***u***_2D_ (Figure 2C, top chain). We then used ***T***_2D_ to solve the forward problem and determine the displacement $u_{2D}^{*}$(Figure 2C, bottom chain). The veTFM algorithm was deemed consistent if $\xi_{\max}\left(u_{2D},u_{2D}^{*}\right)$ remained below 5%—it is worth noting that $u_{2D}=u_{2D}^{*}$ represents an ideal case that may not be exactly achievable due to numerical errors arising from the Fourier or Laplace transforms and the time discretization. For harmonic displacement fields, simulations confirmed maximum errors $\xi_{\max}\left(u_{2D}^{H},u_{2D}^{*}\right)<5\%$ for all sampling regimes (Figure 2E, right). Similarly, quadrupolar displacement inputs produced maximum errors below 5% for high‐frequency sampling, and consistently negligible errors for intermediate and low‐frequency sampling (Figure 2G, right). Hence, veTFM demonstrated robust consistency by the defined criteria.

To explicitly assess veTFM reliability—defined as matching predictions from independent analytical or numerical methods—we compared the veTFM‐computed traction fields ***T***_2D_ with independent solutions. For harmonic displacement inputs with maximum magnitude *u*_0_ = 0.2 µm we computed the analytical solution with maximum magnitude $T_{2D}^{A}=200\;\mathrm{Pa}.$ Simulations showed maximum relative errors $\xi_{\max}\left(T_{2D}^{H},T_{2D}^{A}\right)<5\%$ under high‐frequency sampling conditions, while significantly higher errors (50%) arose at intermediate and low‐frequency sampling, reflecting insufficient temporal sampling of the displacement history used as input for viscoelastic traction quantification (Figure 2E, left, inset). For quadrupolar displacement inputs no direct analytical solution existed. We therefore developed a custom FEM‐veTFM method to obtain an independent solution with maximum magnitude $T_{2D}^{F}=400\;\mathrm{Pa}\;$(Experimental Section and Section S6). Because FEM‐veTFM numerically resolves Equations (1) and (2) over the full viscoelastic domain, it is computationally far more demanding than the semi‐analytical veTFM framework. Runtime benchmarking showed that veTFM remained computationally much closer to conventional eTFM than to FEM‐veTFM for cell‐sized problems, while being two to three orders of magnitude faster than FEM‐veTFM (Figure 2H). Our FEM‐veTFM reliably converged under high‐frequency and intermediate sampling only (Figure 2G, left, inset), providing a robust benchmark. In these sampling regimes, veTFM matched closely with FEM‐veTFM, showing maximum relative errors below 5%. However, at low‐frequency sampling intervals, FEM‐veTFM did not converge, preventing direct comparisons. Nonetheless, careful extrapolation from intermediate sampling intervals confirmed minimal deviation, thus affirming veTFM reliability under realistic conditions (Figure 2G, inset). In conclusion, validations under the defined criteria confirmed that our veTFM algorithm correctly implements theoretical formulations (consistency) and accurately predicts traction fields compared to independent analytical and numerical methods (reliability).

### 2.5 Experimental Application of veTFM: Reference‐Configuration and Pre‐Stress Effect

Two fundamental challenges arise when applying veTFM to experimental data in vitro, which do not manifest in simulations in silico. The first is the determination of a reference configuration required for computing in vitro substrate displacements. The second is the effect of substrate pre‐stress on cellular traction forces, which in viscoelastic materials depends on the entire history of deformations over time. Pre‐stress effect arises when, due to experimental constraints, the first measured displacements at the initial time point *t*_0_ do not coincide with the onset of cell adhesion at *t*_A_ (i.e., *t*_0_ ≥ *t*_A_), meaning that the initial displacements may already carry residual stress.

**FIGURE 3:**
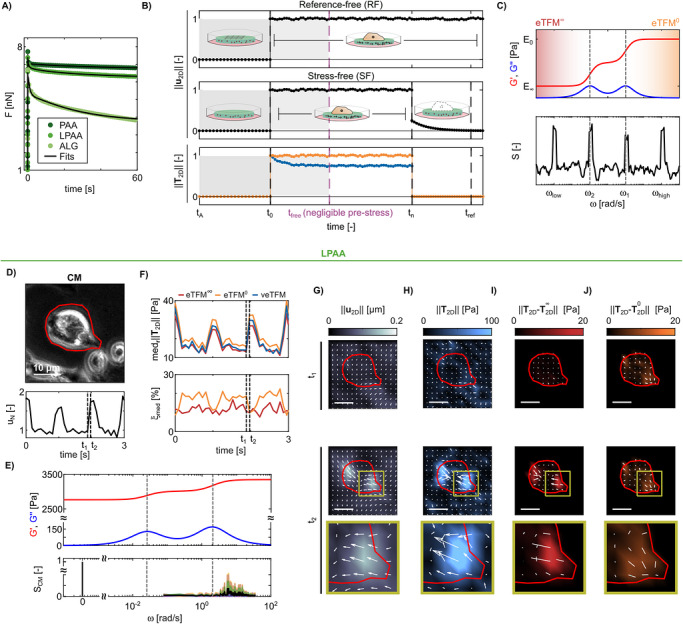
Experimental setup and application of veTFM to beating cardiomyocytes. (A) Stress‐relaxation response of the viscoelastic hydrogel substrates utilized for in vitro experiments (Methods): curves determined via atomic force microscopy (green lines) and in silico fitting (continuum black lines). (B) Schematic comparison of the stress‐free (SF) and reference‐free (RF) workflows in veTFM. Representative evolution of the average magnitudes of substrate displacements ***u***_2D_ (black dots) for the stress‐free and reference‐free workflows in veTFM, together with the corresponding cellular tractions ***T***_2D_ computed from these displacement fields using veTFM (blue dots) and eTFM^0^ (orange dots), from the time of cell adhesion *t*_A_ to the end of imaging at *t*_n_ in both RF and SF workflows, and additionally to stress‐free reference acquisition at *t*_ref_ in the SF workflow. Due to in vitro experimental demands or constraints, substrate displacements might only be recorded from *t*_0_ > *t*_A_; hence, the substrate may initially retain residual prestress. Time *t*_free_ denotes the time interval [*t*_0_, *t*_0_ + *t*_free_] required for this pre‐stress (and its effects on inferred tractions) to decay sufficiently, since computing tractions ***T***_2D_ at a generic timepoint *t_i_* requires, in principle, the displacement history ***u***_2D_ over the entire time interval [*t*_A_, *t_i_*]. (C) Illustrative viscoelastic traction components originated from a harmonic input displacement field $u_{2DH}$, such that, $T_{2D}^{H}=\varepsilon_{0}G^{\prime }\sin\left(\omega t\right)+\varepsilon_{0}G^{\prime \prime }\cos\left(\omega t\right)$. While *G*′ is in temporal phase with deformations and therefore elastic fields, *G*′′ introduces an out of phase temporal component typical of viscoelastic materials. *ω*_low_ denotes an example frequency for which the substrate behaves as a material with stiffness *E*_∞_, ω_high_ denotes an example frequency for which the substrate behaves as a material with stiffness *E*_0_, and *ω*_1_ *τ*_1_ = 1, *ω*_2_ *τ*_2_ = 1 the angular frequencies at which viscoelastic out of phase components, i.e., *G*′′, are maximized. (D) **Top**: representative phase‐contrast image of a beating cardiomyocyte (CM) cell used for veTFM. Red line represents the cell perimeter used for computation of median cellular tractions. Scale bars, 10 µm. **Bottom**: Normalized time evolution of the displacement magnitude *u*_N_ = *med*_***r***_|| ***u***_2D_||/*med*_***r***,*t*_|| ***u***_2D_||. (E) **Top**: Values of $G^{\prime }\left(\omega \right)$ and $G^{\prime \prime }\left(\omega \right)$ for LPAA. **Bottom**: CM power spectral density normalized to the zero‐frequency component obtained from time Fourier transformation of every pixel inside cell perimeter. Black lines indicate average over all pixels. For all graphs, vertical grey lines indicate *ω*_1_ and *ω*_2_. (F) **Top**: Median elastic and viscoelastic traction magnitudes. **Bottom**: Median relative error *ξ*_med_ against eTFM^0^ (orange) or eTFM^∞^ (red). The black dashed lines mark the consecutive timepoints *t*_1_ of diastole (non‐contracted phase) and *t*_2_ of systole (contracted phases) used to display the fields in (G–J). In (G–J), the lower panels show magnified views of the yellow boxed regions above. (G) Substrate‐displacement vector field obtained via image analysis, overlaid with a color‐coded scalar map of displacement magnitudes. (H) Cellular‐traction vector field obtained via veTFM, overlaid with a color‐coded scalar map of traction magnitudes. (I) Vector difference between traction fields from veTFM and eTFM^∞^, overlaid with a color‐coded scalar map of the difference traction magnitude. (J) Vector difference between traction fields from veTFM and eTFM^0^, overlaid with a color‐coded scalar map of the difference traction magnitude.

To address the reference configuration, we note that veTFM requires the substrate displacement field to be measured relative to a defined stress‐free configuration, irrespective of whether a stress‐free or a reference‐free workflow is used (Figure 3B). In reference‐free workflows, the reference configuration is known a priori from the predefined fiducial‐marker positions [21]. In stress‐free workflows [22], the reference configuration must instead be obtained experimentally after cell removal, by allowing the substrate to relax over a recovery interval Λ to a configuration with negligible residual stress. Upon ensuring that *Λ* exceeded the longest relaxation time of the material by one order of magnitude (Figure 3B), namely $\Lambda \gg \tau_{\max}=\max_{i=1,2}\tau_{i}\;,$ the stress‐free reference configuration is taken at a timepoint *t*_ref_ = *t*_n_ + *Λ* with *t*_n_ the last point of the imaging time‐lapse. It is worth noting that our in vitro experiments were conducted using a stress‐free workflow, for which the condition *Λ* ≫ *τ*_max_ was always satisfied.

To address the pre‐stress effect, we first considered that the current stress tensor **σ** (and tractions ***T***_2D_) at any time *t* > *t*_A_ depend theoretically on the entire history of substrate displacements over the interval [*t*_A_, *t*] (Experimental Section and Equation (S6)). This dependence applies irrespective of how the reference configuration is established (Figure 3B), such that both stress‐free and reference‐free workflows remain subject to the same history‐truncation problem when imaging does not capture the full displacement history from *t*_A_. Nevertheless, past displacements contribute progressively less to the current stress and tractions (Equation (15), Experimental Section, and Section S7), which allowed us to introduce a time offset *t*_free_ = 5*τ*_max_ representing the effective truncation point of the deformation history. Second, we considered that tracking displacement fields from the time point *t*_A_ may be experimentally impractical or even unfeasible (Figure 3B), and imaging may have to start from a certain timepoint *t*_0_ > *t*_A_. Analyses on substrates having a wide span of relaxation times (Section S7) showed that tractions ***T***_2D_ over the time interval [*t*_0_, *t*_0_ + *t*_free_] are not free of pre‐stress effects when prescinding from the deformation history over the interval [*t*_A_, *t*_0_] (Section S7). Therefore, veTFM tractions were computed starting from *t* ≥ *t*_0_ + *t*_free_ (Figure 3B) also for our in vitro case studies.

### 2.6 Experimental Application of veTFM: Three In Vitro Case Studies

We applied veTFM in vitro to quantify cellular traction forces on two viscoelastic hydrogel substrates: linear polyacrylamide (LPAA), the viscoelastic counterpart of conventional elastic polyacrylamide (PAA) widely used in TFM [27, 29]; and alginate (ALG), which offers broader tuneability of viscoelastic properties [48] (Figure 3A). We read out hydrogel displacements over time by embedding fluorescent beads (Figure 3B—top) and ensured cell adhesion to the substrate's top surface by functionalizing the gels for 2D TFM (Experimental Section). Local viscoelastic parameters at the cell‐substrate interface were obtained by performing AFM‐based rheology and by fitting a deviatoric GMX2 model to the force–distance curves (Figure 3A and the Experimental Section) [49]. LPAA hydrogels exhibited: terminal stiffness *E*_∞_ = 4.1 kPa; short and long relaxation times *τ*_1_ = 0.5s and *τ*_2_ = 34s, respectively; partial dissipations *α*_1_ = 0.07 and *α*_2_ = 0.05; and a moderate total dissipation *α*_*t*_ = *α*_1_ + *α*_2_ = 0.12 (Section S2). Alginate hydrogels showed: higher terminal stiffness *E*_∞_ = 36 kPa, similar relaxation times *τ*_1_ = 1s and *τ*_2_ = 35s; and, substantially larger total dissipation *α*_*t*_ = 0.56 with partial dissipations *α*_1_ = 0.26 and *α*_2_ = 0.3. These AFM fits support the response used in the GMX2 substrate model (Figure 3A). To suppress pre‐stress memory we initialized traction recovery at *t* ≥ *t*_0_ + *t*_free_ with *t*_free_ = 5*τ*_max_ (Figure 3B—bottom and Section S7)—namely *t*_free_ = 170s for the LPAA and *t*_free_ = 175s for the alginate.

Because viscoelastic tractions depend on the rate at which cells load and deform the hydrogel substrate (Experimental Section), we reasoned that each harmonic angular frequency *ω* (Section S8) in the time Fourier power‐density spectrum *S*(*ω*) of the hydrogel displacements (Figure 3C—bottom and Section S8) probes a particular point of *G*′(*ω*) and *G*′′(*ω*) (Figure 3C—top). We computed *S*(*ω*) and normalized it to the zero‐frequency (ZF) amplitude at *ω* = 0. We predicted that *G*′ ≈ *E*_∞_ and *G*′′ ≈ 0 (or *G*′′ ≪ *G*′) when most spectral density in *S*(*ω*) lies well below both rheological knees $\omega_{i}=\tau_{i}^{-1}$ (or *ω*_*i*_ *τ*_*i*_ = 1, with *i* = 1, 2) of the curve *G*′(*ω*) (Figure 3C—top), implying that the viscoelastic hydrogel would respond elastically to cell loading in this case—as if it were an elastic material with an effective stiffness *E*_eff_ ≈ *E*_∞_ (Section S8). In this low‐frequency limit, henceforth referred to as eTFM^∞^, we expected that average traction forces obtained by veTFM have the same order of magnitude as those produced by the elastic TFM (eTFM) algorithm on a substrate with stiffness *E* = *E*_∞_ (Figure 3C—top, left side corresponding to *ω*_low_). Conversely, if most spectral density in *S*(*ω*) lies well above both rheological knees $\omega_{i}=\tau_{i}^{-1}$ (or *ω*_*i*_ *τ*_*i*_ = 1, with *i* = 1, 2) of the curve *G*′(*ω*) (Figure 3C—top), then *G*′(*ω*) is close to *E*_0_ and *G*′′(*ω*) is modest (i.e., *G*′′ ≈ 0 or *G*′′ ≪ *G*′). In this high‐frequency limit, henceforth referred as eTFM^0^, we expected that average traction forces obtained by veTFM have the same order of magnitude as those produced by eTFM on a substrate with stiffness *E* = *E*_0_ (Figure 3C—top, right side corresponding to *ω*_high_). For spectral densities between the knees *ω*_1_ and *ω*_2_ (Figure 3C, top, central part), hydrogel responses can be viscoelastic (*G*′′ non‐negligible compared to *G*′) or elastic with an effective stiffness *E*_∞_ < *E*_eff_ < *E*_0_ (negligible *G*′′).

**FIGURE 4:**
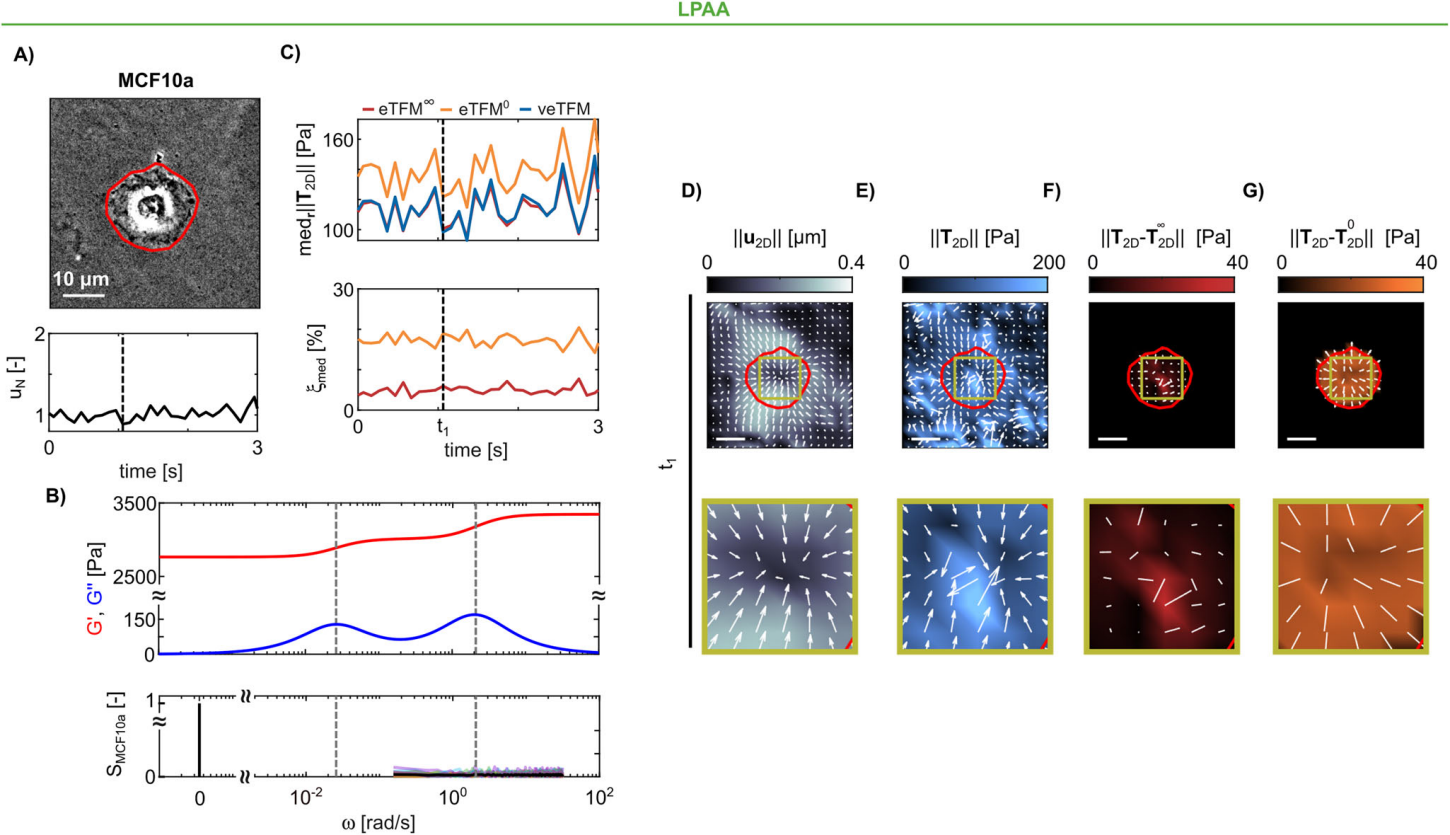
Experimental application of veTFM to epithelial cells. (A) **Top**: Representative phase‐contrast image of an MCF10A epithelial cell used for veTFM. Red line represents the cell perimeter used for computation of median cellular tractions. Scale bars, 10 µm. **Bottom**: Normalized time evolution of the displacement magnitude *u*_N_ = *med*_***r***_|| ***u***_2D_||/*med*_***r***,*t*_|| ***u***_2D_||. (B) **Top**: Values of $G^{\prime }\left(\omega \right)$ and $G^{\prime \prime }\left(\omega \right)$ for LPAA. **Bottom**: MCF10A power spectral density normalized to the zero‐frequency component obtained from time Fourier transformation of every pixel inside cell perimeter. Black lines indicate average over all pixels. For all graphs, vertical grey lines indicate *ω*_1_ and *ω*_2_. (C) **Top**: Median elastic and viscoelastic traction magnitudes. **Bottom**: Median relative error *ξ*_med_ against eTFM^0^ (orange) or eTFM^∞^ (red). The black dashed line marks the timepoint used to display the fields in (D‐G). In (D‐G), the lower panels show magnified views of the yellow boxed regions above. (D) Substrate‐displacement vector field obtained via image analysis, overlaid with a color‐coded scalar map of displacement magnitudes. (E) Cellular‐traction vector field obtained via veTFM, overlaid with a color‐coded scalar map of traction magnitudes. (F) Vector difference between traction fields from veTFM and eTFM^∞^, overlaid with a color‐coded scalar map of the difference traction magnitude. (G) Vector difference between traction fields from veTFM and eTFM^0^, overlaid with a color‐coded scalar map of the difference traction magnitude.

**FIGURE 5:**
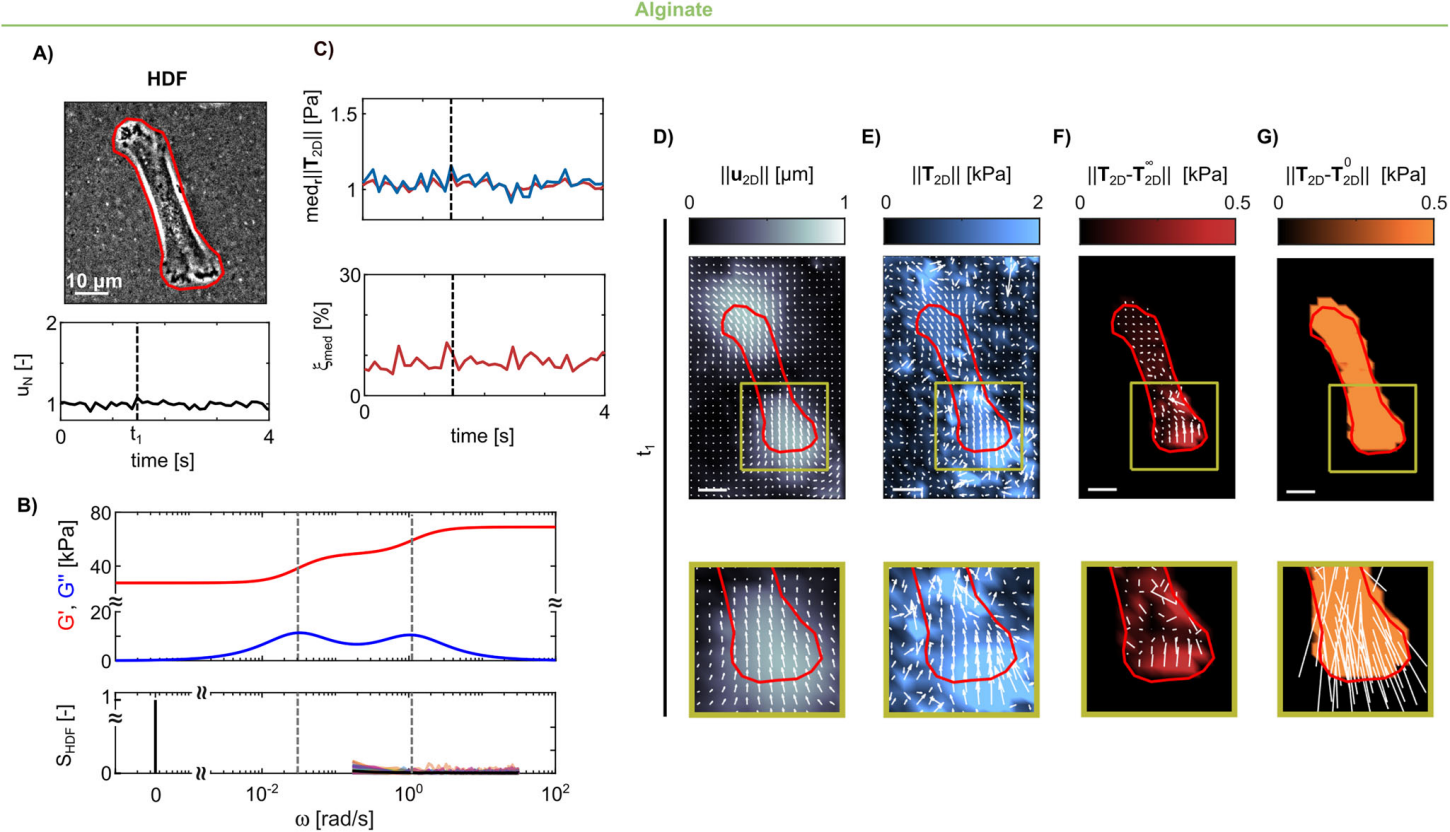
Experimental application of veTFM to fibroblasts. (A) **Top**: Representative phase‐contrast image of a human dermal fibroblast (HDF) used for veTFM. In red, cell perimeter used for computation of median cellular tractions. Scale bars, 10 µm. **Bottom**: Normalized time evolution of the displacement magnitude *u*_N_ = *med*_***r***_|| ***u***_2D_||/*med*_***r***,*t*_|| ***u***_2D_||. (B) **Top**: Values of $G^{\prime }\left(\omega \right)$ and $G^{\prime \prime }\left(\omega \right)$ for alginate. **Bottom**: HDF power spectral density normalized to the zero‐frequency component obtained from time Fourier transformation of every pixel inside cell perimeter. Black lines indicate average over all pixels. For all graphs, vertical gray lines indicate *ω*_1_ and *ω*_2_. (C) **Top**: Median elastic and viscoelastic traction magnitudes. **Bottom**: Median relative error *ξ*_med_ against eTFM^0^ (orange) or eTFM^∞^ (red). The black dashed line marks the timepoint used to display the fields in (D‐G). In (D‐G), the lower panels show magnified views of the yellow boxed regions above. (D) Substrate‐displacement vector field obtained via image analysis, overlaid with a color‐coded scalar map of displacement magnitudes. (E) Cellular‐traction vector field obtained via veTFM, overlaid with a color‐coded scalar map of traction magnitudes. (F) Vector difference between traction fields from veTFM and eTFM^∞^, overlaid with a color‐coded scalar map of the difference traction magnitude. (G) Vector difference between traction fields from veTFM and eTFM^0^, overlaid with a color‐coded scalar map of the difference traction magnitude.

To test these predictions, we examined three human cell types spanning distinct hydrogel loading regimes, namely: beating human cardiomyocytes (CM, Figure 3D) derived from human pluripotent stem cells (hPSC, Experimental Section) and seeded on LPAA; human mammary epithelial cells (MCF10A, Figure 4A) on LPAA; and, human dermal fibroblasts (HDF, Figure 5A) on alginate. In all three cases, the measured displacement fields remained within the linear viscoelastic regime of the corresponding hydrogels, supporting the use of a linear GMX constitutive description under the experimental conditions considered here (Figures S1, S7, S8, and S9). CMs were not used on alginate due to calcium sensitivity. Imaging intervals were short and comparable to ensure optimal veTFM performance across cell types (Δ*t* = 89 ms for CMs; and Δ*t* = 100 ms for MCF10A and HDFs; Experimental Section). The measured spectra *S*_CM_(*ω*), *S*_MCF10A_(*ω*), and *S*_HDF_(*ω*) corresponding to these three cell types (Figures 3E, 4B, and 5B—bottom) exhibited a dominant zero‐frequency (ZF) component (*ω* = 0) with a normalized density of 1, indicating a strong non‐oscillatory contribution. However, the three spectra differed at higher frequencies. *S*_MCF10A_(*ω*) and *S*_HDF_(*ω*) (Figures 4B and 5B—bottom) were mono‐modal with a predominant lobe with density ≈ 1 around ZF (*ω* ≈ 0) and content at higher *ω* spread roughly uniformly with lower and modest densities (≪ 1). Instead, *S*_CM_(*ω*) was bi‐modal (Figure 3E—bottom) with a secondary lobe at higher frequencies around *ω* ≈ 2π [rad/s], coinciding with the approximate systolic beating frequence of 1 Hz (Hertz) for CMs. By selecting these three cell types, we achieved to effectively capture a broad functional spectrum of dynamic cell–matrix interactions (Figures 3E, 4B, and 5B—bottom) across different viscoelastic substrates (Figures 3E, 4B, and 5B—top).

We then compared tractions computed via veTFM to the elastic algorithm (Boussinesq, finite thickness [2]) applied at stiffness *E* = *E*_0_ (eTFM^0^ limit) or *E* = *E*_∞_ (eTFM^∞^ limit). For each method we monitored the spatial median of traction magnitudes $\underset{r}{\mathrm{med}}\left||T_{2D}|\right|$ (Figures 3F, 4C, and 5C—top), while similarity differences between elastic and viscoelastic algorithms were quantified through $\xi_{\mathrm{med}}\left(T_{2D},T_{2D}^{\infty ,0}\right)=100\underset{r}{\mathrm{med}}\xi \left(T_{2D},T_{2D}^{\infty ,0}\right)/\underset{r}{\mathrm{med}}\left||T_{2D}^{\infty ,0}|\right|$ (Figures 3F, 4C, and 5C—bottom), with *ξ* defined by Equation (8) and ***T***_2D_, $T_{2D}^{\infty }$, and $T_{2D}^{0}$ the traction fields respectively obtained through veTFM, eTFM^∞^, and eTFM^0^. Numerical results are summarized in Table S2 and Section S10.

Average traction directions agreed closely across methods (mean angular deviations <10°, Figure S10). In contrast, average traction magnitudes varied in line with *S*(*ω*) for each cell type. For MCF10A on LPAA and HDF on alginate, whose spectra are ZF‐centred and largely non‐oscillatory (Figures 4B and 5B—bottom) veTFM magnitudes matched eTFM^∞^ on average (Figures 4C and 5C—top; Table S2). This is consistent with MCF10A and HDF cells predominantly loading the hydrogel at low *ω* (modal lobe of their respective spectra), where *G*′′(*ω*) is small and *G*′(*ω*) ≈ *E*_∞_, so the respective viscoelastic substrates behave (in terms of median traction magnitudes) as if elastic with an effective stiffness *E*_eff_ = *E*_∞_ (Figures 4F and 5F—top vs. Figures 4G and 5G—top; Section S10). In contrast, cardiomyocytes showed a rate‐dependent switching. During diastolic relaxation intervals (lower displacement magnitudes; Figure 3F—top), the ZF component dominates *S*_CM_(*ω*), and veTFM estimates shift toward eTFM^∞^ as for the non‐beating cell phenotypes (Figure 3H—top; Figure 3I—mid and bottom vs. Figure 3J—mid and bottom; Section S10), consistent with *G*′ ≈ *E*_∞_ and a negligible *G*′′ at lower frequencies for LPAA (Figure 3E—bottom, left part of the spectrum). During systolic beating (higher displacement magnitudes, Figure 3F—top), the spectral lobe of *S*_CM_ at higher *ω* becomes prominent (Figure 3F, top) and veTFM estimates shift toward eTFM^0^ (Figure 3I—top vs. Figure 3J—top; Section S10), consistent with *G*′ ≈ *E*_0_ and a negligible *G*′′ at higher frequencies for LPAA (Figure 3E—bottom, right part of the spectrum). As expected for systole, viscoelastic tractions point inward of the cell boundary (Figure 3H, red line) forming contractile dipoles (Figure 3H—mid; longer white arrows on blue shades). Notably, even when medians coincide over the whole cellular footprint at the interface with the hydrogel, local traction vectors can differ between veTFM and eTFM within the cell footprint (Figures 3I‐J, 4F‐G, and 5F‐G, bottom). These observations indicated that, for each of the tested cell types, there existed a viscous dissipation threshold beyond which eTFM fails to approximate viscoelastic tractions and veTFM becomes necessary. Thus, we set out to establish general criteria linking the choice between veTFM and its eTFM limits to the hydrogel's total dissipation and its timescale matching to cell loading.

### 2.7 Criteria for Selecting Viscoelastic vs. Elastic TFM Formulations

**FIGURE 6:**
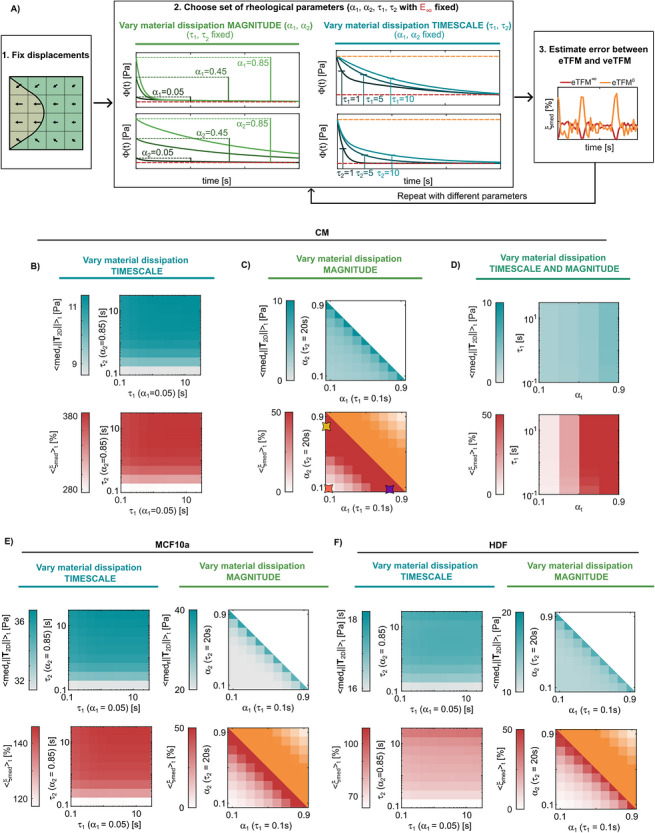
Global parameter‐space analysis of how substrate viscoelasticity affects tractions inferred for the three cell types. (A) Schematic of the computational framework developed to quantify differences in traction‐field inference between viscoelastic and elastic TFM algorithms. (1) Experimental displacement fields measured from cardiomyocytes (CM), MCF10A epithelial cells, and human dermal fibroblasts (HDF) were imposed on virtual viscoelastic substrates. (2) Substrates are assumed as having a range of GMX2 viscoelastic parameters *α*_1_,*α*_2_,*τ*_1_,*τ*_2_. (3) Traction fields are computed via veTFM and eTFM algorithms and differences between traction fields are quantitatively compared. (B–D) Application of the framework to CM displacements on LPAA. Mean over time of the median over space of the traction magnitude 〈*med*_***r***_|| ***T***_2D_||〉_*t*_ and mean over time of the traction magnitude median relative error 〈*ξ*_med_〉_*t*_ obtained from varying: (B) viscoelastic dissipation timescale; (C) viscoelastic dissipation magnitude; (D) both viscoelastic time and magnitude components. In C‐bottom, the red, yellow, and purple diamonds mark the three representative GMX2 cases analyzed in Figure 7. (E) Application of the framework to MCF10A displacements on LPAA. **Right**: varying viscoelastic dissipation magnitude. **Left**: varying viscoelastic dissipation timescales. **Bottom**: mean over time of the median relative error 〈*ξ*_med_〉_*t*_. **Top**: mean over time of the spatial median traction magnitude 〈*med_r_* ∣∣*T*_2D_∣∣〉_*t*_. (F) Application of the framework to HDF displacements on alginate. N.B. All simulations assume *E* = 700 Pa and *ν* = 0.4.

For users working on viscoelastic substrates, the practical question is whether a standard eTFM approximation remains sufficient and, if so, which elastic limit should be used between eTFM^0^ and TFM^∞^. To answer this within the GMX2 framework, we extended our analysis beyond the specific hydrogels tested experimentally, and computed traction fields on virtual GMX2 hydrogels by systematically varying their material parameters (Figure 6A2). The input displacements were kept fixed and drawn from the three in vitro cases previously analyzed (Figure 6A1), since those signals load the substrate over time with distinct mono‐ and bi‐modal spectra *S*(*ω*) (Figures 3E, 4B, and 5B—bottom). For each material‐parameter set, we quantified the difference between veTFM and the two eTFM limits through the *ξ*_med_ metrics previously defined (Figure 6A3).

We first considered fixed dissipation magnitudes (constant *α*_1_ and *α*_2_) while varying relaxation times *τ*_1_ and *τ*_2_ independently (Figure 6B and Figure S6). With a CM‐like displacement input, average viscoelastic traction magnitudes were insensitive to *τ*_1_ but responsive to *τ*_2_, the viscoelastic component associated with largest dissipation. When *τ*_2_ fell below 1s, the average magnitude increased by approximately 100%, whereas for $\tau_{2}≳1s$ the increase remained below 20% (Figure 6B, top). We then performed a second analysis in which we varied the total dissipation *α*_*t*_ = *α*_1_ + *α*_2_ together with *τ*_1_ (Figure 6D). Here, the average traction magnitude increased monotonically (by more than 50%) with *α*_*t*_ and remained nearly constant with *τ*_1_. Taken together, these two scenarios indicated that—contrary to common expectations in the literature attributing cell read‐outs to hydrogel relaxation times rather than total dissipation [50]—the distribution of relaxation times exerts less influence on traction magnitudes than the total dissipation. We therefore fixed the hydrogel's dissipation timescales *τ*_1_,*τ*_2_ and varied its dissipation magnitudes *α*_1_,*α*_2_ under the solid‐like constraint *α*_*t*_ < 1, with *α*_1_ and *α*_2_ ranging from 0.05 to 0.95 (Figure 6C). Simulations showed that viscoelastic traction magnitudes increased by more than 50% as either *α*_1_ or *α*_2_ rose (Figure 6C—top), and the discrepancy between veTFM and the eTFM limits grew as the hydrogel transitioned from solid‐like *α*_*t*_ < 1 toward the fluid‐like limit *α*_*t*_ ≈ 1 (Figure 6C, bottom), making veTFM indispensable because neither eTFM^0^ nor eTFM^∞^ can approximate the viscoelastic response with acceptable accuracy in that regime. It is worth noticing that the 50% threshold for the *ξ*_med_ error depended on the displacement profile (i.e., cell type): for CMs, the threshold occurred already at $\alpha_{t}≳0.6$ (Figure 6C—bottom); for MCF10As, only beyond $\alpha_{t}≳0.8$ (Figure 6E); for HDFs, close to the fluid‐like limit *α*_*t*_ ≈ 1 (Figure 6F).

To connect these global materials criteria with concrete cellular behaviors, we used a cardiomyocyte‐like input as a probe because its bi‐modal spectrum *S*_CM_(*ω*), with a dominant zero‐frequency component and a secondary high‐frequency lobe, could sample both low‐ and high‐rate cellular loading within one experiment. We then sought to disentangle and combine the effects of hydrogel dissipation magnitude (*α*_*t*_) and timescale matching (*ωτ*_*i*_) on the traction beating waveform and its amplitude by defining three GMX2 settings that isolate each factor and their interaction. The first setting was referred to as Low Dissipation (*α*_1_ = 0.05, *α*_2_ = 0.05, *α*_*t*_ = 0.1 ≪ 1, red diamond in Figure 6C—bottom) and represented a virtual hydrogel formulation that is weakly dissipative, strongly solid‐like and in which the GMX2 response is effectively elastic. The second setting was the Slow‐High Dissipation (*α*_1_ = 0.05, *α*_2_ = 0.85, *τ*_1_ = 0.1s, *τ*_2_ = 20s, *α*_*t*_ = 0.9 ≈ 1, yellow diamond in Figure 6C—bottom) and represented a virtual hydrogel formulation that was strongly fluid‐like and highly dissipative at the lower material's timescale, as $G^{\prime \prime }\left(\omega \right)$ presented a predominant dissipative term peaking in correspondence of the low‐frequency knee $\omega =\tau_{2}^{-1}\approx 0.05\;\left[\mathrm{rad}\;s^{-1}\right]$ of *G*′(*ω*) that does not catch nor match the CM systolic lobe of *S*_CM_(*ω*) at higher frequencies (Figure 3E—bottom). The third setting was the Fast–High Dissipation (*α*_1_ = 0.85, *α*_2_ = 0.05, *τ*_1_ = 0.1s, *τ*_2_ = 20s, *α*_*t*_ = 0.9 ≈ 1, purple diamond in Figure 6C—bottom) and represented a virtual hydrogel formulation that was also strongly fluid‐like but highly dissipative at the lower material's timescale, as $G^{\prime \prime }\left(\omega \right)$ presented a predominant dissipative term peaking in correspondence of the second‐knee $\omega =\tau_{1}^{-1}\approx 10\;\left[\mathrm{rad}\;s^{-1}\right]$ of *G*′(*ω*) that does catch and match the CM systolic lobe of *S*_CM_(*ω*) at higher frequencies (Figure 3E—bottom).

**FIGURE 7:**
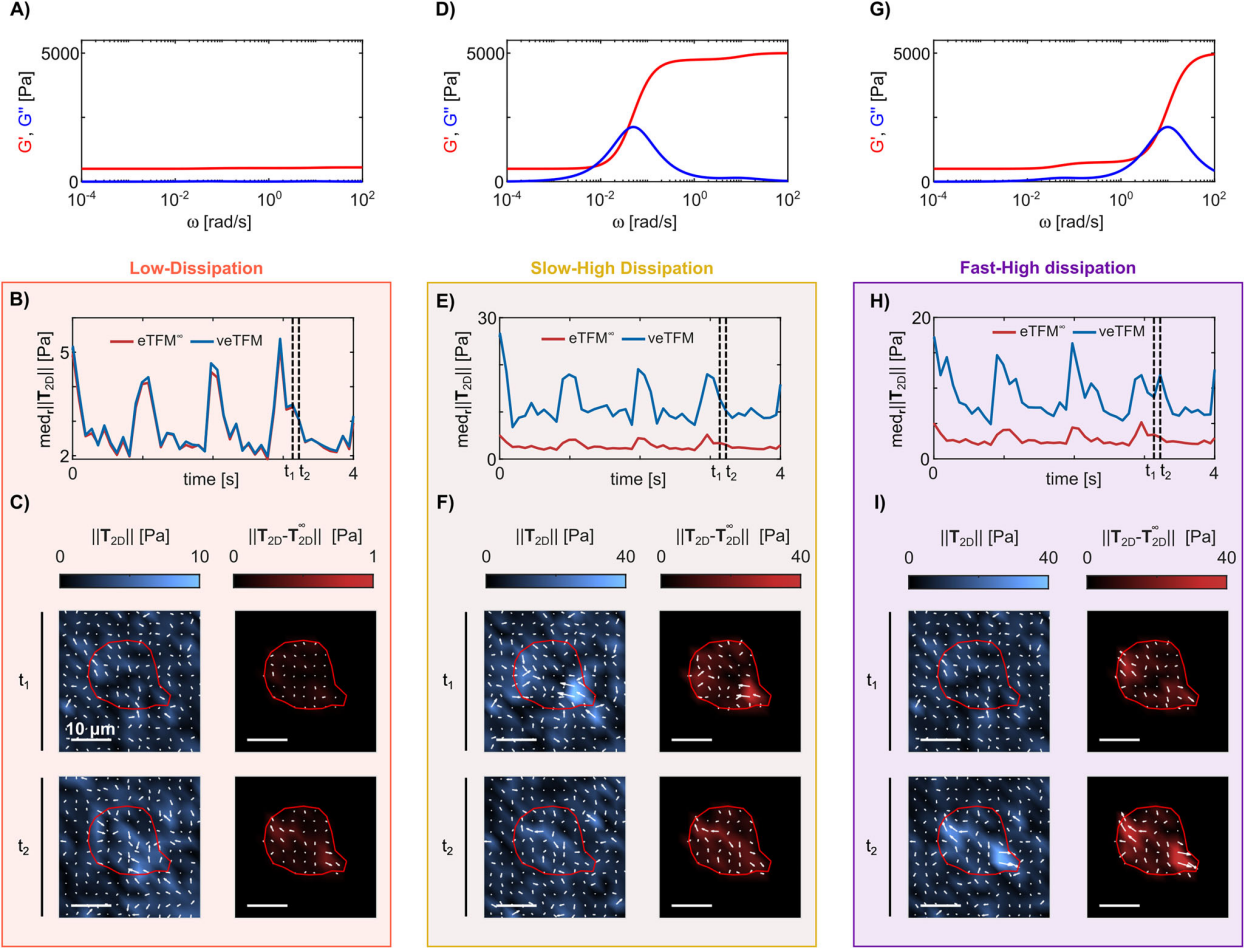
Representative cardiomyocyte traction waveforms and spatial fields for three GMX2 viscoelastic substrate regimes. (A–C) “Low Dissipation” case (corresponding to the red diamond in Figure 6C‐bottom): *α*_1_ = 0.05, *α*_2_ = 0.05, *τ*_1_ = 0.1*s*, *τ*_2_ = 20*s*; (D–F) “Slow‐High Dissipation” case (corresponding to the yellow diamond in Figure 6C‐bottom): *α*_1_ = 0.05, *α*_2_ = 0.85, *τ*_1_ = 0.1*s*, *τ*_2_ = 20*s*; and, (G–I) “Fast‐High Dissipation” case (corresponding to the purple diamond in Figure 6C‐bottom): *α*_1_ = 0.85, *α*_2_ = 0.05, *τ*_1_ = 0.1*s*, *τ*_2_ = 20*s*. (A, D, G) Storage and loss moduli, *G*′(*ω*) and *G*′′(*ω*), for the three selected GMX2 regimes. (B, E, H) Temporal evolution of the spatial median traction magnitude. Vertical dashed lines indicate the timepoints selected to display the fields below. (C, F, I) Local veTFM traction fields (blue) and corresponding traction‐difference fields relative to eTFM^∞^ at the selected timepoints during: top – *t*_1_, systole (contracting phase); bottom – *t*_2_, diastole (non‐contracting phase). N.B. All simulations assume *E* = 700 Pa and *ν* = 0.4.

Simulations showed that for Low‐Dissipation hydrogels, elastic and viscoelastic tractions overlapped almost exactly in average, confirming recovery of the elastic limit for negligible dissipation (Figure 7A,B,C). For Slow‐High Dissipation hydrogels, viscoelastic tractions diverged from eTFM^∞^ (Figure 7D–F), differently from the previous LPAA case and as expected given the higher *α*_*t*_ of this virtual hydrogel formulation. The diastole/systole pattern remained recognisable in the average viscoelastic tractions (Figure 7F), but both phases exhibited a uniform magnitude shift relative to the elastic baselines. For Fast–High Dissipation hydrogels, the traction magnitude shift persisted but the waveform was altered: secondary local maxima emerged shortly after systole (Figure 7H). Spatial maps at those time points showed outward‐pointing tractions at the cell boundary (Figure 7I; white vectors on blue shades)—an extensile dipolar signature—opposite to the contractile dipoles seen at systole on LPAA (Figure 3H –middle; white vectors on blue shades). Expressed as substrate reaction forces (opposite of cellular traction forces), the Fast–High Dissipative material lagged the cellular loading and transiently drove hydrogel compaction towards the CMs along the beating axis, rather than simply resisting it as in the LPAA case.

## 3 Discussion

Traction forces at the cell–ECM interface are a central mechanical readout of how cells engage, probe, and adapt to their environment [51, 52, 53, 54], and are therefore relevant not only to mechanobiology but also to the emerging mechanomedicine [30] and to regenerative engineering through cell‐instructive biomaterials [31]. Extracellular‐matrix viscoelasticity is not merely a background material property [5, 8, 9, 12, 13, 27], but an active regulator of cell behavior [32, 33], tissue organization [4], and disease‐relevant responses [5], making accurate traction quantification on viscoelastic substrates necessary for linking matrix mechanics to cell‐generated force transmission.

When the substrate is viscoelastic, however, the inferred traction field depends not only on the imposed substrate deformation but also on how the temporal content of cellular loading overlaps with substrate rheology. Studying viscoelastic traction forces is therefore necessary whenever substrate dissipation can alter the magnitude, temporal profile, or spatial organization of the computed forces.

A central practical outcome of this work is that the choice between eTFM^∞^, eTFM^0^, and veTFM can be made systematically from that overlap. In practical terms, eTFM^∞^ is appropriate when the cellular loading spectrum is concentrated well below the dominant rheological knees, eTFM^0^ when it is concentrated well above them, and veTFM otherwise or whenever total dissipation is high. The relevant predictor is therefore not cell type or displacement magnitude in isolation, but the full measured displacement spectrum relative to substrate rheology. Strict universal thresholds in terms of cell type, hydrogel rheology, or displacement magnitude cannot be given a priori for arbitrary cell‐hydrogel combinations, because none of these quantities alone is sufficient.

In this context, veTFM extends the elastic Boussinesq formulation of eTFM to viscoelastic substrates modeled as one‐ or two‐component Generalized Maxwell (GMX) materials while preserving the semi‐analytical structure that makes 2D eTFM fast and accessible. It also addresses viscoelastic‐specific experimental issues, including reference‐configuration handling and substrate pre‐stress. While runtime benchmarks show that veTFM is only modestly slower than eTFM, it provides higher traction accuracy when elastic approximations cease to be valid and remains far faster than FEM‐based viscoelastic quantification for cell‐sized problems.

Recent work has shown that traction responses can adapt rapidly to dynamic changes in substrate mechanics, whereas slower mechanosignaling processes evolve over longer timescales, underscoring the importance of resolving traction dynamics over time [55]. In this context, veTFM provides time‐resolved force readouts that can inform and test mechanosensing models, including molecular‐clutch frameworks extended to account for viscous substrate response [56], while also helping address the broader need for routine tools that quantify force‐regulated cell behavior over time in viscoelastic microenvironments. At the same time, like all TFM approaches, veTFM captures a key mechanical observable at the cell–ECM interface without directly resolving the full downstream intracellular or nuclear mechanotransduction state of the cell [57].

Across the three cell systems analyzed here, the effective mechanical response sampled by the cell depends on how the temporal content of the measured displacement field overlaps with substrate rheology. Rapid loading samples a stiffer, high‐frequency response; slow loading a softer, low‐frequency response; and intermediate loading produces traction distributions that diverge from any single elastic approximation. In this sense, slow loading tends towards the terminal elastic limit eTFM^∞^, whereas fast loading tends towards the instantaneous elastic limit eTFM^0^. This is most evident in the cardiomyocyte case, where the bi‐modal displacement spectrum indicates sampling of both low‐frequency and higher‐frequency regimes across the beating cycle. By contrast, for both MCF10A on LPAA and HDF on alginate, the measured displacement spectra are strongly centered at zero frequency and dominated by low‐frequency content, so that median traction magnitudes are well approximated by the low‐rate elastic limit eTFM^∞^. However, this agreement applies mainly to the median traction magnitude over the full cell footprint. Even when median magnitudes coincide, local traction vectors can still differ within the footprint, particularly for HDF on alginate because of the higher total dissipation of alginate. More generally, when loading lies predominantly within either elastic regime and dissipation is low, the corresponding elastic approximation can be sufficient on average, whereas veTFM remains preferable when local traction organization matters, when temporal waveform differences matter, or when dissipation is high.

Within the GMX2 framework analyzed here, the expanded parameter‐space analysis sharpens this practical picture. The range in which veTFM becomes necessary is cell dependent not because of cell identity itself, but because the experimentally measured displacement profiles span distinct spectral regimes. In the present cases, the 50% error threshold appears already at lower total dissipation for cardiomyocyte inputs, only beyond higher total dissipation for MCF10A, and only near the fluid‐like limit for HDF, consistent with the different spectral overlap of their displacement histories with the rheology. We therefore provide both a graphical guide (Figure 6) and a step‐by‐step adoption protocol (Section S1) for identifying, within the GMX2 framework analyzed here, when viscoelastic quantification is required and when an elastic approximation remains sufficient.

Among commonly used matrix classes, we envisage veTFM to be especially relevant for alginates [19], highly dissipating formulations of polyacrylamide [58], hyaluronic‐acid based hydrogels [59], and some protein‐based or supramolecular hydrogel formulations [60, 61]. It is also likely to be especially important for processes whose loading dynamics overlap with substrate relaxation times in the presence of substantial dissipation, including cardiomyocyte beating, cell migration, protrusion extension and retraction, and focal adhesion maturation [47, 54, 62, 63, 64].

Beyond the mechanosensing context discussed above, veTFM is also likely to be relevant wherever viscoelastic matrices are used but traction forces remain unquantified or are inferred using elastic approximations. This includes cell and tissue dynamics in viscoelastic microenvironments, where stress relaxation is increasingly used as a controlled material parameter [5, 13], regenerative biomaterials designed to instruct cell behavior through time‐dependent mechanics [5, 65, 66, 67], and viscoelastic matrices modeling disease in which traction may become a relevant quantitative mechanical phenotype [5, 68, 69, 70, 71]. More broadly, these settings span development, regeneration, ageing and disease, and include processes such as adhesion, proliferation, differentiation, and maturation that are shaped by time‐dependent matrix mechanics.

Two points are particularly important for interpreting these results. First, the main framework analyzed here is the viscoelastic analogue of conventional 2D elastic TFM and is therefore most appropriate when the traction force field is predominantly in‐plane. To examine the consequences of out‐of‐plane cellular loading more explicitly, we also provide a 2.5D viscoelastic formulation (Section S4). Importantly, because veTFM operates downstream of displacement computation, the quantification itself is agnostic to the imaging modality, displacement‐tracking workflow or reference‐definition strategy used upstream [2, 21, 22, 23, 24, 25, 26], provided that the required interfacial displacement field and substrate rheology are available.

Second, the present results establish a clear starting point for interpreting how substrate rheology shapes inferred tractions. Within the GMX2 framework analyzed here, total dissipation emerges as a stronger determinant of traction magnitude than any individual relaxation time. This conclusion is supported not only by the experimental comparisons, but also by the wider GMX2 parameter‐space exploration in which characteristic relaxation times and total dissipation were varied independently. At the same time, the present analysis is valuable precisely because it makes explicit which questions can now be addressed more systematically with veTFM. The current results isolate how inferred traction depends on substrate rheology within the GMX2 framework, while also showing that future studies can now test how these relationships extend across biological settings and to other viscoelastic material classes as these continue to emerge and become increasingly relevant in the field. In particular, other TFM substrate materials may belong to different rheological families and may exhibit broader or non‐GMX spectra, including materials better described by additional relaxation modes or by fractional or power‐law rheology [8, 38]. More generally, the Laplace‐inversion framework we presented here provides a basis that can in principle be extended to other rheological kernels, provided that the corresponding Laplace‐domain relaxation function is available, although this will require robust parameter identification and separate validation in each case.

Our quantitative analysis also establishes a practical criterion for handling displacement pre‐history under experimentally realistic sampling conditions in veTFM. Under the sampling interval used here, the adopted effective free‐history criterion keeps the relative error below 5% across the tested conditions, providing a useful balance between error control and practical feasibility. At the same time, the analysis makes clear that this criterion depends on temporal sampling as well as on substrate relaxation and dissipation, thereby providing a basis for adapting displacement pre‐history requirements to other experimental settings in veTFM (Section S1).

Taken together, veTFM establishes an adoptable extension of mainstream 2D TFM to viscoelastic substrates. Its value lies in turning viscoelastic traction quantification from an ad hoc or specialist exercise into a practical decision framework—one that identifies when elastic approximations remain valid, which elastic limit should be used when they do, and when full viscoelastic quantification is necessary. In this way, veTFM addresses a missing methodological link between the increasing use of viscoelastic matrices in mechanobiology, biomaterials, and regenerative engineering and the need for reliable traction‐based force readouts in those settings. It also enables users to move beyond displacement‐only analyses and ad hoc stiffness choices by recovering time‐resolved traction waveforms and local force organization on viscoelastic substrates, including in regimes where median traction magnitudes may still be well approximated by an elastic formulation. By coupling routine workflow compatibility with explicit scope conditions, veTFM is immediately usable in its present form while also providing a basis for future extensions to broader viscoelastic material classes.

Taken together, veTFM provides a practical extension of standard 2D TFM to viscoelastic substrates. It turns viscoelastic traction quantification from an ad hoc or specialist exercise into a usable framework for traction force quantification on viscoelastic substrates and for determining when elastic approximations remain valid, which elastic limit should be used when they do, and when full viscoelastic quantification is required. In this way, veTFM addresses a methodological gap between the increasing use of viscoelastic matrices in mechanobiology, biomaterials, and regenerative engineering and the need for reliable traction‐based force readouts in those settings. It also enables users to move beyond displacement‐only analyses and ad hoc stiffness choices by resolving time‐dependent traction dynamics and local force organization, including in regimes where median traction magnitudes remain well captured by an elastic formulation. By combining compatibility with existing TFM workflows with clearly defined conditions of applicability, veTFM is immediately usable in its present form while also providing a basis for future extensions to other viscoelastic material classes as these become increasingly relevant in the field.

## 4 Experimental Section

### 4.1 veTFM Implementation

Obtention of viscoelastic tractions as per veTFM can be described briefly as

1) For simplicity, we drop the subindex 2D, and define ***u*** (***r***, *t*) ≡ ***u***_2D_(***r***,*t*) = [*u_x_* (***r***, *h*, *t*), *u_y_* (***r***, *h*, *t*)] the obtained displacement field, first perform the 2D‐DFT on the *x* − *y* plane of each of the displacement field components

(10) $$F_{2}\left[u\left(r,t_{i}\right)\right]\left(k,t_{i}\right)\equiv \hat{u}\left(k,t_{i}\right)=\sum_{r}e^{-ik\cdot r}u\left(r,t_{i}\right)\;$$

with ***k*** the Fourier wavevector ***k*** = (*k_x_*,*k_y_*) and ***r*** the cartesian position vector ***r*** = (*x*, *y*).

2) Compute tractions in Fourier space. Define for a general function its Laplace transform $L\left[f(t)\right]\left(s\right)\equiv \tilde{f}\left(s\right)$, then for a given time *t_i_* and frequency ***k***, dropping dependencies for conciseness

(11) T^x=1kky2tanhkhL−1sΦ∼u∼^x−kxkytanhkhL−1sΦ∼u∼^y+ky2tanhkhL−12kx2L−1sΦ∼γ∼^u∼^x+2kxkyL−1sΦ∼γ∼^u∼^y

(12) T^y=1kkx2tanhkhL−1sΦ∼u∼^y−kxkytanhkhL−1sΦ∼u∼^x+kx2tanhkhL−12ky2L−1sΦ∼γ∼^u∼^y+2kxkyL−1sΦ∼γ∼^u∼^x

with $k=\sqrt{k_{x}^{2}+k_{y}^{2}}$, *h* the height of the substrate. The term corresponding to ***k*** = 0 needs to be computed separately as ${\hat{T}}_{x}\left(k=0,t_{i}\right)=L^{-1}\left[s\tilde{\Phi }{\hat{\tilde{u}}}_{x}\right]\left(k=0,t_{i}\right)/h$, ${\hat{T}}_{y}\left(k=0,t\right)=L^{-1}\left[s\tilde{\Phi }{\hat{\tilde{u}}}_{y}\right]\left(k=0,t_{i}\right)/h$ to avoid an apparent indetermination. Assuming *t_i_* > *t*_0_ = 0, linear interpolation of each of the components of the observed displacement fields leads to

(13) $$\begin{array}{cc} & L^{-1}\left[s\tilde{\Phi }\hat{\tilde{u}}\right]\;\left(k,t_{i}\right)\\ & \;=\hat{u}\left(k,0\right)\Phi \left(t_{i}\right)\\ & \;+\sum_{j=0}^{i-1}\left[\frac{\Delta {\hat{u}}_{j}\left(k\right)}{\Delta t_{j}}-\frac{\Delta {\hat{u}}_{j-1}\left(k\right)}{\Delta t_{j-1}}\right]L^{-1}\left[\tilde{\Phi }/s\right]\left(t_{i}-t_{j}\right) \end{array}$$

(14) $$\begin{array}{cc} & L^{-1}\left[s\tilde{\Phi }\hat{\tilde{\gamma }}\hat{\tilde{u}}\right]\left(k,t_{i}\right)\\ & \;=\hat{u}\left(k,0\right)L^{-1}\left[\tilde{\Phi }\hat{\tilde{\gamma }}\right]\left(k,t_{i}\right)\\ & \;+\sum_{j=0}^{i-1}\left[\frac{\Delta {\hat{u}}_{j}\left(k\right)}{\Delta t_{j}}-\frac{\Delta {\hat{u}}_{j-1}\left(k\right)}{\Delta t_{j-1}}\right]L^{-1}\left[\tilde{\Phi }\hat{\tilde{\gamma }}/s\right]\left(k,t_{i}-t_{j}\right) \end{array}$$

where Δ*t_j_* = *t*_*j* + 1_ − *t_j_*, $\Delta {\hat{u}}_{j}\left(k\right)=\hat{u}\left(k,t_{j+1}\right)-\hat{u}\left(k,t_{j}\right)$ with $\Delta {\hat{u}}_{-1}\equiv 0$. The solution up until this point remains general. For a GMX2

(15) $$\Phi \left(t\right)=\frac{E}{2\left(1+\nu \right)}+\sum_{i=1}^{2}\frac{E_{i}}{2}e^{-t\frac{E_{i}}{\eta_{i}}}\;$$

(16) $$L^{-1}\left[\tilde{\Phi }/s\right]\left(t\right)=\frac{E}{2\left(1+\nu \right)}t-\sum_{i=1}^{2}\frac{\eta_{i}}{2}\left[e^{-t\frac{E_{i}}{\eta_{i}}}-1\right]\;$$

Tractions in Fourier space can be computed after the remaining transformed terms $L^{-1}\left[\tilde{\Phi }\hat{\tilde{\gamma }}\right],$ $L^{-1}\left[\tilde{\Phi }\hat{\tilde{\gamma }}/s\right]$ are obtained:

a) If the substrate is incompressible, i.e., *ν* = 0.5, $\hat{\tilde{\gamma }}$ does not depend on *s* nor *t* (Section S3) and so analytical Laplace inversion is direct

(17) $$L^{-1}\left[\tilde{\Phi }\hat{\tilde{\gamma }}\right]\left(k,t\right)=\frac{\cosh^{2}\left(\mathrm{kh}\right)+k^{2}h^{2}}{\sinh\left(\mathrm{kh}\right)\cosh\left(\mathrm{kh}\right)+\mathrm{kh}}\Phi \left(t\right)$$

(18) $$L^{-1}\left[\tilde{\Phi }\hat{\tilde{\gamma }}/s\right]\left(k,t\right)=\frac{\cosh^{2}\left(\mathrm{kh}\right)+k^{2}h^{2}}{\sinh\left(\mathrm{kh}\right)\cosh\left(\mathrm{kh}\right)+\mathrm{kh}}L^{-1}\left[\tilde{\Phi }/s\right]\left(t\right)$$

b) If analytical inversion of the Laplace transform of a compressible substrate is considered

(19) $$L^{-1}\left[\tilde{\Phi }\hat{\tilde{\gamma }}\right]\left(k,t\right)=\Gamma_{e}+\sum_{i=1}^{6}\Gamma_{i}e^{-\kappa_{i}t}$$

(20) $$L^{-1}\left[\tilde{\Phi }\hat{\tilde{\gamma }}/s\right]\left(k,t\right)=\Gamma_{e}t-\sum_{i=1}^{6}\frac{\Gamma_{i}}{\kappa_{i}}\left[e^{-\kappa_{i}t}-1\right]\;$$

Parameters *Γ*_e_, *Γ*_*i*_, and *κ*_*i*_ for the deviatoric GMX2 and the deviatoric and non‐deviatoric GMX1 can be found in Section S3.

c) If numerical inversion of the Laplace transform of a compressible substrate is considered

(21) $$L^{-1}\left[\tilde{\Phi }\hat{\tilde{\gamma }}\right]\left(k,t\right)\approx \frac{\chi }{Mt}\sum_{m=0}^{M-1}Re\left[\delta_{m}\tilde{\Phi }\left(\frac{s_{m}}{t}\right)\hat{\tilde{\gamma }}\left(k,\frac{s_{m}}{t}\right)\right]$$

(22) $$L^{-1}\left[\tilde{\Phi }\hat{\tilde{\gamma }}/s\right]\left(k,t\right)\approx \frac{\chi }{Mt}\sum_{m=0}^{M-1}Re\left[\delta_{m}\frac{t}{s_{m}}\tilde{\Phi }\left(\frac{s_{m}}{t}\right)\hat{\tilde{\gamma }}\left(k,\frac{s_{m}}{t}\right)\right]\;$$

Which can be computed for any given $\tilde{\Phi }\left(s\right)$ and $\tilde{\Psi }\left(s\right)$ fulfilling the requirements for Talbot's numerical inversion (Section S3). For a deviatoric GMX2

(23) $$\tilde{\Phi }\left(s\right)=\frac{E}{2\left(1+\nu \right)}\frac{1}{s}+\frac{1}{2}\left[\frac{E_{1}}{s+E_{1}/\eta_{1}}+\frac{E_{2}}{s+E_{2}/\eta_{2}}\right]$$

(24) $$\tilde{\Psi }\left(s\right)=\frac{E\nu }{\left(1+\nu \right)\left(1-2\nu \right)}\frac{1}{s}-\frac{1}{3}\left[\frac{E_{1}}{s+E_{1}/\eta_{1}}+\frac{E_{2}}{s+E_{2}/\eta_{2}}\right]$$

(25) $$\begin{array}{cc} & \hat{\tilde{\gamma }}\left(k,s\right)\\ & \;=\frac{1}{\tilde{\Psi }+2\tilde{\Phi }}\frac{\cosh^{2}\left(\mathrm{kh}\right){\left(\tilde{\Psi }+2\tilde{\Phi }\right)}^{2}-{\tilde{\Phi }}^{2}\sinh^{2}\left(\mathrm{kh}\right)+k^{2}h^{2}{\left(\tilde{\Psi }+\tilde{\Phi }\right)}^{2}}{\sinh\left(\mathrm{kh}\right)\cosh\left(\mathrm{kh}\right)\left(\tilde{\Psi }+3\tilde{\Phi }\right)+\mathrm{kh}\left(\tilde{\Psi }+\tilde{\Phi }\right)} \end{array}$$

Required parameters for inversion *s_m_*, *δ*_*m*_, *χ* can be found in Section S3.

3. Obtain the traction field as function of time and space by performing an inverse 2D‐DFT as

(26) $$T\left(r,t\right)=\frac{1}{N}\sum_{k}e^{ik\cdot r}\hat{T}\left(k,t\right)\;$$

with *N* = *N_x_* *N_y_*, where *N_x_*, *N_y_* are, respectively, the number of points in *x* and *y* defining the discrete grid at which the displacement and traction fields are obtained.

### 4.2 Finite Element Modeling for Viscoelastic Traction Force Microscopy

The displacements inside the substrate and the resulting response tractions obtained by the 3D linear viscoelastic Finite Element model considered in this work result from solving

(27) $$K\left(t_{i}\right)u\left(t_{i}\right)=F^{r}\left(t_{i}\right)\;$$

with **K**(*t_i_*) the stiffness matrix and ***F** ^r^* (*t_i_*) the residual forces in the material, including tractions at its surface (Section S6). Computation of **K** and ***F*** ^***r***^ is obtained by considering a Generalized Maxwell model as well for which the stress is

(28) $$\sigma \left(t_{i}\right)=\sigma_{\infty }\left(t_{i}\right)+\sum_{k}^{N}\sigma_{k}\left(t_{i}\right)$$

(29) $$\sigma_{\infty }\left(t_{i}\right)=G_{e}:\varepsilon \left(t_{i}\right)\;$$

with ***G***_***e***_ the Hookean elasticity tensor, and where for a deviatoric Generalized Maxwell model, each of the viscoelastic components contributes as

(30) $$\sigma_{k}\left(t_{i}\right)=E_{k}\mathrm{dev}\left[\dot{\varepsilon }\left(t_{i}\right)\right]-E_{k}\frac{\sigma_{k}\left(t_{i}\right)}{\eta_{k}}$$

with $\mathrm{dev}\left[\dot{\varepsilon }\left(t_{i}\right)\right]=\dot{\varepsilon }\left(t_{i}\right)-I\mathrm{tr}\left[\dot{\varepsilon }\left(t_{i}\right)\right]/3$. Trilinear shape functions with 8 integration points are used for the finite elements, while the set of ODE's in time on **σ**_*k*_ are solved numerically by means of the Runge–Kutta 4 algorithm (Section S6). All results obtained using FEM as well as those obtained by veTFM in Figure 2 assume a substrate height of 2 µm to ensure comparability to the infinitely extending substrate assumption inherent to veTFM.

### 4.3 Preparation of Polyacrylamide, Linear Polyacrylamide, and Alginate Hydrogels for Viscoelastic TFM

Viscoelastic polyacrylamide hydrogels were prepared as previously described [72] with slight modifications (Table 1). First, functionalization of glass bottom wells for hydrogel attachment was obtained by preparing 357 µL of 3‐(Trimethoxysilyl)propyl methacrylate (Sigma‐Aldrich, 440159) dissolved in 4.286 mL of absolute ethanol for analysis (Merck, 1.00983) and 357 µL of acetic acid (Sigma‐Aldrich, A6283). 120 µL of the final solution were pipetted on top of 13 mm diameter glass wells of a 12‐well plate (MatTek). After 1 h, the well was rinsed with absolute ethanol (Technisolv, 83813.410) 3 times, dried with N_2_ and considered ready for hydrogel attachment. For gel preparation, linear polyacrylamide (LPAA) solutions were prepared by mixing, in the following order, MilliQ water (MQ), 40% acrylamide solution (Bio‐Rad, 1610140), 5% APS (Bio‐Rad, 1610700), and 1.6% TEMED (Sigma‐Aldrich, 102649815) in the appropriate amounts (Table 1). The LPAA mixture was incubated for 2 h at 37°C. Final viscoelastic and elastic hydrogel mixtures were then prepared by adding 2% bis‐acrylamide solution (Bio‐Rad, 1610142) and polymerization was initiated by adding TEMED and APS (Table 1). To prepare the viscoelastic linear polyacrylamide hydrogels, a certain volume of MQ was substituted by LPAA with respect to the elastic polyacrylamide hydrogel (Table 1). 11.5 µL of the final hydrogel mixtures were pipetted into each silanized glass well as fast as possible. Right after, the mixture was covered with a 13 mm diameter coverslip and gels were left to polymerize for at least 20 min. Afterwards, each well was covered in MQ water for approximately 1 h, after which the coverslip was removed with the help of needle and tweezers. To functionalize the hydrogel for cell attachment, 70 µL of Sulfo‐SANPAH (ThermoFisher, 22589) at a concentration of 1 mg/mL in MQ water was placed on top of each gel and illuminated for 5 min under a UV lamp (XX‐15, UVP) at 365 nm. Gels were then washed three times with sterile PBS and 60 µL of a 0.1 mg/mL collagen type IV (C5533, Sigma‐Aldrich) solution in sterile PBS was placed on top of each gel. After incubating gels overnight at 4°C and washing once with sterile PBS, hydrogels were considered ready for cell seeding. For alginate hydrogels, a glass bottom well plate identical to the one used for acrylamide hydrogels was silanized for alginate attachment by adding 120 µL of a 2% v/v APTES (Sigma‐Aldrich, 440140) solution in absolute ethanol for analysis (Merck, 1.00983). After 1 h, glass wells were rinsed 2 times with absolute ethanol (Technisolv, 83813.410) and 2 times with MQ water, then dried with N_2_. To prepare the hydrogel, very low molecular weight sodium alginate coupled with RGD peptides (Novatach/Sigma‐Aldrich, 4270101) was mixed with MQ water to obtain a 30 mg/mL alginate solution. 6 µL of the alginate solution was then placed on each glass well and covered with a 9 mm diameter glass coverslip. Then, each well was submerged in a 1 mL 100 mM CaCl_2_ (ACROS Organics, 349615000) solution and left to polymerize overnight at 37°C. Coverslips were then removed and alginate gels were considered ready for cell seeding. All hydrogels were functionalized for TFM by including dark red fluorescent spheres with a diameter of 0.2 µm (F8807, ThermoFisher). Beads were incorporated into hydrogel mixes to obtain a 1% v/v bead concentration before polymerization by thoroughly vortexing and re‐suspending.

**TABLE 1:** Components and volumes used for the preparation of hydrogels used in this work. All volumes in µL.

Reagent	Elastic PAA	Viscoelastic PAA	LPAA	Alginate
MilliQ (MQ)	287	125	317	400
LPAA	/	160	/	/
40% acrylamide	50	70	50	/
2% bis‐acrylamide	30	12	/	/
1.6% TEMED	25	25	25	/
5% APS	8	8	8	/
RGD alginic acid (mg)	/	/	/	12
Beads	4	4	/	4

### 4.4 Characterization of Viscoelastic Properties of Substrates by Atomic Force Microscopy

Viscoelastic properties of hydrogels were measured using the Cypher ES Environmental AFM. Hydrogels were polymerized between bind‐silane coated glass coverslips (*d* = 13 mm) and a hydrophobic rectangular microscopy glass slide. Silanized glass coverslips containing the gel were then glued to a metal puck (Electron Microscopy Services, 75010‐15) with conventional glue. At least 8 force‐distance curves per gel (*n* = 3 independent gels) were obtained using a spherical probe with spring constant *k* = 0.1 N/m and 6.44 µm tip diameter (CP‐qp‐CONT‐PM‐C‐5, sQube). All force–distance curves (*F* − *z*) were obtained using a piezo approach velocity of 5 µm/s and a relative force trigger of 8 nN. After reaching the force trigger, the piezo position (*z*) was kept fixed for at least 60s before retracting fully. For each force–distance curve, the contact point was initially estimated as the point with maximum deflection (*d*) variance within a 0.1 µm piezo position window [73]. From the initial estimated contact point, the true contact point (*z* = *z*_c_, *d* = *d*_off_) was then determined by fitting Hertzian contact models within a 0.05 µm interval in *z* by least squares minimization between the model and the experimental deflection (*F*/*k*).  From this, viscoelastic properties were obtained by the viscoelastic contact model [49]

(31) $$F\left(t\right)=\frac{8}{3\left(1-\nu \right)}\sqrt{R}\underset{0}{\int^{t}}\Phi \left(t-t^{\prime }\right)\frac{d(\delta {\left(t^{\prime }\right)}^{3/2})}{dt^{\prime }}dt^{\prime }$$

with *δ*(*t*) = (*z*(*t*) − *z_c_*) − (*d*(*t*) − *d*_off_) the indentation on the sample, *R* the radius of the probe, and *ν* the Poisson ratio of the sample. Rheological parameters defining a two‐component generalized Maxwell model (Equation (15)) were also obtained by minimization of least squares between the force obtained experimentally and via the contact model. In particular, MATLAB's constrained nonlinear multivariable solver (lsqnonlin) was used under constraints that all parameters defining *Φ*(*t*) remain non‐negative based on physical grounds.

### 4.5 Linear Viscoelastic Regime Rheological Measurements

Linear viscoelastic regimes for alginate and LPAA hydrogels were determined by performing a 0%–20% strain sweep using an Ares‐LS rheometer (TA instruments). In brief, the LPAA hydrogel mixture was placed inside a 25 mm diameter polydimethylsiloxane (PDMS) mold between a conventional plastic petri dish and a 32 mm diameter glass coverslip. Hydrogels polymerized after 1 h and were detached, placed in the rheometer in PBS and brought into contact with the geometry. Instead, alginate hydrogels were directly polymerized under the rheometer by adding 30 mg/mL of alginate solution in MilliQ (Table I) before bringing the mixture in contact with the geometry. Afterwards, the preparation was submerged in 100 mM CaCl_2_ and left to polymerize for 1 h. For both hydrogels, a sandblasted 25 mm parallel plate geometry was used.

### 4.6 Cell Culture

Human dermal fibroblasts (HDF, Lonza CC‐2511) were maintained in DMEM (Gibco, 41966) growth medium supplemented with 10% fetal bovine serum (Gibco, 10500064) and 1% Pen‐Strep (Gibco, 15140148) at 37°C, 5% CO_2_ and passaged every 3–4 days in a 1:5 split ratio. HDFs were dissociated using 0.05% Trypsin‐EDTA (Gibco, 25300‐054) and seeded on alginate gels at a density of 20k cells/mL per gel in the same cell medium used for maintenance. The hRAS inducible immortalized breast epithelial MCF10A cell line (a gift from Julian Downwards laboratory, University College London, London, UK) was maintained in DMEM/F‐12 without phenol red (ThermoFisher, 1103921), containing 5% charcoal‐stripped horse serum (ThermoFisher, 16050122), 20 ng/mL EGF (Peprotech, AF‐100‐15‐100UG), 0.5 mg/mL Hydrocortisone (Sigma‐Aldrich, H0888), 100 ng/mL Cholera toxin (Sigma‐Aldrich, C8052), 10 µg/mL Insulin (Sigma‐Aldrich, I6634), and 1% Pen‐Strep at 37°C with 5% CO_2_. Confluent cells were passaged every 2–3 days in a 1:5 split ratio. For cardiomyocytes (CMs) human pluripotent stem cells (hPSCs) from a previously described cell line (DRRAGN, kindly provided by Professor R. Passier, University of Twente), carrying a double reporter of mRubyll‐ACTN2 and GFP‐NKX2.5, were differentiated into CMs [46]. hPSCs were cultured on vitronectin‐coated (A14700, ThermoFisher) 6‐wells in Essential 8 medium (ThermoFisher, A1517001). Passaging of hPSCs was done by dissociation using 1x TrypLE in 1 mM EDTA in PBS (ThermoFisher, A1217701). Differentiation to CMs was done as described previously [74]. Briefly, hPSCs were seeded at a density of 85 K cells per cm^2^ on Matrigel‐coated 6‐well plates (Merck, CLS354234). Mesodermal differentiation was initiated through Wnt activation, by treating cells with 3 µM CHIR99021 (Axon Medchem, CT99021) in RPMI‐1640 medium (ThermoFisher, 11835063) with B‐27 supplement minus insulin (ThermoFisher, A1895601). After 3 days, Wnt was inactivated by adding XAV939 (Bio‐Techne, 3748/10) in the RPMI/B‐27 medium, with a Matrigel overlay (1:200) to promote cell adhesion. On day 5 of differentiation, cell cultures were refreshed with RPMI/B‐27 medium. On day 7 of differentiation, medium was changed to RPMI‐1640 with B‐27 supplement (ThermoFisher, 11530536). After this point, CMs started beating. On day 10 of differentiation, purification was started by addition of RPMI‐1640 without glucose (ThermoFisher, 11879020) supplemented with B‐27. After 2 days of purification, cells were ready to use for experiments. Differentiated CMs were dissociated using 10x TrypLE (ThermoFisher, A1217701) and replated on the coated PAA gels at a density of 80k cells per gel in RPMI/B27.

### 4.7 Time‐Lapse Microscopy and Displacement Field Acquisition

Cells and fluorescent beads were imaged using a Nikon Eclipse Ti2 microscope with a 20x objective (Plan Apo λ 20x Ph2 DM, 0.75 NA). Cell and bead imaging happened the day after cell seeding for CMs, and 10 h after for MCF10A and HDF, at environmental conditions of 37°C, 5% CO_2_, and 90% humidity for all cases. A single phase contrast image was taken at start of imaging, and afterward beads were imaged for 5 min every 100 ms for MCF10A and HDF, and for 5 min every 89 ms for CMs. Displacement fields on the cell‐substrate interface were obtained by performing particle image velocimetry [34] between experimental time‐lapse images of fluorescent beads capturing cell generated deformations and the stress‐free reference configuration, obtained 5 min after removal of cells by adding 10% SDS (05030‐500ML‐F, Sigma‐Aldrich) solution into each well. For PIV, a full image corresponding to a region of interest was divided into smaller interrogation windows of 32 by 32 pixels in size with 50% step overlap.

## Author Contributions

A.V.R. developed veTFM; A.V.R. validated veTFM with assistance from J.J.M.; A.V.R., J.J.M. and V.C. designed in silico experiments; A.V.R, P.v.d.B., D.C.A.D., and V.C. designed in vitro experiments; M.A.G.O., G.C. and M.S.S. developed LPAA protocols; A.V.R. and S.P. developed alginate protocols; A.V.R., D.C.A.D., P.v.d.B., M.A.G.O., G.C. and M.S.S. adapted hydrogel experimental protocols for veTFM; P.v.d.B. performed MCF10A experiments; D.C.A.D. performed cardiomyocyte experiments; A.V.R. performed human dermal fibroblast experiments; A.V.R., D.C.A.D. and P.v.d.B. produced in vitro data; A.V.R. analysed in vitro data, produced and analysed in silico data; A.V.R., P.v.d.B., B.G. and D.C.A.D. measured hydrogel viscoelastic rheology; A.V.R, D.C.A.D., P.v.d.B., J.J.M. and V.C. wrote the manuscript; all authors discussed and commented on the manuscript; V.C. secured funding, conceived and supervised the entire project.

## Code Availability

The MATLAB implementation of the veTFM algorithm is available at https://github.com/SMorphs/veTFM.

The repository includes the executable core code, the required input data and a user interface script to adjust relevant parameters and run the method.

## Conflicts of Interest

The authors declare no conflicts of interest.

## Supporting information




**Supporting File**: advs75490‐sup‐0001‐SuppMat.pdf.

## Data Availability

The synthetic datasets used to validate the computational method and support the biological findings are available at the following GitHub repository: https://github.com/SMorphs/veTFM.
