# Supplementary material for: Traction Force Microscopy for Viscoelastic Substrates: A Semi‐Analytical Method
Source: Adv Sci (Weinh). 2026 May 28;13(42):e22252. doi: 10.1002/advs.202522252 (PMC13335583; doi:10.1002/advs.202522252)
Supplement: Supplementary file 1 — Supporting File: advs75490‐sup‐0001‐SuppMat.pdf. [file ADVS-13-e22252-s001.pdf]

Supplementary Information for Traction Force Microscopy for viscoelastic substrates: a semi-analytical algorithm

A. Villacrosa-Ribas, D.C.A. Duffhues, P.v.d. Bersselaar, S. Pragnere, B.G.W. Groenen, M.A.G. Oliva, G. Ciccone, M. Salmeron-Sanchez, C.V.C. Bouten, J.J. Muñoz, V. Conte

Contents

S1: Practical implementation of veTFM **2**

S2: Rheological modelling and characterization of polyacrylamide and alginate hydrogels **3**

S3: Extended derivation of veTFM and Laplace transform inversion **4**

S4: Computation of out of plane tractions (T_z) using veTFM **10**

S5: Extended validation of veTFM **12**

S6: Finite Element Modelling of viscoelastic Traction Force Microscopy **13**

S7: Assessment of pre-stress effects in viscoelastic substrates **15**

S8: Elastic limits in harmonic displacement fields **17**

S9: Strains exerted by cardiomyocytes, MCF10a cells and human dermal fibroblasts **19**

S10: Summary of experimental viscoelastic tractions **20**

S1: Practical implementation of veTFM

1. **Characterize the material properties of the substrate** to obtain the viscoelastic parameters. For the GMX2 model, these are $E, E_1, E_2, \eta_1, \eta_2$:
   - If performing dynamic mechanical analysis measurements, fit the storage and loss moduli (Eqs. 3, 4).
   - If performing bulk stress relaxation measurements, fit the stress relaxation function (Eq. 1).
   - If performing AFM-based stress relaxation, fit the viscoelastic Hertzian contact model (Eq. 5).
2. **Compute the relevant viscoelastic parameters** for analysis and selection of the traction reconstruction algorithm:
   - Determine the relaxation times (i.e. the viscoelastic time components) as $\tau_k = \eta_k/E_k$ ($k = 1$ for GMX1 and $k = 1, 2$ for GMX2).
   - Compute the terminal and instantaneous stiffness values as $E_\infty = E/(1 + \nu)$ and $E_0 = E_\infty + \sum_k E_k$ respectively.
   - Evaluate the dissipations (i.e. viscoelastic magnitude components) as $\alpha_k = E_k/E_0$ and $\alpha_t = \sum_k \alpha_k$.
3. **Obtain the three-dimensional displacement field generated by the cell** at the cell-substrate interface `ux(x,y,t)`, `uy(x,y,t)`:
   - If a reference-free approach is applied, the displacement field arrays are obtained directly.
   - If a stress-free reference scheme is used:
     (a) Remove the cells from the substrate to obtain a stress-free configuration.
     (b) Acquire the stress-free reference image at a time at least one order of magnitude larger than the maximum relaxation time of the material after cell detachment.
     (c) Determine the substrate displacements by comparing the stress-free reference and live images, e.g using an image-processing tool such as Particle Image Velocimetry (PIV) or Digital Volume Correlation (DVC) (PIVlab for MATLAB in our implementation).
4. **Choose the appropriate traction reconstruction algorithm** (see Section S8). These guidelines are general and may not apply to all systems; therefore, use of veTFM is recommended if possible. However, if an elastic approach is preferred:
   (a) Compute the temporal Fourier spectrum, $S(\omega)$, of the displacement field generated by the cell for each pixel (`fft` over time in MATLAB in our implementation).
   (b) Select the corresponding algorithm depending on the obtained spectra and the measurement of interest.
      i. If $\alpha_t \geq 0.8$ or if local/time dependent tractions magnitudes are crucial, select veTFM (e.g. local tractions of HDF on alginate in this work).
      ii. If $\alpha_t < 0.8$ and only average traction magnitudes are relevant, let ω_j be the dominant frequency components of $S(\omega)$, then:
         - If $\omega_j \tau_k \approx 1$ or if both $\omega_j \tau_k >> 1$ and $\omega_j \tau_k << 1$ occur for any combinations of k, j, select veTFM (e.g. cardiomyocytes on LPAA in this work).
         - If $\omega_j \tau_k >> 1$ for all k, j, use the elastic TFM algorithm with effective stiffness $E_{eff} = E_0$.
         - If $\omega_j \tau_k << 1$ for all k, j use the elastic TFM algorithm with effective stiffness $E_{eff} = E_\infty$ (e.g. MCF10a on LPAA or HDF on alginate in this work).

5. **Compute the traction fields using veTFM, if required**. In our MATLAB implementation available at the public repository `https://github.com/SMorphs/veTFM`, call `veTFM_2D`. Instructions on its use can also be found in the public repository. The numerical and computational details of the veTFM implementation are described in the Experimental Section of the manuscript.

6. **Correct for pre-stress, if necessary.** If substrate displacements are expected to be non-zero prior to the start of imaging (t_0), record displacements from t_0 but consider tractions to be reliable only for times $t > t_0 + 5\max_k \tau_k$ to avoid pre-stress effects. This correction is necessary regardless of the method used to obtain displacements (stress-free or reference-free).

S2: Rheological modelling and characterization of polyacrylamide and alginate hydrogels

Rheological parameters defining $\Phi(t)$ can be obtained through different rheological experimental tests.

- For bulk rheology, if a stress relaxation test is performed, i.e, constant sudden strain $\boldsymbol{\varepsilon}(t) = \varepsilon_0 H(t)$, for a second order Generalized Maxwell Model (GMX2):

$$\sigma(t) = 2\Phi(t)\varepsilon_0 = \left(\frac{E}{1+\nu} + E_1 e^{-t/\tau_1} + E_2 e^{-t/\tau_2}\right)\varepsilon_0 \tag{1}$$

  with ν Poisson's ratio.

- For dynamic mechanical analysis (bulk), an oscillating strain can be applied to the system such that $\boldsymbol{\varepsilon}(t) = \varepsilon_0 \sin(\omega t)$ ($\omega = constant$), under which:

$$\sigma(t) = G'(\omega)\sin(\omega t)\varepsilon_0 + G''(\omega)\cos(\omega t)\varepsilon_0 \tag{2}$$

  where for a GMX2:

$$G'(\omega) = E_1 \frac{\omega^2\tau_1^2}{1+\omega^2\tau_1^2} + E_2 \frac{\omega^2\tau_2^2}{1+\omega^2\tau_2^2} + \frac{E}{1+\nu} \tag{3}$$

$$G''(\omega) = E_1 \frac{\omega\tau_1}{1+\omega^2\tau_1^2} + E_2 \frac{\omega\tau_2}{1+\omega^2\tau_2^2} \tag{4}$$

  which can be fitted similarly to obtain the parameters defining the GMX2, i.e., E, E_1, E_2 and η_1, η_2 ($\tau_k = \eta_k/E_k$).

- For AFM-based rheology, for a spherical cantilever with radius R:

$$F(t) = \frac{8}{3(1-\nu)}\sqrt{R}\int_0^t \Phi(t-t')\frac{d(\delta(t')^{3/2})}{dt'}dt' \tag{5}$$

  with $\delta(t) = z(t) - z_c - (d(t) - d_{off})$ the indentation on the substrate, d, z the measured deflection and piezo position of the cantilever respectively, and d_{off}, z_c the deflection and piezo position at contact between the probe and the substrate. This formulation assumes that the viscoelastic components affect only the deviatoric part of the stress tensor (Eq. 16, 17).

  In this work, viscoelastic properties of hydrogels were obtained using AFM, resulting in:

Material	E [kPa]	ν [-]	E_1 [kPa]	η_1 [kPa·s]	E_2 [kPa]	η_2 [kPa·s]
PAA	5.25 ± 0.2	0.48	0.37 ± 0.03	0.09 ± 0.01	0.22 ± 0.03	10.60 ± 1.74
LPAA	4.09 ± 0.12	0.48	0.33 ± 0.02	0.16 ± 0.03	0.25 ± 0.03	9.65 ± 2.95
Alginate	36.03 ± 3.98	0.31	19.81 ± 1.96	17.99 ± 1.52	21.71 ± 1.85	708.24 ± 52.64

Table 1: Viscoelastic properties of hydrogels. Average GMX2 parameters obtained by fitting AFM force-distance curves from equation 5 (± indicate standard error). At least 20 points for each of three separate hydrogels were obtained for alginate and LPAA and at least 8 points for PAA. Poisson ratio's ν for each type of hydrogel were taken from literature [1, 2].

Parallelly, bulk rheology tests were used to obtain the linear viscoelastic regime of the hydrogels considered in veTFM (Fig. S1).

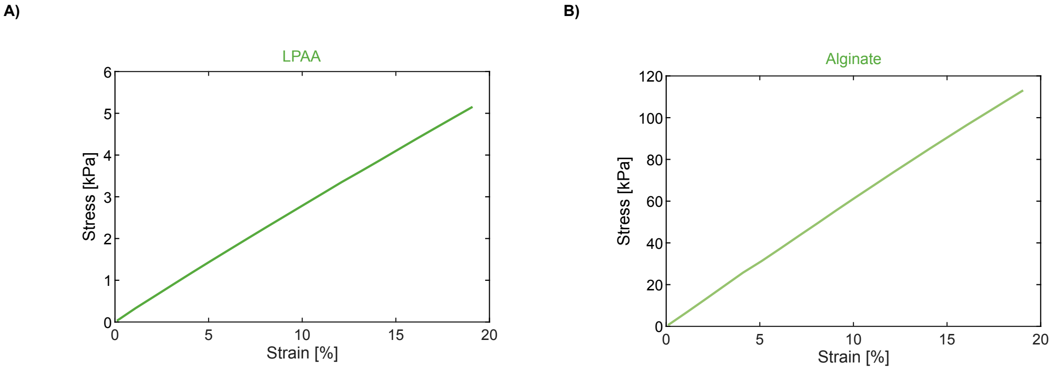

Fig. S1: Linear constitutive regime of alginate and linear polyacrylamide hydrogels A, B) Stress as a function of strain for LPAA (A) and alginate hydrogels (B) as obtained by bulk rheology.

S3: Extended derivation of veTFM and Laplace transform inversion

In this work, linear viscoelasticity is formulated by Boltzmann's superposition principle. Assuming $t_0 = 0$ for simplicity:

$$\boldsymbol{\sigma}(\boldsymbol{r}, z, t) = \int_0^t \boldsymbol{\mathcal{G}}(t - t') : \dot{\boldsymbol{\varepsilon}}(\boldsymbol{r}, z, t')dt' \tag{6}$$

where $\boldsymbol{r} = (x, y)$ and $\dot{\boldsymbol{\varepsilon}}(\boldsymbol{r}, z, t) = \partial/\partial t[\boldsymbol{\nabla u}(\boldsymbol{r}, z, t) + (\boldsymbol{\nabla u}(\boldsymbol{r}, z, t))^T]/2$ the infinitesimal strain rate tensor. $\boldsymbol{\mathcal{G}}$ is the general linear, isotropic, homogeneous viscoelasticity tensor, defined with components:

$$\mathcal{G}_{abcd} \equiv \Psi(t)\delta_{ab}\delta_{cd} + 2\Phi(t)\delta_{ac}\delta_{bd} \tag{7}$$

The complete system of equations defining mechanical equilibrium is then:

$$\boldsymbol{\nabla} \cdot \left(\int_0^t \boldsymbol{\mathcal{G}}(t - t') : \dot{\boldsymbol{\varepsilon}}(\boldsymbol{r}, z, t')dt' \right) = 0 \tag{8}$$

where $\boldsymbol{\nabla} = (\partial_x, \partial_y, \partial_z)$. Let $\boldsymbol{k} = (k_x, k_y)$ be the $x - y$ associated Fourier wave-vector, s the time associated Laplace domain variable, then:

$$\mathcal{L}[f(\boldsymbol{r}, z, t)] \equiv \tilde{f}(\boldsymbol{r}, z, s) \tag{9}$$

$$\mathcal{F}_2[f(\boldsymbol{r}, z, t)] \equiv \hat{f}(\boldsymbol{k}, z, t) \tag{10}$$

$$\mathcal{L}[\mathcal{F}_2[f(\boldsymbol{r},z,t)]] \equiv \hat{\tilde{f}}(\boldsymbol{k},z,s) \tag{11}$$

The Laplace transform of Eq. 6 results in $\tilde{\boldsymbol{\sigma}}(\boldsymbol{r},z,s) = s\tilde{\boldsymbol{\mathcal{G}}}(s)\tilde{\varepsilon}(\boldsymbol{r},z,s)$, and mechanical equilibrium is, under no body load and after taking the 2D Fourier transform:

$$\left[\tilde{\Psi}(s)+\tilde{\Phi}(s)\right]\boldsymbol{k}_z(\boldsymbol{k}_z\cdot\hat{\tilde{\boldsymbol{u}}}(\boldsymbol{k},z,s)) + \tilde{\Phi}(s)(\boldsymbol{k}_z\cdot\boldsymbol{k}_z)\hat{\tilde{\boldsymbol{u}}}(\boldsymbol{k},z,s) = 0$$

where $\boldsymbol{k}_z = (k_x, k_y, \partial_z)$. In this derivation $\tilde{\Psi}(s)$ and $\tilde{\Phi}(s)$ are not restricted to the generalized Maxwell model and remain general. By performing both transforms, the viscoelastic mechanical equilibrium problem is comparable to the elastic case [3]. The system of ordinary differential equations in z to be solved is:

$$\begin{aligned}
&(-k^2+\partial_z^2)\hat{\tilde{u}}_x(\boldsymbol{k},z,s) + i\frac{\tilde{\Psi}(s)+\tilde{\Phi}(s)}{\tilde{\Phi}(s)}k_x(ik_x\hat{\tilde{u}}_x(\boldsymbol{k},z,s)+ik_y\hat{\tilde{u}}_y(\boldsymbol{k},z,s)+\partial_z\hat{\tilde{u}}_z(\boldsymbol{k},z,s)) = 0\\
&(-k^2+\partial_z^2)\hat{\tilde{u}}_y(\boldsymbol{k},z,s) + i\frac{\tilde{\Psi}(s)+\tilde{\Phi}(s)}{\tilde{\Phi}(s)}k_y(ik_x\hat{\tilde{u}}_x(\boldsymbol{k},z,s)+ik_y\hat{\tilde{u}}_y(\boldsymbol{k},z,s)+\partial_z\hat{\tilde{u}}_z(\boldsymbol{k},z,s)) = 0\\
&(-k^2+\partial_z^2)\hat{\tilde{u}}_z(\boldsymbol{k},z,s) + \frac{\tilde{\Psi}(s)+\tilde{\Phi}(s)}{\tilde{\Phi}(s)}\partial_z(ik_x\hat{\tilde{u}}_x(\boldsymbol{k},z,s)+ik_y\hat{\tilde{u}}_y(\boldsymbol{k},z,s)+\partial_z\hat{\tilde{u}}_z(\boldsymbol{k},z,s)) = 0
\end{aligned}$$

with $k^2 = k_x^2 + k_y^2$. The solutions that fulfill boundary conditions at $z=0$ (i.e. $\boldsymbol{u}(x,y,0,t)=0$) are:

$$\begin{aligned}
\hat{\tilde{\boldsymbol{u}}}_1(\boldsymbol{k},z,s) &= (-k_y,k_x,0)\sinh(kz)\\
\hat{\tilde{\boldsymbol{u}}}_2(\boldsymbol{k},z,s) &= (k_x,k_y,0)\sinh(kz) + kz\frac{\tilde{\Psi}(s)+\tilde{\Phi}(s)}{\tilde{\Psi}(s)+3\tilde{\Phi}(s)}(k_x\cosh(kz),k_y\cosh(kz),-ik\sinh(kz))\\
\hat{\tilde{\boldsymbol{u}}}_3(\boldsymbol{k},z,s) &= (0,0,ik)\sinh(kz) + kz\frac{\tilde{\Psi}(s)+\tilde{\Phi}(s)}{\tilde{\Psi}(s)+3\tilde{\Phi}(s)}(k_x\sinh(kz),k_y\sinh(kz),-ik\cosh(kz))
\end{aligned}$$

And the general solution also fulfilling boundary conditions at the top of the substrate $z=h$ (i.e. $u_x(\boldsymbol{r},h,t) = u_x^h(\boldsymbol{r},t)$ and $u_y(\boldsymbol{r},h,t) = u_y^h(\boldsymbol{r},t)$) is constructed as a linear superposition:

$$\hat{\tilde{\boldsymbol{u}}}(\boldsymbol{k},z,s) = \hat{\tilde{C}}_1(\boldsymbol{k},s)\hat{\tilde{\boldsymbol{u}}}_1(\boldsymbol{k},z,s) + \hat{\tilde{C}}_2(\boldsymbol{k},s)\hat{\tilde{\boldsymbol{u}}}_2(\boldsymbol{k},z,s) + \hat{\tilde{C}}_3(\boldsymbol{k},s)\hat{\tilde{\boldsymbol{u}}}_3(\boldsymbol{k},z,s)$$

with:

$$\begin{aligned}
\hat{\tilde{C}}_1(\boldsymbol{k},s) &= \frac{k_x\hat{\tilde{u}}_y^h(\boldsymbol{k},s) - k_y\hat{\tilde{u}}_x^h(\boldsymbol{k},s)}{k^2\sinh(kh)}\\
\hat{\tilde{C}}_2(\boldsymbol{k},s) &= \frac{k_x\hat{\tilde{u}}_x^h(\boldsymbol{k},s) + k_y\hat{\tilde{u}}_y^h(\boldsymbol{k},s)}{k^2}\frac{\tilde{\Psi}(s)+3\tilde{\Phi}(s)}{\tilde{\Psi}(s)+2\tilde{\Phi}(s)}\frac{\cosh(kh)(\tilde{\Psi}(s)+2\tilde{\Phi}(s)) - kh\sinh(kh)(\tilde{\Psi}(s)+\tilde{\Phi}(s))}{\sinh(kh)\cosh(kh)(\tilde{\Psi}(s)+3\tilde{\Phi}(s)) + kh(\tilde{\Psi}(s)+\tilde{\Phi}(s))}\\
\hat{\tilde{C}}_3(\boldsymbol{k},s) &= \frac{k_x\hat{\tilde{u}}_x^h(\boldsymbol{k},s) + k_y\hat{\tilde{u}}_y^h(\boldsymbol{k},s)}{k^2}\frac{\tilde{\Psi}(s)+3\tilde{\Phi}(s)}{\tilde{\Psi}(s)+2\tilde{\Phi}(s)}\frac{\sinh(kh)\tilde{\Phi}(s) + kh\cosh(kh)(\tilde{\Psi}(s)+\tilde{\Phi}(s))}{\sinh(kh)\cosh(kh)(\tilde{\Psi}(s)+3\tilde{\Phi}(s)) + kh(\tilde{\Psi}(s)+\tilde{\Phi}(s))}
\end{aligned}$$

Finally, tractions in Fourier-Laplace transformed space are found to be, after computing the strain tensor components $\hat{\tilde{\varepsilon}}_{xz}$, $\hat{\tilde{\varepsilon}}_{yz}$, evaluating them at $z=h$ and using $\hat{\tilde{T}}_x = 2\tilde{\Phi}\hat{\tilde{\varepsilon}}_{xz}$, $\hat{\tilde{T}}_y = 2\tilde{\Phi}\hat{\tilde{\varepsilon}}_{yz}$:

$$\hat{\tilde{\boldsymbol{T}}}(\boldsymbol{k},s) = \frac{s\tilde{\Phi}(s)}{k}\begin{pmatrix} k_y^2/\tanh(kh) + 2k_x^2\hat{\tilde{\gamma}}(\boldsymbol{k},s) & -k_xk_y/\tanh(kh) + 2k_xk_y\hat{\tilde{\gamma}}(\boldsymbol{k},s)\\ -k_xk_y/\tanh(kh) + 2k_xk_y\hat{\tilde{\gamma}}(\boldsymbol{k},s) & k_x^2/\tanh(kh) + 2k_y^2\hat{\tilde{\gamma}}(\boldsymbol{k},s)\end{pmatrix}\hat{\tilde{\boldsymbol{u}}}^h(\boldsymbol{k},s) \tag{12}$$

with:

$$\hat{\tilde{\gamma}}(\boldsymbol{k},s) = \frac{\cosh(kh)^2(\tilde{\Psi}(s)+2\tilde{\Phi}(s))^2 - \tilde{\Phi}(s)^2\sinh(kh)^2 + (kh)^2(\tilde{\Psi}(s)+\tilde{\Phi}(s))^2}{(\tilde{\Psi}(s)+2\tilde{\Phi}(s))(\sinh(kh)\cosh(kh)(\tilde{\Psi}(s)+3\tilde{\Phi}(s)) + kh(\tilde{\Psi}(s)+\tilde{\Phi}(s)))} \tag{13}$$

Tractions in Fourier space can also readily be written as:

$$\hat{\boldsymbol{T}}(\boldsymbol{k},t_i)=\begin{pmatrix}k_y^2 & -k_xk_y\\ -k_xk_y & k_x^2\end{pmatrix}\frac{\mathcal{L}^{-1}[s\tilde{\Phi}\hat{\tilde{\boldsymbol{u}}}^h](\boldsymbol{k},t_i)}{k\tanh(kh)}+\begin{pmatrix}k_x^2 & k_xk_y\\ k_xk_y & k_y^2\end{pmatrix}\frac{2\mathcal{L}^{-1}[s\tilde{\Phi}\hat{\tilde{\gamma}}\hat{\tilde{\boldsymbol{u}}}^h](\boldsymbol{k},t_i)}{k} \tag{14}$$

It should be noted that, analytically, the forward and inverse spatial 2D Fourier transform and time Laplace transform commute, and therefore taking $\mathcal{F}_2[\mathcal{L}[\cdot]]$ leads to the same result. However, numerically this might not be necessarily true, and in this work the numerical inverse Laplace transform is applied before the inverse discrete Fourier Transform. Considering piece-wise linear interpolation of the displacement field:

$$\boldsymbol{u}^h(\boldsymbol{r},t_i)=H(t_i)\left[\boldsymbol{u}^h(\boldsymbol{r},0)+\frac{\Delta\boldsymbol{u}_0^h(\boldsymbol{r})}{\Delta t_0}t_i\right]+\sum_{j=1}^{i-1}(t_i-t_j)H(t_i-t_j)\left[\frac{\Delta\boldsymbol{u}_j^h(\boldsymbol{r})}{\Delta t_j}-\frac{\Delta\boldsymbol{u}_{j-1}^h(\boldsymbol{r})}{\Delta t_{j-1}}\right]$$

where $\Delta\boldsymbol{u}_j^h(\boldsymbol{r})=\boldsymbol{u}^h(\boldsymbol{r},t_{j+1})-\boldsymbol{u}^h(\boldsymbol{r},t_j)$, and $\Delta t_j=t_{j+1}-t_j$. Its corresponding Fourier-Laplace transform is:

$$\mathcal{L}[\mathcal{F}_2[\boldsymbol{u}^h(\boldsymbol{r},t_i)]](\boldsymbol{k},s)=\frac{\hat{\boldsymbol{u}}^h(\boldsymbol{k},0)}{s}+\sum_{j=0}^{i-1}\frac{e^{-t_js}}{s^2}\left[\frac{\Delta\hat{\boldsymbol{u}}_j^h(\boldsymbol{k})}{\Delta t_j}-\frac{\Delta\hat{\boldsymbol{u}}_{j-1}^h(\boldsymbol{k})}{\Delta t_{j-1}}\right]$$

assuming $\Delta\boldsymbol{u}_{-1}^h=0$, and so:

$$\begin{aligned}\mathcal{L}^{-1}[s\tilde{\Phi}\hat{\tilde{\boldsymbol{u}}}^h](\boldsymbol{k},t_i)&=\mathcal{L}^{-1}\left[\hat{\boldsymbol{u}}^h(\boldsymbol{k},0)\tilde{\Phi}(s)+\sum_{j=0}^{i-1}e^{-t_js}\frac{\tilde{\Phi}(s)}{s}\left[\frac{\Delta\hat{\boldsymbol{u}}_j^h(\boldsymbol{k})}{\Delta t_j}-\frac{\Delta\hat{\boldsymbol{u}}_{j-1}^h(\boldsymbol{k})}{\Delta t_{j-1}}\right]\right](t_i)\\&=\hat{\boldsymbol{u}}^h(\boldsymbol{k},0)\Phi(t_i)+\sum_{j=0}^{i-1}\left[\frac{\Delta\hat{\boldsymbol{u}}_j^h(\boldsymbol{k})}{\Delta t_j}-\frac{\Delta\hat{\boldsymbol{u}}_{j-1}^h(\boldsymbol{k})}{\Delta t_{j-1}}\right]\int_0^{t_i-t_j}\Phi(t)dt\end{aligned}$$

Similarly:

$$\mathcal{L}^{-1}[s\hat{\tilde{\gamma}}\tilde{\Phi}\hat{\tilde{\boldsymbol{u}}}^h](\boldsymbol{k},t_i)=\hat{\boldsymbol{u}}^h(\boldsymbol{k},0)\mathcal{L}^{-1}[\hat{\tilde{\gamma}}\tilde{\Phi}](\boldsymbol{k},t_i)+\sum_{j=0}^{i-1}\left[\frac{\Delta\boldsymbol{u}_j^h(\boldsymbol{k})}{\Delta t_j}-\frac{\Delta\boldsymbol{u}_{j-1}^h(\boldsymbol{k})}{\Delta t_{j-1}}\right]\int_0^{t_i-t_j}\mathcal{L}^{-1}[\hat{\tilde{\gamma}}\tilde{\Phi}](\boldsymbol{k},t)dt \tag{15}$$

Therefore, tractions in Fourier space require computation of $\mathcal{L}^{-1}[\hat{\tilde{\gamma}}\tilde{\Phi}]$ and its integral (or $\mathcal{L}^{-1}[\hat{\tilde{\gamma}}\tilde{\Phi}/s]$), alongside the integral of Φ which is expected to be available analytically, although it can also be obtained numerically.

Closed forms for inverse Laplace Transforms appearing in veTFM

Incompressible viscoelastic materials

For materials with diverging $\tilde{\Psi}\to\infty$ as $\nu\to 0.5$, $\hat{\tilde{\gamma}}$ has an apparent indetermination which can be removed by dividing numerator and denominator by $\tilde{\Psi}$ such that the result is well-posed. This results in a $\hat{\tilde{\gamma}}$ factor that does not depend on time or the Laplace domain variable s anymore:

$$\gamma_{inc}(\boldsymbol{k})=\lim_{\nu\to 0.5}\frac{\cosh(kh)^2(1+2\tilde{\Phi}/\tilde{\Psi})^2-\tilde{\Phi}^2/\tilde{\Psi}^2\sinh(kh)+(kh)^2(1+\tilde{\Phi}/\tilde{\Psi})^2}{[1+2\tilde{\Phi}/\tilde{\Psi}][\sinh(kh)\cosh(kh)(1+3\tilde{\Phi}/\tilde{\Psi})+kh(1+\tilde{\Phi}/\tilde{\Psi})]}=\frac{\cosh(kh)^2+(kh)^2}{\sinh(kh)\cosh(kh)+kh}$$

and so:

$$\mathcal{L}^{-1}[\tilde{\Phi}\hat{\tilde{\gamma}}](\boldsymbol{k},t)=\gamma_{inc}(\boldsymbol{k})\Phi(t)\qquad(\nu\to 0.5)$$

Compressible deviatoric GMX2

For the compressible, deviatoric second order generalized Maxwell model:

$$\Phi(t) = \frac{E}{2(1+\nu)} + \frac{E_1}{2}e^{-t/\tau_1} + \frac{E_2}{2}e^{-t/\tau_2} \tag{16}$$

$$\Psi(t) = \frac{E\nu}{(1+\nu)(1-2\nu)} - \frac{2}{3}\left[\frac{E_1}{2}e^{-t/\tau_1} + \frac{E_2}{2}e^{-t/\tau_2}\right] \tag{17}$$

$\tilde{\Phi}(s)\hat{\tilde{\gamma}}(\boldsymbol{k}, s)$ can be written as simple fractions by performing partial fraction decomposition and so:

$$\mathcal{L}^{-1}[\hat{\tilde{\gamma}}\tilde{\Phi}](\boldsymbol{k}, t) = \Gamma_e(\boldsymbol{k}) + \sum_{i=1}^{6}\Gamma_i(\boldsymbol{k})e^{-\kappa_i(\boldsymbol{k})t}$$

with:

$$\begin{aligned}
\beta_1 &= 1/\tau_1 \\
\beta_2 &= 1/\tau_2 \\
\alpha_1 &= E_1(1+\nu)/E \\
\alpha_2 &= E_2(1+\nu)/E \\
\Psi_e &= E/(3(1-2\nu)) \\
\Phi_2 &= E(1+\alpha_1+\alpha_2)/(6\Psi_e(1+\nu)) \\
\Phi_1 &= E(\beta_1+\beta_2+\alpha_1\beta_2+\alpha_2\beta_1)/(6\Psi_e(1+\nu)) \\
\Phi_0 &= E\beta_1\beta_2/(6\Psi_e(1+\nu)) \\
t_4 &= \cosh(kh)^2(1+4\Phi_2)^2 - 9\sinh(kh)^2\Phi_2^2 + (kh)^2(1+\Phi_2)^2 \\
t_3 &= \cosh(kh)^2 2(1+4\Phi_2)(\beta_1+\beta_2+4\Phi_1) - 18\sinh(kh)^2\Phi_2\Phi_1 + 2(kh)^2(1+\Phi_2)(\beta_1+\beta_2+\Phi_1) \\
t_2 &= \cosh(kh)^2[2(1+4\Phi_2)(4\Phi_0+\beta_1\beta_2) + (\beta_1+\beta_2+4\Phi_1)^2] \\
&\quad - 9\sinh(kh)^2(2\Phi_2\Phi_0+\Phi_1^2) + (kh)^2[2(1+\Phi_2)(\Phi_0+\beta_1\beta_2) + (\beta_1+\beta_2+\Phi_1)^2] \\
t_1 &= 2\cosh(kh)^2(\beta_1+\beta_2+4\Phi_1)(4\Phi_0+\beta_1\beta_2) - 18\sinh(kh)^2\Phi_1\Phi_0 + 2(kh)^2(\beta_1+\beta_2+\Phi_1)(\Phi_0+\beta_1\beta_2) \\
t_0 &= \cosh(kh)^2(4\Phi_0+\beta_1\beta_2)^2 - 9\sinh(kh)^2\Phi_0^2 + (kh)^2(\Phi_0+\beta_1\beta_2)^2 \\
p_6 &= (1+\alpha_1+\alpha_2)t_4 \\
p_5 &= (1+\alpha_1+\alpha_2)t_3 + (\beta_1+\beta_2+\alpha_1\beta_2+\alpha_2\beta_1)t_4 \\
p_4 &= (1+\alpha_1+\alpha_2)t_2 + (\beta_1+\beta_2+\alpha_1\beta_2+\alpha_2\beta_1)t_3 + \beta_1\beta_2 t_4 \\
p_3 &= (1+\alpha_1+\alpha_2)t_1 + (\beta_1+\beta_2+\alpha_1\beta_2+\alpha_2\beta_1)t_2 + \beta_1\beta_2 t_3 \\
p_2 &= (1+\alpha_1+\alpha_2)t_0 + (\beta_1+\beta_2+\alpha_1\beta_2+\alpha_2\beta_1)t_1 + \beta_1\beta_2 t_2 \\
p_1 &= (\beta_1+\beta_2+\alpha_1\beta_2+\alpha_2\beta_1)t_0 + \beta_1\beta_2 t_1 \\
p_0 &= \beta_1\beta_2 t_0 \\
m_2 &= \Phi_2(7\sinh(kh)\cosh(kh)+kh) + kh + \sinh(kh)\cosh(kh) \\
m_1 &= \Phi_1(7\sinh(kh)\cosh(kh)+kh) + (\beta_1+\beta_2)(kh+\sinh(kh)\cosh(kh)) \\
m_0 &= \Phi_0(7\sinh(kh)\cosh(kh)+kh) + \beta_1\beta_2(\sinh(kh)\cosh(kh)+kh) \\
l_2 &= 1+4\Phi_2 \\
l_1 &= \beta_1+\beta_2+4\Phi_1 \\
l_0 &= 4\Phi_0+\beta_1\beta_2 \\
\kappa_1 &= \beta_1 \\
\kappa_2 &= \beta_2 \\
\kappa_3 &= -(-m_1/(2m_2) - \sqrt{(m_1/2m_2)^2 - m_0/m_2}) \\
\kappa_4 &= -(-m_1/(2m_2) + \sqrt{(m_1/2m_2)^2 - m_0/m_2}) \\
\kappa_5 &= -(-l_1/(2l_2) - \sqrt{(l_1/2l_2)^2 - l_0/l_2}) \\
\kappa_6 &= -(-l_1/(2l_2) + \sqrt{(l_1/2l_2)^2 - l_0/l_2})
\end{aligned}$$

$$\Gamma_i = -\frac{E}{2(1+\nu)}\frac{1}{m_2 l_2}\frac{\kappa_i^6 p_6 - \kappa_i^5 p_5 + \kappa_i^4 p_4 - \kappa_i^3 p_3 + \kappa_i^2 p_2 - \kappa_i p_1 + p_0}{\kappa_i \prod_{j\neq i}(\kappa_j - \kappa_i)} \qquad (i = 1,2,3,4,5,6)$$

$$\Gamma_e = \frac{E}{2(1+\nu)}\frac{1}{m_2 l_2}\frac{p_0}{\kappa_1\kappa_2\kappa_3\kappa_4\kappa_5\kappa_6}$$

The integral of this term is straightforward and can be found in the Experimental Section of the manuscript. Similarly for the GMX1 also described in this document. Note that for $\tau_1 = \tau_2 \equiv \tau$ the terms Γ_1, Γ_2 diverge and the solution is not applicable. However, for such a case $2\Phi = E_\infty + (E_1 + E_2)e^{-t/\tau}$ and tractions might be computed using the GMX1 solution with effective viscoelastic parameters $E_1' = E_1 + E_2$, $\eta_1 = \tau E_1'$, described as follows.

Compressible deviatoric standard linear solid (GMX1)

For the compressible, deviatoric first order generalized Maxwell model:

$$\Phi(t) = \frac{E}{2(1+\nu)} + \frac{E_1}{2}e^{-t/\tau_1} \tag{18}$$

$$\Psi(t) = \frac{E\nu}{(1+\nu)(1-2\nu)} - \frac{2}{3}\left[\frac{E_1}{2}e^{-t/\tau_1}\right] \tag{19}$$

Under these definitions, the deviatoric first order GMX, or standard linear solid, can be inverted similarly but in a slightly simplified manner:

$$\mathcal{L}^{-1}[\hat{\tilde{\gamma}}\tilde{\Phi}](\boldsymbol{k},t) = \frac{E}{2(1+\nu)}\left[\gamma_e(\boldsymbol{k}) + \sum_{i=1}^{3}\Gamma_i(\boldsymbol{k})\left[1 - \kappa_i(\boldsymbol{k})\left(1 + \frac{E_i(1+\nu)}{E}\right)\right]e^{-t\kappa_i(\boldsymbol{k})/\tau}\right]$$

with coefficients:

$$\alpha = E_1(1+\nu)/E$$

$$\kappa_1 = \frac{3kh + 3(3-4\nu)\cosh(kh)\sinh(kh)}{[3+\alpha(1-2\nu)]kh + [3(3-4\nu) + 7\alpha(1-2\nu)]\cosh(kh)\sinh(kh)}$$

$$\kappa_2 = \frac{3(1-\nu)}{3(1-\nu) + 2\alpha(1-2\nu)}$$

$$\kappa_3 = 1$$

$$\Gamma_1 = \frac{2[1+\nu][kh + \sinh(kh)\cosh(kh) - 2kh\cosh(kh)^2]^2}{[r - \cosh(kh)\sinh(kh)][kh + 7\cosh(kh)\sinh(kh)][kh + (3-4\nu)\cosh(kh)\sinh(kh)]}$$

$$\Gamma_2 = \frac{[1+\nu]\left[\sinh(kh)^2 - (kh)^2\right]}{4[1-\nu][kh - \cosh(kh)\sinh(kh)]}$$

$$\Gamma_3 = -\frac{7\cosh^2(kh) + (kh)^2 + 9}{4[kh + 7\cosh(kh)\sinh(kh)]}$$

$$\gamma_e = \frac{\cosh^2(kh)(3-4\nu) + (1-2\nu)^2 + (kh)^2}{2[1-\nu][\sinh(kh)\cosh(kh)(3-4\nu) + kh]}$$

Compressible non-deviatoric standard linear solid (GMX1)

For the compressible, non-deviatoric first order Generalized Maxwell model:

$$\Phi(t) = \frac{E}{2(1+\nu)} + \frac{E_1}{2}e^{-t/\tau_1} \tag{20}$$

$$\Psi(t) = \frac{E\nu}{(1+\nu)(1-2\nu)} \tag{21}$$

Similarly as to the deviatoric case, the non-deviatoric Standard Linear Solid or GMX1 can be obtained as:

$$\mathcal{L}^{-1}[\tilde{\gamma}\tilde{\Phi}](\boldsymbol{k},t) = \frac{E}{2(1+\nu)}\left[\gamma_e(\boldsymbol{k}) + \sum_{i=1}^{3}\Gamma_i(\boldsymbol{k})\left[1-\kappa_i(\boldsymbol{k})\left(1+\frac{E_1(1+\nu)}{E}\right)\right]e^{-t\kappa_i(\boldsymbol{k})/\tau}\right]$$

but this time:

$$\begin{aligned}
\alpha &= E_1(1+\nu)/E \\
\kappa_1 &= \frac{r+(3-4\nu)\cosh(kh)\sinh(kh)}{[1+\alpha(1-2\nu)]kh+[3-4\nu+\alpha(3-6\nu)]\cosh(kh)\sinh(kh)} \\
\kappa_2 &= \frac{1-\nu}{1-\nu+\alpha(1-2\nu)} \\
\kappa_3 &= 1 \\
\Gamma_1 &= \frac{\nu[\sinh(2kh)-2kh\cosh(2kh)]^2}{2[r-\cosh(kh)\sinh(kh)][kh+3\cosh(kh)\sinh(kh)][kh+(3-4\nu)\cosh(kh)\sinh(kh)]} \\
\Gamma_2 &= \frac{\nu\left[(kh)^2-\sinh(kh)^2\right]}{2[1-\nu][\cosh(kh)\sinh(kh)-kh]} \\
\Gamma_3 &= -\frac{3\cosh(kh)^2+1+(kh)^2}{2[kh+3\cosh(kh)\sinh(kh)]} \\
\gamma_e &= \frac{\cosh(kh)^2(3-4\nu)+(1-2\nu)^2+(kh)^2}{2(1-\nu)[\sinh(kh)\cosh(kh)(3-4\nu)+kh]}
\end{aligned}$$

Numerical inversion for general viscoelastic models

Given a function in Laplace domain $\tilde{f}(s)$, its Talbot inverse transform $f(t)$ is computed as:

$$f(t_i) = \frac{\chi}{Mt_i}\sum_{m=0}^{M-1}\mathrm{Re}\left[\delta_m\tilde{f}\left(\frac{s_m}{t_i}\right)\right] \tag{22}$$

with:

$$\begin{aligned}
s_m &= \chi\left(\theta_m\cot\theta_m + i\theta_m\right) \\
\delta_m &= \left(1+i(\theta_m+\cot\theta_m(\theta_m\cot\theta_m-1))\right)e^{s_m} \\
\delta_0 &= e^{\chi}/2 \\
s_0 &= \chi \\
\theta_m &= m\pi/M \\
\chi &= 10
\end{aligned}$$

Numerical inversions in this work are performed using $M = 32$. Talbot's numerical Laplace inversion requires that:

1. s_m encloses all singularities of $\tilde{f}(s)$
2. $|\tilde{f}(s)| \to 0$ uniformly as $|s| \to \infty$
3. $f(t)$ is real

$\tilde{\Phi}(s)\hat{\tilde{\gamma}}(s)$ is of $O(1/s)$ and therefore 2. is satisfied both for its direct inversion and its convolution integral form. Additionally, all poles are negative and real for GMX1 and GMX2 viscoelastic models, which can be observed when performing analytical partial fraction decomposition. Therefore

1. is also fulfilled with the considered contour. Additionally, $\hat{\tilde{\gamma}}$ depends only on the modulus of the wavevector $\boldsymbol{k}$ resulting in a real $\hat{\gamma}$, thus 3. is also satisfied. The choice of χ and other parameters defining the contour are known to have an important effect on the accuracy of numerical inversion. The value used in this work ($\chi = 10$) is suggested by Talbot in his original work and led to acceptable numerical precision compared to analytical inversion (Fig. S3). It should be noted that this choice is most probably not optimal: Abate and Whitt [4] suggest using $\chi = 5M/2$ and similarly, Weidemen [5] suggests a different parametrization of $\rho \propto M/t$, where ρ is present in the original contour described by Talbot ($s_m = \chi(\theta_m \cot\theta_m + i\rho\theta_m)$), chosen to be 1 in this work. Either way, these values are proposed in the context of arbitrary precision software, as the term e^{s_m} will rapidly overflow in double precision as M is increased. As our method involves a considerable amount of numerical inversions and arbitrary precision software is usually considerably slower when compared to double precision arithmetic, a constant χ and ρ are chosen.

S4: Computation of out of plane tractions ($\boldsymbol{T}_z$) using veTFM

Using the semi-analytical formalism, cellular normal tractions (T_z) can also be computed given the normal component of deformations (u_z) is measured, which results in prescribing the additional boundary condition $u_z(\boldsymbol{r}, h, t) = u_z^h(\boldsymbol{r}, t)$. From there, the solution proceeds similarly to the unconstrained boundary condition ($T_z = 0$), with linear superposition coefficients being for the u_z prescribed case:

$$\begin{aligned}
\hat{\tilde{C}}_1^{(c)}(\boldsymbol{k}, s) &= \frac{k_x \hat{\tilde{u}}_y^h - k_y \hat{\tilde{u}}_x^h}{k^2 \sinh(kh)} \\
\hat{\tilde{C}}_2^{(c)}(\boldsymbol{k}, s) &= \frac{k_x \hat{\tilde{u}}_x^h + k_y \hat{\tilde{u}}_y^h}{k^2} \frac{\sinh(kh)(\tilde{\Psi}(s) + 3\tilde{\Phi}(s))^2 - kh\cosh(kh)(\tilde{\Psi}(s) + \tilde{\Phi}(s))(\tilde{\Psi}(s) + 3\tilde{\Phi}(s))}{\sinh(kh)^2(\tilde{\Psi}(s) + 3\tilde{\Phi}(s))^2 - (kh)^2(\tilde{\Psi}(s) + \tilde{\Phi}(s))^2} \\
&\quad + \frac{i}{k} \frac{\hat{\tilde{u}}_z^h \sinh(kh) kh(\tilde{\Psi}(s) + \tilde{\Phi}(s))(\tilde{\Psi}(s) + 3\tilde{\Phi}(s))}{\sinh(kh)^2(\tilde{\Psi}(s) + 3\tilde{\Phi}(s))^2 - (kh)^2(\tilde{\Psi}(s) + \tilde{\Phi}(s))^2} \\
\hat{\tilde{C}}_3^{(c)}(\boldsymbol{k}, s) &= \frac{k_x \hat{\tilde{u}}_x^h + k_y \hat{\tilde{u}}_y^h}{k^2} \frac{kh\sinh(kh)(\tilde{\Psi}(s) + \tilde{\Phi}(s))(\tilde{\Psi}(s) + 3\tilde{\Phi}(s))}{\sinh(kh)^2(\tilde{\Psi}(s) + 3\tilde{\Phi}(s))^2 - (kh)^2(\tilde{\Psi}(s) + \tilde{\Phi}(s))^2} \\
&\quad - \frac{i}{k} \frac{\hat{\tilde{u}}_z^h(\tilde{\Psi}(s) + 3\tilde{\Phi}(s))(\sinh(kh)(\tilde{\Psi}(s) + 3\tilde{\Phi}(s)) + kh\cosh(kh)(\tilde{\Psi}(s) + \tilde{\Phi}(s)))}{\sinh(kh)^2(\tilde{\Psi}(s) + 3\tilde{\Phi}(s))^2 - (kh)^2(\tilde{\Psi}(s) + \tilde{\Phi}(s))^2}
\end{aligned}$$

Using these coefficients, it can be shown that tractions for u_z prescribed are, dropping dependencies for conciseness:

$$\begin{pmatrix} \hat{\tilde{T}}_x \\ \hat{\tilde{T}}_y \\ \hat{\tilde{T}}_z \end{pmatrix} = \frac{s\tilde{\Phi}(s)}{k} \begin{pmatrix} k_y^2/\tanh(kh) + 2k_x^2\hat{\tilde{\zeta}} & -k_x k_y \tanh(kh) + 2k_x k_y \hat{\tilde{\zeta}} & 2ikk_x\hat{\tilde{\zeta}}_z \\ -k_x k_y \tanh(kh) + 2k_x k_y \hat{\tilde{\zeta}} & k_x^2/\tanh(kh) + 2k_x^2\hat{\tilde{\zeta}} & 2ikk_y\hat{\tilde{\zeta}}_z \\ -2ikk_x\hat{\tilde{\zeta}}_z & -2ikk_y\hat{\tilde{\zeta}}_z & 2k^2\hat{\tilde{\zeta}}_{zz} \end{pmatrix} \begin{pmatrix} \hat{\tilde{u}}_x^h \\ \hat{\tilde{u}}_y^h \\ \hat{\tilde{u}}_z^h \end{pmatrix} \tag{23}$$

with:

$$\hat{\tilde{\zeta}}(\boldsymbol{k}, s) = (\tilde{\Psi}(s) + 2\tilde{\Phi}(s)) \frac{\sinh(kh)\cosh(kh)(\tilde{\Psi}(s) + 3\tilde{\Phi}(s)) - kh(\tilde{\Psi}(s) + \tilde{\Phi}(s))}{\sinh(kh)^2(\Psi(s) + 3\Phi(s))^2 - (kh)^2(\tilde{\Psi}(s) + \tilde{\Phi}(s))^2} \tag{24}$$

$$\hat{\tilde{\zeta}}_z(\boldsymbol{k}, s) = \frac{\sinh(kh)^2\tilde{\Phi}(s)(\tilde{\Psi}(s) + 3\tilde{\Phi}(s)) - (kh)^2(\tilde{\Psi}(s) + \tilde{\Phi}(s))^2}{\sinh(kh)^2(\tilde{\Psi}(s) + 3\tilde{\Phi}(s))^2 - (kh)^2(\tilde{\Psi}(s) + \tilde{\Phi}(s))^2} \tag{25}$$

$$\hat{\tilde{\zeta}}_{zz}(\boldsymbol{k}, s) = (\tilde{\Psi}(s) + 2\tilde{\Phi}(s)) \frac{\sinh(kh)\cosh(kh)(\tilde{\Psi}(s) + 3\tilde{\Phi}(s)) + kh(\tilde{\Psi}(s) + \tilde{\Phi}(s))}{\sinh(kh)^2(\tilde{\Psi}(s) + 3\tilde{\Phi}(s))^2 - (kh)^2(\tilde{\Psi}(s) + \tilde{\Phi}(s))^2} \tag{26}$$

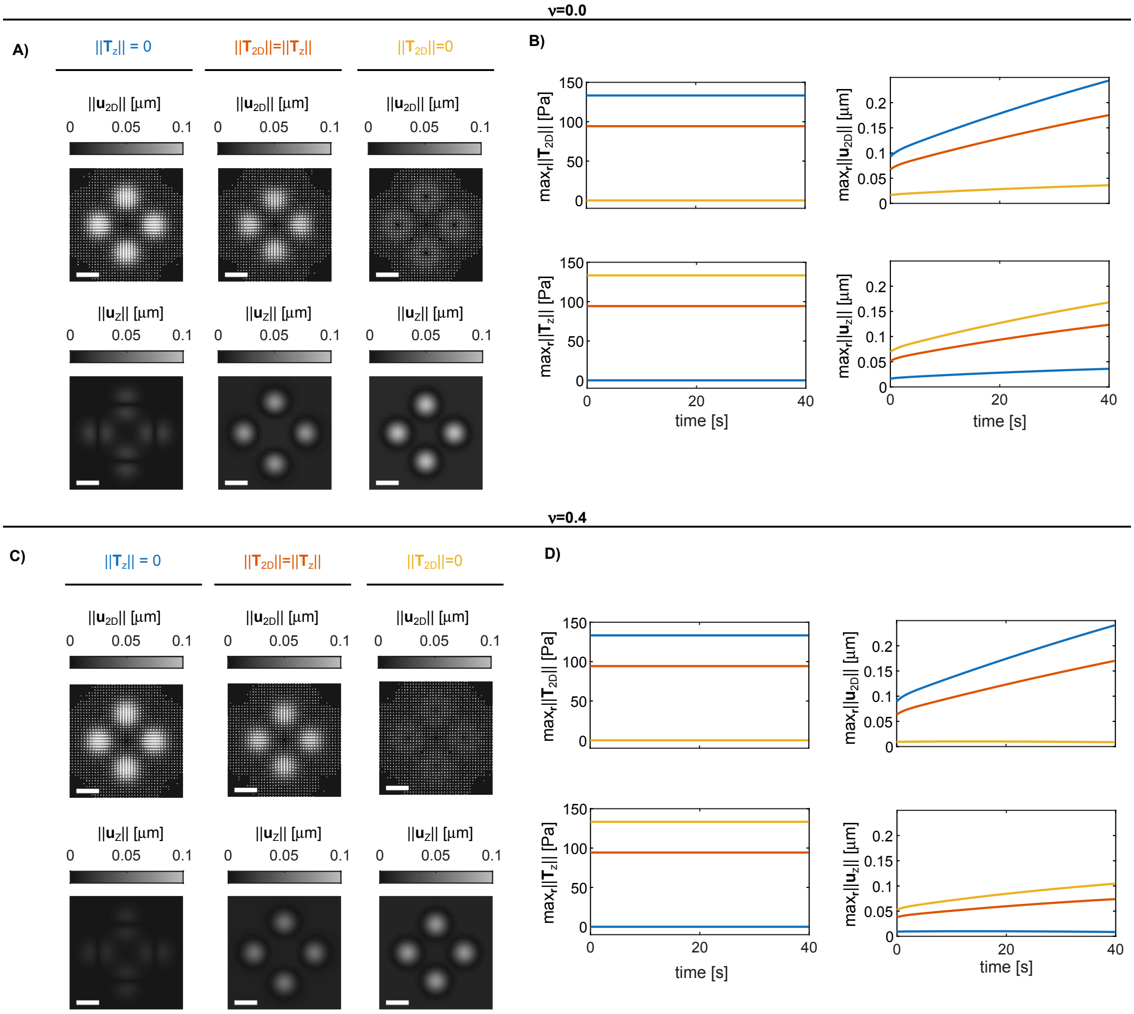

Fig. S2: Effect of out of plane traction magnitude ($\boldsymbol{T_z}$) on displacements. A, C) For $\nu = 0.0$ (A) or $\nu = 0.4$ (C) generated in-plane displacement fields ($\boldsymbol{u}_{2D}$) and out-of-plane displacement fields ($\boldsymbol{u}_z$) for cases in which $||\boldsymbol{T}_z|| = 0$ (blue), $||\boldsymbol{T}_z|| = ||\boldsymbol{T}_{2D}||$ (orange), or $||\boldsymbol{T}_{2D}|| = 0$ (yellow). **B, D)** Maximum magnitudes for $||\boldsymbol{T}_z||$, $||\boldsymbol{T}_{2D}||$, $||\boldsymbol{u}_{2D}||$ and $||\boldsymbol{u}_z||$ over time for $\nu = 0.0$ (B) and $\nu = 0.4$ (D). Scale bars indicate 10μm.

S5: Extended validation of veTFM

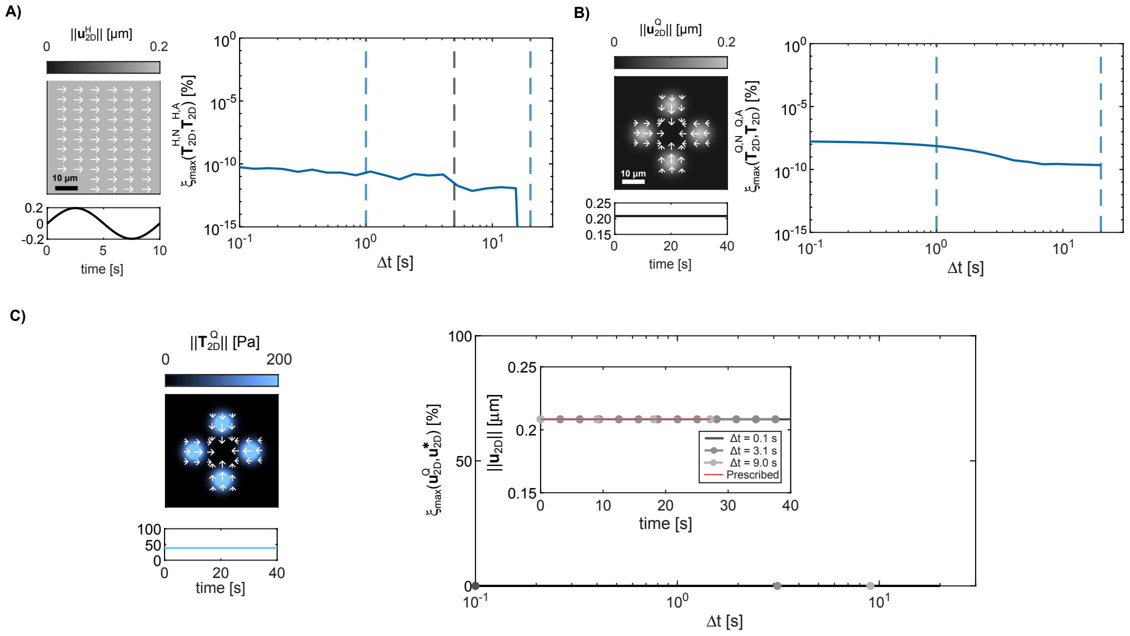

Fig. S3: Validation of the 2D veTFM solution for the two component deviatoric Generalized Maxwell Model. A, B) For a displacement field oscillating sinusoidally over time but having constant dependence over space, $\boldsymbol{u}_{2D}^{H}$ (A), or for a displacement field with gaussian spatial dependence on four localized regions but constant over time $\boldsymbol{u}_{2D}^{Q}$ (B), magnitude differences between the corresponding traction field obtained by numerical Laplace inversion ($\boldsymbol{T}_{2D}^{H/Q,N}$) and analytical Laplace inversion ($\boldsymbol{T}_{2D}^{H/Q,A}$) over different sampling times Δt. **C)** For $\boldsymbol{u}_{2D}^{Q}$, magnitude differences between the corresponding recovered displacement field obtained by numerical Laplace inversion ($\boldsymbol{u}_{2D}^{*}$) over different sampling times Δt for $\alpha_1 = \alpha_2 \approx 10^{-4}, \tau_1 = \tau_2 = 10^4 s$. Blue vertical dashed lines indicate τ_1 and τ_2, whereas the black dashed line corresponds to $1/2f$ for the harmonic case. For A) and B), a substrate with material properties $E = 700$ Pa, $\nu = 0.4$, $E_1 = 500$ Pa, $E_2 = 4000$ Pa, $\eta_1 = 500$ Pa·s, $\eta_2 = 80000$ Pa·s ($\tau_1 = 1s$, $\tau_2 = 20s$, $\alpha_1 = 0.1$, $\alpha_2 = 0.8$) is considered. For C), E and ν are maintained and E_1, E_2, η_1, η_2 are adapted accordingly. Field arrows in left panels for A, B and C) are representative - the true lattice resolution corresponds to $n_x = n_y = 41$.

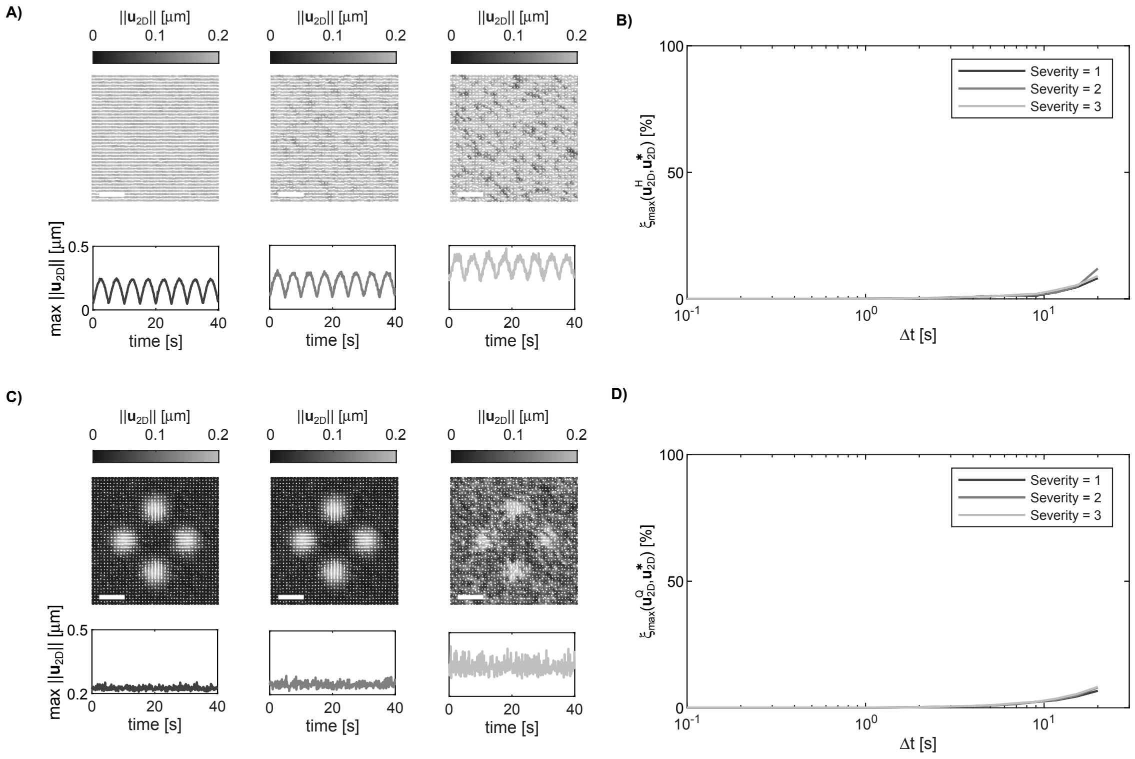

Fig. S4: Sensitivity of veTFM to noise. A, C) Harmonic $\boldsymbol{u}_{2D}^{H}$ (A) or quadrupolar $\boldsymbol{u}_{2D}^{Q}$ (C) displacement fields with addition of gaussian noise with different amplitudes. **B, D)** Maximum relative error between prescribed $\boldsymbol{u}_{2D}^{H/Q}$ and recovered displacement fields $\boldsymbol{u}_{2D}^{*}$ for the harmonic (B) or quadrupolar (D) input displacement field. Scale bars correspond to 10μm.

S6: Finite Element Modelling of viscoelastic Traction Force Microscopy

In order to compare veTFM to an independent algorithm, a linear viscoelastic Finite Element Model (FEM-veTFM) was developed. Mechanical equilibrium for the FEM can be described in a variational form by:

$$\delta W(\boldsymbol{r}, t_i) = \int_V \boldsymbol{\sigma}(\boldsymbol{r}, t_i) : \boldsymbol{\nabla}\delta\boldsymbol{r}(t_i) dV - \int_{\partial V} \boldsymbol{T}(\boldsymbol{r}, t_i) \cdot \delta\boldsymbol{r}(t_i) dA = 0 \qquad (\forall \delta\boldsymbol{r} \in H^0)$$

with $\boldsymbol{T}$ the unknown traction field, V defining the volume domain of the substrate, ∂V the surface defining the volume domain of the substrate and H^0 containing all integrable functions with integrable first derivative, vanishing at the boundary with fixed known displacements. The stress tensor in this work for the FEM implementation takes the form:

$$\boldsymbol{\sigma}(\boldsymbol{r}, t_i) = \boldsymbol{\sigma}_\infty(\boldsymbol{r}, t_i) + \sum_{k=1}^{n} \boldsymbol{\sigma}_k(\boldsymbol{r}, t_i)$$

to reduce the cost of computing the time integral in Eq. 6. For a deviatoric Generalized Maxwell Model, each of the Maxwell elements contributes to the stress as:

$$\dot{\boldsymbol{\sigma}}_k(\boldsymbol{r}, t_i) = E_k dev[\dot{\boldsymbol{\varepsilon}}(\boldsymbol{r}, t_i)] - E_k \frac{\boldsymbol{\sigma}_k(\boldsymbol{r}, t_i)}{\eta_k} \equiv \boldsymbol{g}_k(\boldsymbol{\sigma}_k, \boldsymbol{\varepsilon})$$

where $dev[\dot{\boldsymbol{\varepsilon}}(\boldsymbol{r}, t_i)] = \dot{\boldsymbol{\varepsilon}}(\boldsymbol{r}, t_i) - \boldsymbol{I}\mathrm{tr}[\dot{\boldsymbol{\varepsilon}}(\boldsymbol{r}, t_i))]/3$ and $\boldsymbol{I}$ the identity matrix. The set of ODE's in time on $\boldsymbol{\sigma}_k$ are solved numerically by means of the Runge-Kutta 4 algorithm:

$$\boldsymbol{\sigma}_k(\boldsymbol{r}, t_i) = \boldsymbol{\sigma}_k(\boldsymbol{r}, t_{i-1}) + \frac{\Delta t}{6}(\boldsymbol{g}_{k,1}(\boldsymbol{r}, t_i) + 2\boldsymbol{g}_{k,2}(\boldsymbol{r}, t_i) + 2\boldsymbol{g}_{k,3}(\boldsymbol{r}, t_i) + \boldsymbol{g}_{k,4}(\boldsymbol{r}, t_i))$$

with:

$$\begin{aligned}
\boldsymbol{g}_{k,1}(\boldsymbol{r},t_i) &= \boldsymbol{g}_k\left(\boldsymbol{\sigma}_k(\boldsymbol{r},t_{i-1}),\boldsymbol{\varepsilon}(\boldsymbol{r},t_i)\right)\\
\boldsymbol{g}_{k,2}(\boldsymbol{r},t_i) &= \boldsymbol{g}_k\left(\boldsymbol{\sigma}_k(\boldsymbol{r},t_{i-1})+\Delta t\boldsymbol{g}_{k,1}/2,\boldsymbol{\varepsilon}(\boldsymbol{r},t_i)\right)\\
\boldsymbol{g}_{k,3}(\boldsymbol{r},t_i) &= \boldsymbol{g}_k\left(\boldsymbol{\sigma}_k(\boldsymbol{r},t_{i-1})+\Delta t\boldsymbol{g}_{k,2}/2,\boldsymbol{\varepsilon}(\boldsymbol{r},t_i)\right)\\
\boldsymbol{g}_{k,4}(\boldsymbol{r},t_i) &= \boldsymbol{g}_k(\boldsymbol{\sigma}_k(\boldsymbol{r},t_{i-1})+\Delta t\boldsymbol{g}_{k,3},\boldsymbol{\varepsilon}(\boldsymbol{r},t_i))
\end{aligned}$$

Solution to the tractions fulfilling the variational problem is equivalent to finding displacements, such that, after linearization:

$$\int_V \boldsymbol{\nabla}\delta\boldsymbol{r}(t_i):\boldsymbol{c}(t_i):\boldsymbol{\varepsilon}(\boldsymbol{r}_V,t_i)dV = -\int_V \boldsymbol{\sigma}(\boldsymbol{r}_S,t_i):\boldsymbol{\nabla}\delta\boldsymbol{r}(t_i)dV + \int_{\partial V}\boldsymbol{P}(\boldsymbol{r},t_i)\cdot\delta\boldsymbol{r}(t_i)dA \qquad (\forall\delta\boldsymbol{r}\in H^0)$$

where $\boldsymbol{r}_V$ and $\boldsymbol{r}_S$ respectively are the displacements in the interior of the substrate domain (unknown) and at its boundary surface (known/prescribed). The corresponding viscoelasticity tensor is:

$$\boldsymbol{c}(t_i) = \boldsymbol{c}_\infty + \sum_k^n \boldsymbol{c}_k(t_i)$$

with:

$$\boldsymbol{c}_k(t_i) = \frac{\partial\boldsymbol{\sigma}_k(\boldsymbol{r},t_i)}{\partial\boldsymbol{\varepsilon}(\boldsymbol{r},t_i)} = \frac{\Delta t}{6}\left[\frac{\partial\boldsymbol{g}_{k,1}(\boldsymbol{r},t_i)}{\partial\varepsilon(\boldsymbol{r},t_i)} + 2\frac{\partial\boldsymbol{g}_{k,2}(\boldsymbol{r},t_i)}{\partial\boldsymbol{\varepsilon}(\boldsymbol{r},t_i)} + 2\frac{\partial\boldsymbol{g}_{k,3}(\boldsymbol{r},t_i)}{\partial\boldsymbol{\varepsilon}(\boldsymbol{r},t_i)} + \frac{\partial\boldsymbol{g}_{k,4}(\boldsymbol{r},t_i)}{\partial\boldsymbol{\varepsilon}(\boldsymbol{r},t_i)}\right]$$

Finite element discretization by use of trilinear shape functions $N_p(\boldsymbol{r})$ leads to:

$$\boldsymbol{K}(t_i)\boldsymbol{u}(\boldsymbol{r},t_i) = -\boldsymbol{F}(\boldsymbol{r},t_i)$$

The external pressure $\boldsymbol{P}$ is null in this TFM problem. Thus, for two nodes p and q belonging to an element e, the elemental stiffness matrix $\boldsymbol{K}_{pq}^{(e)}$ and residual forces $\boldsymbol{F}_p^{(e)}$ are defined as:

$$\boldsymbol{K}_{pq}^{(e)}(t_i) = \int_{V^{(e)}} \boldsymbol{B}_p^T\boldsymbol{D}(t_i)\boldsymbol{B}_q dV$$

$$\boldsymbol{F}_p^{(e)}(t_i) = \int_{V^{(e)}} \boldsymbol{B}_p^T\boldsymbol{\sigma}(\boldsymbol{r}_S,t_i)dV = -\int_{\partial V^{(e)}} N_p\boldsymbol{T}(\boldsymbol{r},t_i)dA$$

where $V^{(e)}$ now refers to the volume of the given finite element, $\boldsymbol{D}$ is the viscoelasticity matrix and $\boldsymbol{B}_p$ the standard deformation matrix, defined in Voigt notation as:

$$\boldsymbol{B}_p = \begin{pmatrix} \frac{\partial N_p}{dx} & 0 & 0\\ 0 & \frac{\partial N_p}{dy} & 0\\ 0 & 0 & \frac{\partial N_p}{dz}\\ \frac{\partial N_p}{dy} & \frac{\partial N_p}{dx} & 0\\ \frac{\partial N_p}{dz} & 0 & \frac{\partial N_p}{dx}\\ 0 & \frac{\partial N_p}{dz} & \frac{\partial N_p}{dy} \end{pmatrix}, \boldsymbol{D} = \frac{1}{2}\begin{pmatrix} 2c_{1111} & 2c_{1122} & 2c_{1133} & c_{1112}+c_{1121} & c_{1113}+c_{1131} & c_{1123}+c_{1132}\\ & 2c_{2222} & 2c_{2233} & c_{2212}+c_{2221} & c_{2213}+c_{2231} & c_{2223}+c_{2232}\\ & & 2c_{3333} & c_{3312}+c_{3321} & c_{3313}+c_{3331} & c_{3323}+c_{3332}\\ & & & c_{1212}+c_{1221} & c_{1213}+c_{1231} & c_{1223}+c_{1232}\\ & sym. & & & c_{1313}+c_{1331} & c_{1323}+c_{1332}\\ & & & & & c_{2323}+c_{2332} \end{pmatrix}$$

After obtaining equilibrium displacements within the substrate, tractions in the cell-substrate interface may be evaluated at a given node p in matrix notation as:

$$\boldsymbol{T}_p(t_i) = -\boldsymbol{M}_{pq}^{-1}\boldsymbol{F}_q(t_i) \tag{27}$$

with

$$\boldsymbol{M}_{pq} = \boldsymbol{I}\int_{\partial V} N_pN_q dA \tag{28}$$

S7: Assessment of pre-stress effects in viscoelastic substrates

The viscoelastic components of Φ are expected to decrease monotonically with time [6]. Therefore, viscoelastic memory arising from displacement history contributing to tractions estimation are expected to eventually be negligible. To limit the time required for such memory effects to decay in realistic experimental settings, we artificially varied the start imaging time t_0 for multiple rheological and imaging conditions, showing that the maximum relaxation time largely dictates the decay of memory effects in experimental settings (Fig. S5).

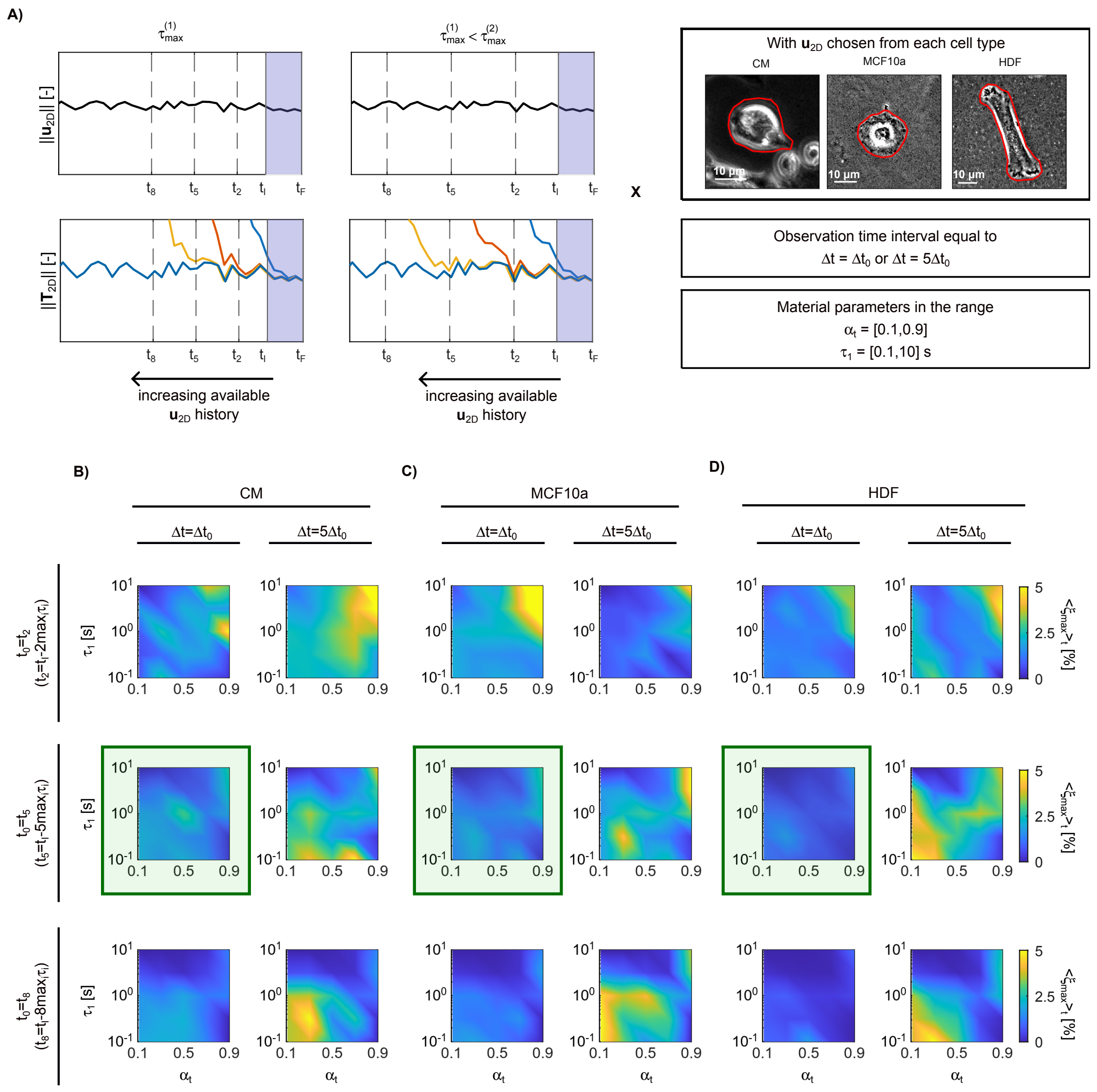

Fig. S5: Assessment of experimental pre-stress error by veTFM. A) Illustration of the proposed framework to estimate pre-stress error in viscoelastic TFM for two different maximum relaxation times $\tau_{max}^{(1)} < \tau_{max}^{(2)}$. A constant analysis time window $[t_I, t_F]$ is chosen to be as late as possible from the real initial time of imaging and therefore assumed to be representative of true tractions exerted on the substrate. The artificial initial timepoint of observation t_0 is taken to be a given time before t_I, depending on the maximum relaxation time of the material: $t_8 = t_I - 8\max_i \tau_i$, $t_5 = t_I - 5\max_i \tau_i$ or $t_2 = t_I - 2\max_i \tau_i$. This is performed for all cell types considered experimentally, and virtually repeated for different observation time intervals and material properties. **B, C, D)** Maximum relative errors for CM (B), MCF10a (C) or HDF (D) across all material properties, initial observation times t_8, t_5, t_2 and observation time intervals Δt. Highlighted in green, conditions chosen to perform experimental analysis.

S8: Elastic limits in harmonic displacement fields

Elastic limits for veTFM considering a GMX2 model can also be recovered from the viscoelastic case by letting $E_i \to 0$ such that $E_i e^{-t/\tau_i} \to 0$. Thus, for this case, viscoelastic branches do not contribute to the tractions and $\Phi \to E/(2(1+\nu))$ resulting in:

$$\gamma = \frac{\cosh(kh)^2(3-4\nu)+(1-2\nu)^2+(kh)^2}{2(1-\nu)[\sinh(kh)\cosh(kh)(3-4\nu)+kh]} \equiv \frac{\gamma_e}{2(1-\nu)}$$

which corresponds to eTFM$^\infty$ and reduces to the elastic traction force microscopy solution for corrected height [3]. Similarly, by letting $\eta_i \to \infty$, $2\Phi \to E/(1+\nu)+E_1+E_2$ which would correspond to eTFM^0. However, this analysis does not include the effect of the input displacement field. As a model accounting for this, we may consider an oscillating strain constant over space $\boldsymbol{\varepsilon}(t) = \varepsilon_0 \sin(\omega t)$ ($\omega = constant$), under which the resulting shear stress obtained from the viscoelastic constitutive equation leads to:

$$\sigma(t) = G'(\omega)\sin(\omega t)\varepsilon_0 + G''(\omega)\cos(\omega t)\varepsilon_0 \tag{29}$$

where for a GMX2:

$$G'(\omega) = E_1\frac{\omega^2\tau_1^2}{1+\omega^2\tau_2^2} + E_2\frac{\omega^2\tau_2^2}{1+\omega^2\tau_2^2} + \frac{E}{1+\nu}$$
$$G''(\omega) = E_1\frac{\omega\tau_1}{1+\omega^2\tau_1^2} + E_2\frac{\omega\tau_2}{1+\omega^2\tau_2^2}$$

The terms $\omega\tau_i$ indicate how the loading frequency on the substrate compares to the relaxation timescales of the material. Thus, for cases in which the substrate is loaded much slower than the relaxation time of the material, i.e. $\omega\tau_1$, $\omega\tau_2 \to 0$:

$$\lim_{\omega\tau_1,\omega\tau_2\to 0} G'(\omega) = \frac{E}{1+\nu}$$
$$\lim_{\omega\tau_1,\omega\tau_2\to 0} G''(\omega) = 0$$

and so for such case the substrate behaves as an elastic material with effective stiffness $E_{eff} = E_\infty = E/(1+\nu)$ (eTFM$^\infty$ limit). Instead for $\omega\tau_1$, $\omega\tau_2 \to \infty$

$$\lim_{\omega\tau_1,\omega\tau_2\to \infty} G'(\omega) = \frac{E}{1+\nu} + E_1 + E_2$$
$$\lim_{\omega\tau_1,\omega\tau_2\to \infty} G''(\omega) = 0$$

and so the system behaves as an elastic material with effective stiffness $E_{eff} = E_0 = E/(1+\nu) + E_1 + E_2$ (eTFM^0 limit). For intermediate regimes, Eq. 29 is required. In particular, at viscoelastic resonance, i.e. $\omega\tau_i = 1$:

$$\lim_{\omega\tau_1,\omega\tau_2\to 1} G'(\omega) = \frac{E}{1+\nu} + \frac{E_1}{2} + \frac{E_2}{2}$$
$$\lim_{\omega\tau_1,\omega\tau_2\to 1} G''(\omega) = \frac{E_1}{2} + \frac{E_2}{2}$$

It can be shown that the amplitude of the stress oscillations is:

$$\max(\sigma) = \varepsilon_0\sqrt{G'^2 + G''^2} \leq \varepsilon_0(G' + G'')$$

for which at the frequency maximizing the contribution of G'':

$$\max(\sigma) < \varepsilon_0(G' + G'') = \varepsilon_0\left(E_\infty + E_1 + E_2\right) = \varepsilon_0 E_0$$

and so the out of phase component diminishes the amplitude of traction oscillations with respect to the eTFM^0 limit thus reducing the effective stiffness perceived by the cell. A similar argument can be used to show that Eq. 29 is larger than E_∞ at resonance.

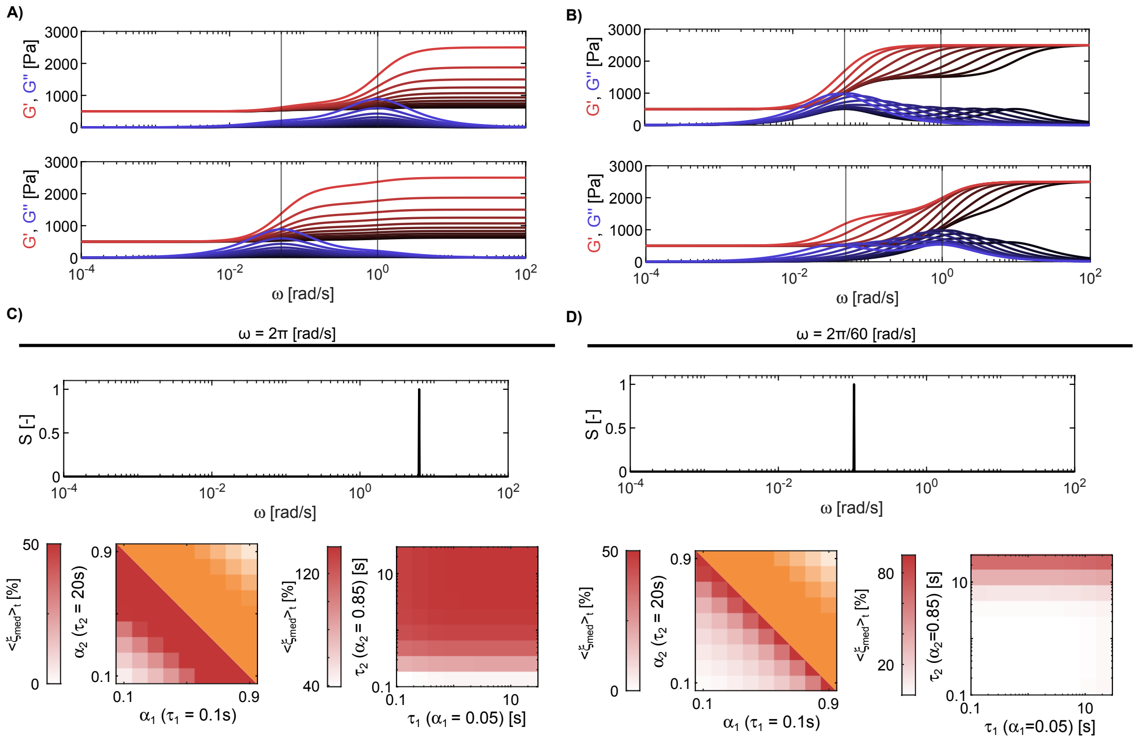

Fig. S6: Effect of angular frequency ω, α_i and τ_i on median viscoelastic harmonic traction magnitudes. **A)** Effect of varying α_1 (top) and α_2 (bottom) on G' and G'' for two distinctive τ_1, τ_2. **B)** Effect of varying τ_1 (top) and τ_2 (bottom) on G' and G'' for two distinctive α_1, α_2. **C, D)** Illustrative normalized power spectrum density for an harmonic displacement field oscillating at a frequency of 1Hz (C) or 1/60 Hz (D) (top) and corresponding median relative differences between elastic and viscoelastic harmonic tractions as a function of α_1, α_2 or τ_1, τ_2 (bottom).

S9: Strains exerted by cardiomyocytes, MCF10a cells and human dermal fibroblasts

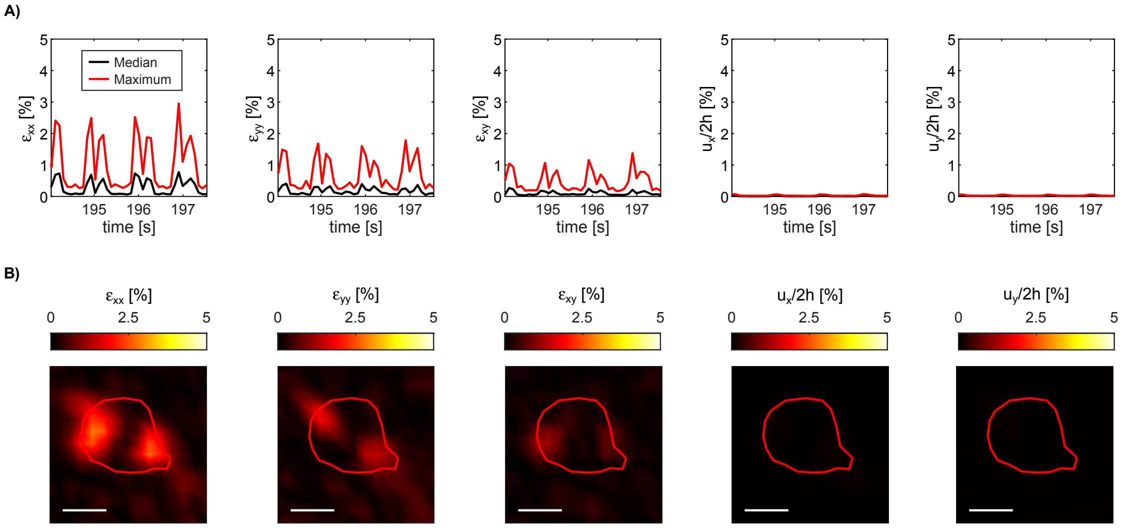

Fig. S7: Approximated strains exerted by a cardiomyocyte on a linear polyacrylamide hydrogel. **A)** Median (black) and maximum (red) strain magnitudes exerted by the cell. **B)** Corresponding strain field components for a chosen beating timepoint. ε_{xx}, ε_{yy}, ε_{xy} were obtained by central finite differences. Scale bars correspond to 10μm.

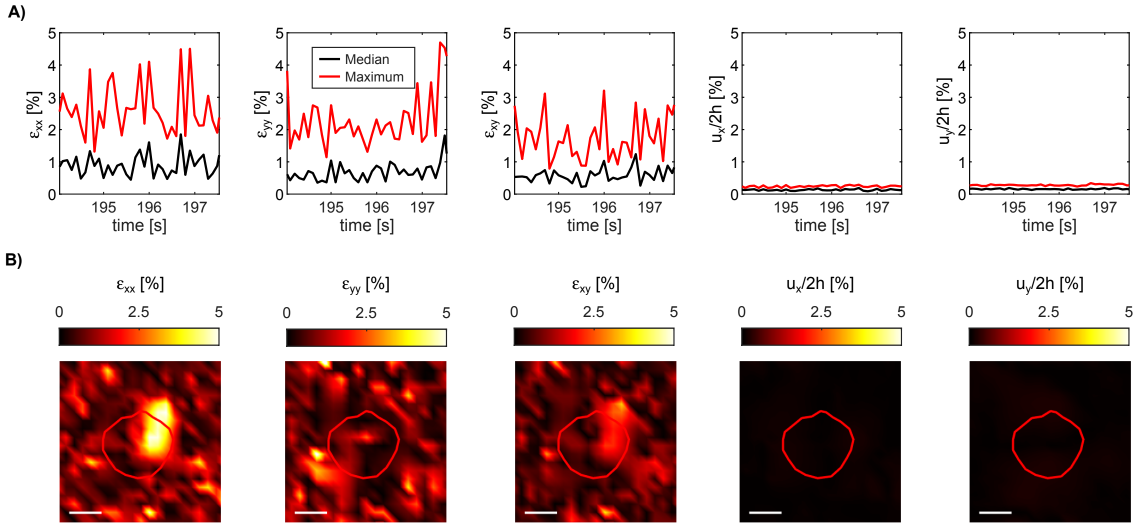

Fig. S8: Approximated strains exerted by an MCF10a cell on a linear polyacrylamide hydrogel. **A)** Median (black) and maximum (red) strain magnitudes exerted by the cell. **B)** Corresponding strain field components for a representative timepoint. ε_{xx}, ε_{yy}, ε_{xy} were obtained by central finite differences. Scale bars correspond to 10μm.

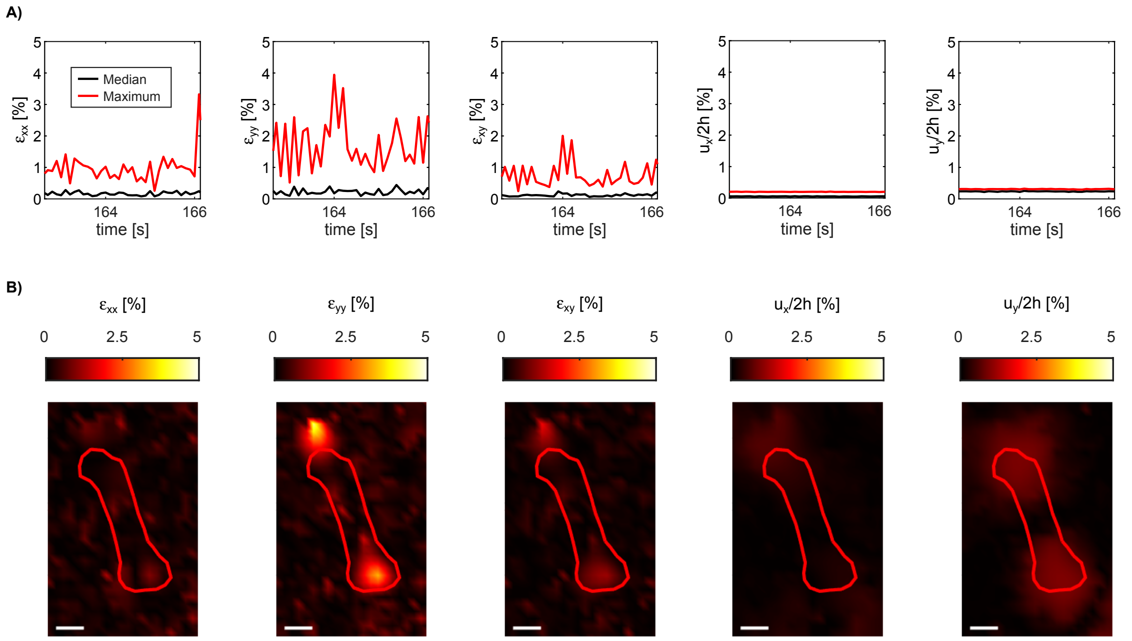

Fig. S9: Approximated strains exerted by a HDF on an alginate hydrogel. A) Median (black) and maximum (red) strain magnitudes exerted by the cell. **B)** Corresponding strain field components for a representative timepoint. ε_{xx}, ε_{yy}, ε_{xy} were obtained by central finite differences. Scale bars correspond to 10μm.

S10: Summary of experimental viscoelastic tractions

		veTFM		eTFM$^\infty$		eTFM^0	
Hydrogel	Cell type	$\underset{\boldsymbol{r},t}{med}\lVert\boldsymbol{T}_{2D}\rVert$	$\underset{\boldsymbol{r},t}{max}\lVert\boldsymbol{T}_{2D}\rVert$	$\underset{\boldsymbol{r},t}{med}\lVert\boldsymbol{T}_{2D}\rVert$	$\underset{\boldsymbol{r},t}{max}\lVert\boldsymbol{T}_{2D}\rVert$	$\underset{\boldsymbol{r},t}{med}\lVert\boldsymbol{T}_{2D}\rVert$	$\underset{\boldsymbol{r},t}{max}\lVert\boldsymbol{T}_{2D}\rVert$
**LPAA**	**CM**	20.6	83.5	19.1 (16.8%)	76.1(25.0%)	23.1(22.4%)	92.1 (24.8%)
**LPAA**	**MCF10a**	118.4	226.3	116.9 (7.7%)	205.0 (20.6%)	141.4 (20.4%)	248.0 (15.8%)
**ALG**	**HDF**	1030.8	2446.4	1027.7 (13.1%)	2200.1(100.3%)	2530.6 (63.7%)	5417.5 (60.4%)

Table 2: Summary of differences between veTFM and eTFM^0 or eTFM$^\infty$. Median and maximum traction magnitudes over space and time for each considered algorithm and cell type. In parentheses for eTFM^0 and eTFM$^\infty$, corresponding relative error with respect to veTFM. All units in Pa.

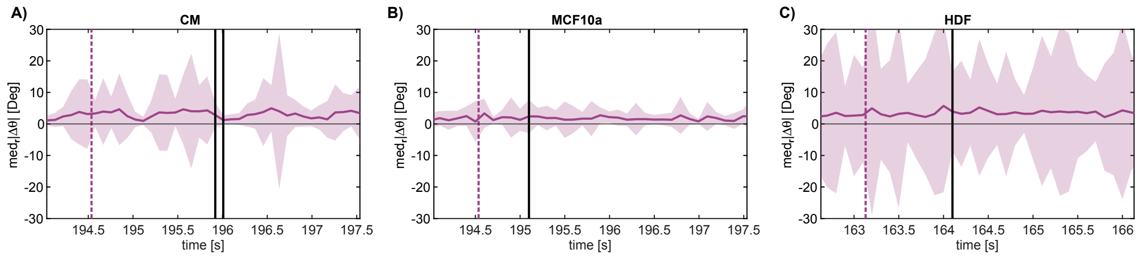

Fig. S10: Angle deviations between viscoelastic and elastic algorithms. A, B, C) For CM (A), MCF10a (B) and HDF (C) median absolute angle differences between veTFM and eTFM$^\infty$. Shaded regions corresponds to standard deviation, vertical purple lines to t_{free} and solid vertical black lines to displayed fields in Fig. 3,4,5.

References

[1] V B Nguyen et al. "Mechanical properties of single alginate microspheres determined by microcompression and finite element modelling". en. In: *Chem. Eng. Sci.* 64.5 (Mar. 2009), pp. 821–829.

[2] T Boudou et al. "An extended relationship for the characterization of Young's modulus and Poisson's ratio of tunable polyacrylamide gels". In: *Biorheology* 43.6 (Nov. 2006), pp. 721–728.

[3] Xavier Trepat et al. "Physical forces during collective cell migration". en. In: *Nat. Phys.* 5.6 (May 2009), pp. 426–430.

[4] J Abate and P P Valkó. "Multi-precision Laplace transform inversion". en. In: *Int. J. Numer. Methods Eng.* 60.5 (June 2004), pp. 979–993.

[5] J A C Weideman. "Optimizing Talbot's contours for the inversion of the Laplace transform". en. In: *SIAM J. Numer. Anal.* 44.6 (Jan. 2006), pp. 2342–2362.

[6] F Mainardi and G Spada. "Creep, relaxation and viscosity properties for basic fractional models in rheology". In: *Eur. Phys. J. Spec. Top.* 193.1 (Mar. 2011), pp. 133–160.
